# Pressure-Induced Metal-like Transport and Magnetoresistance in a Au^2+^–Au^3+^ Halide Perovskite

**DOI:** 10.1021/acscentsci.6c00315

**Published:** 2026-06-24

**Authors:** Christina R. Deschene, Armin Eghdami, Yijun Yu, Alex Smith, Zhenxian Liu, Frederick P. Marlton, Wendy L. Mao, Harold Y. Hwang, Jeffrey B. Neaton, Hemamala I. Karunadasa

**Affiliations:** † Department of Chemistry, 6429 Stanford University, Stanford, California 94305, United States; ‡ Department of Physics, 1438 University of California Berkeley, Berkeley, California 94720, United States; § Stanford Institute for Materials and Energy Sciences, SLAC National Laboratory, Menlo Park, California 94025, United States; ∥ Department of Applied Physics, 6429 Stanford University, Stanford, California 94305, United States; ⊥ Department of Physics, University of Illinois at Chicago, Chicago, Illinois 60607, United States; # Centre for Clean Energy Technology, School of Mathematical and Physical Sciences, Faculty of Science, 1994 University of Technology Sydney, Sydney, NSW 2007, Australia; ∇ Department of Earth and Planetary Sciences, 6429 Stanford University, Stanford, California 94305, United States; ○ Materials Sciences Division, 1666 Lawrence Berkeley National Laboratory, Berkeley, California 94720, United States; ◆ Kavli Energy NanoSciences Institute at Berkeley, Berkeley, California 94720, United States

## Abstract

The Cs_4_Au^II^Au^III^_2_Cl_12_ perovskite (**1**), featuring AuCl_4_ trimers separated by vacancies, enables the first high-pressure study of Au^2+/3+^mixed-valence. Our computational analysis of the gold frontier orbitals suggests that the Au^2+^→Au^3+^ intervalence charge transfer (IVCT) occurs across the vacancies. Computational structures indicate that these vacancies rapidly shrink with pressure and the Au^2+^ and Au^3+^ coordination spheres become very similar at the phase transition to nearly cubic symmetry at ca. 15 GPa—enabling facile IVCT. Although the activation energy of conductivity of 0.73(4) meV and far-infrared absorption indicate a small but nonzero bandgap, ambient thermal energy drives the IVCT, affording metallic properties: prominent infrared reflectivity and transport values of 10^2^ S·cm^–1^. This prompted us to perform the first high-pressure studies of magnetoresistance (MR) and Hall effect in halide perovskites. At 16 GPa, the MR increases by 9.3% at 2 K and 9 T; this value is maintained up to 27 GPa, when a local distortion drives electronic localization. By globally fitting the MR and Hall resistance to a two-carrier model we quantify how the carrier densities and mobilities evolve with pressure. Thus, metal-like transport and MR in **1** is driven by a pressure-induced transition from localized to partially delocalized mixed-valence.

## Graphical abstract

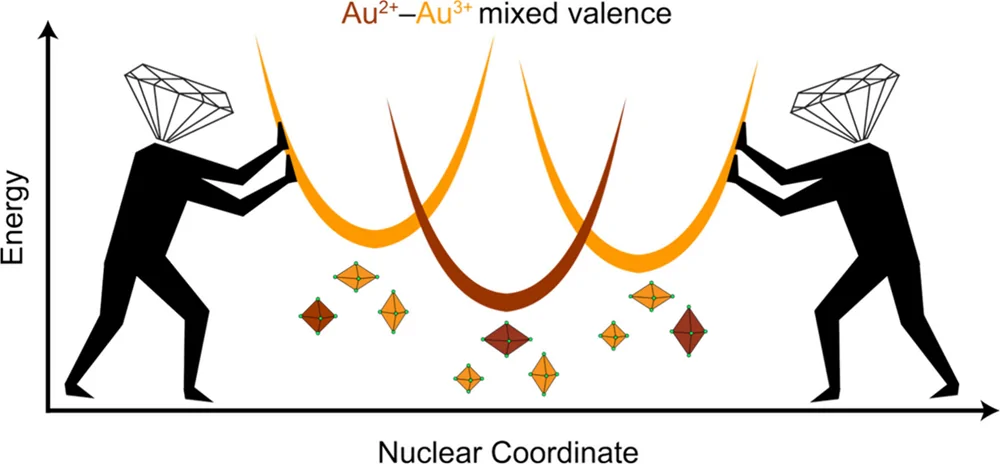

## Introduction

The recently reported 3D perovskite Cs_4_Au^II^Au^III^_2_Cl_12_ (**1**), stabilizes the exotic, magnetic Au^2+^ valence state at ambient temperature and pressure (Figure 1A).^1^ Here, a Au^2+^→Au^3+^ intervalence charge transfer (IVCT) defines the perovskite’s bandgap.^2^ The similar square planar (or tetragonally distorted octahedral) coordination of Au^2+^ and Au^3+^ (Figure 1A inset) likely affords a low energy barrier for IVCT, with an optical onset of 850(30) meV and a thermal activation energy (*E*_a_) of 600(5) meV. The stability, small bandgap, and moderate conductivity (2.58(1) × 10^–6^ S·cm^–1^ at room temperature) of **1** provide a unique opportunity to study the characteristics of the rare Au^2+^–Au^3+^ mixed-valence and magneto-electronic coupling in a conductive perovskite.

**Figure 1:**
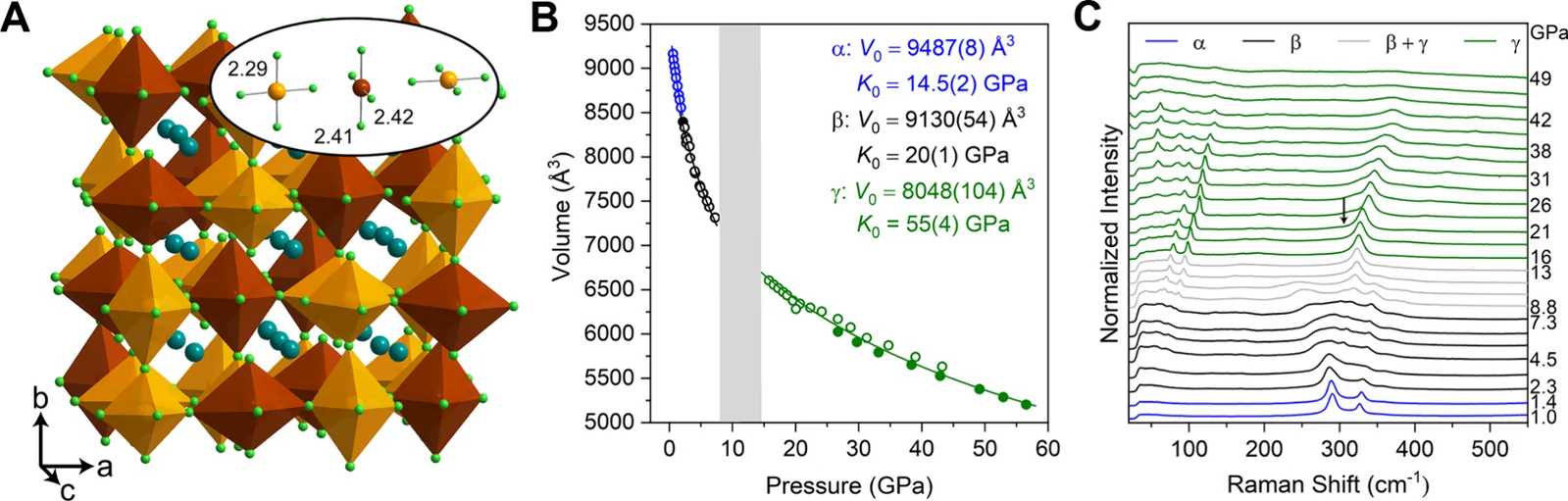
(A) Single-crystal X-ray diffraction (SC-XRD) structure of Cs_4_Au^II^Au^III^_2_Cl_12_ (**1**) at ambient pressure, adapted with permission from ref 1. Copyright 2023 Springer Nature. Teal, scarlet, gold, and green spheres represent Cs^+^, Au^2+^, Au^3+^, and Cl^–^ ions, respectively. Scarlet and gold polyhedra represent Au^II^Cl_6_ and Au^III^Cl_6_, respectively. Inset: Au–Cl bond lengths (in Å) of the Au^II^Cl_4_ and Au^III^Cl_4_ square-planar subunits within a trimer, given to 3 significant figures. (B) Evolution of the unit-cell volume of **1** with pressure, determined through phase indexing and Pawley refinement of powder X-ray diffraction (PXRD) patterns. Open and closed circles correspond to PXRD measurements collected with 400- and 300-μm culet diamonds, respectively. Second-order Birch–Murnaghan equation-of-state fits to the α (*I*4_1_ *cd*, blue), β (*Iba*2, black), and γ (*Ia*3̅*d*, green) phases are shown with corresponding bulk moduli (*K*_0_; bulk modulus at 0 GPa) and initial volumes (*V*_0_). The gray region denotes pressures where the PXRD patterns contain both β and γ phases. (C) Raman spectra of **1** with pressure collected with 785 nm excitation. Spectra corresponding to the α, β, and γ phases are assigned according to the indexed PXRD patterns at corresponding pressures. Gray spectra contain both β and γ phases. The mode denoting the distortion to lower symmetry in the γ phase beginning at 21 GPa is shown by a black arrow.

Since charge delocalization increases in mixed-valence compounds as the two metals are positioned closer together and become more similar in coordination,^2,3^ we sought to investigate the high-pressure properties of **1**. As IVCT becomes more facile in mixed-valence solids, the electronic delocalization between metals can change the solid from an insulator (Class I mixed-valence; no IVCT) to a semiconductor (Class II; thermal/optical barrier to IVCT) to a metal (Class III; no barrier to IVCT). The high crystallinity and compressibility of halide perovskites,^4,5^ make them particularly suitable for high-pressure studies. To understand high-pressure charge delocalization, however, we must track changes in carrier density and mobility with pressure, considering changes to both the average and local structure. To our knowledge, this study of the pressure dependence of Au^2+^–Au^3+^ mixed-valence marks the first high-pressure characterization of carrier densities and mobilities and coupled magneto-electronic phenomena in halide perovskites.

## I. Mixed-Valence Halide Perovskites

IVCT in mixed-valence solids often yields enhanced electronic conductivity and low-energy optical absorption, and in some cases more exotic phenomena such as high-temperature superconductivity.^6–9^ Several mixed-valence 3D halide perovskites have been isolated,^10^ with the first and most-studied being the gold double perovskites, featuring the common and nonmagnetic Au^+^ and Au^3+^ ions: A_2_Au^I^Au^III^X_6_ (A = Rb^+^, Cs^+^, K^+^, CH_3_NH_3_^+^, NH_3_^+^; X = Cl^–^, Br^–^, I^–^).^11–20^ In Class II mixed-valence perovskites, the bandgap corresponds to an IVCT.^2,21^ These IVCT bandgaps tend to be smaller than the predominantly ligand-to-metal charge-transfer (LMCT) bandgaps of Pb or Sn perovskites. Therefore, mixed-valence perovskites typically exhibit lower-energy optical absorption onsets and higher conductivity than their Pb/Sn counterparts, suggesting new applications in opto- and microelectronics.

The elpasolite double perovskites Cs_2_Au^I^Au^III^X_6_ (X = Cl^–^, Br^–^, I^–^) have been examined under pressure for decades, revealing dramatic structural and electronic transitions. These Class II mixed-valence perovskites are assigned to undergo a high-pressure Class III transition with full electronic delocalization between identical Au sites (see Supplementary Discussion 1 and Figure S1). Although the structure of **1** exhibits some similarities to that of Cs_2_Au^I^Au^III^Cl_6_, an ordered vacancy at one-quarter of the octahedral sites (B sites) in **1**, which is required to maintain the average 2+ B-site charge,^22^ disrupts the 3D connectivity of the framework (Figures 1A and S2). So, the structure of **1** is lower in symmetry than that of Cs_2_Au^I^Au^III^Cl_6_. However, **1** exhibits a smaller absorption onset and higher conductivity than does Cs_2_Au^I^Au^III^Cl_6_, which we attribute to the more similar coordination of the square planar Au^2+^ and Au^3+^ compared to the linear Au^+^ and square planar Au^3+^, affording a lower energy barrier for IVCT.

## II. Structural Response to Compression

Perovskite **1** adopts the *I*4_1_ *cd* space group at ambient conditions. This tetragonal structure (α phase) persists up to 2.2 GPa, when we observe a transition to orthorhombic symmetry (β phase, Figure S3A,D and Supplementary Discussion 2). A sluggish second phase transition begins at 8.7 GPa, and by 15 GPa, **1** appears to complete the transition to cubic symmetry (γ phase, Figure S3B). Peaks corresponding to this γ phase are present in diffraction patterns up to 57 GPa, albeit with increased broadening at the highest pressures (Figure S3A,C).

Using GSAS-II software,^23^ we indexed the α, β, and γ phases to *I*4_1_ *cd*, *Iba*2, and *Ia*3̅*d* space groups, respectively, based on observed reflections and systematic absences (Figure S4, see Supplementary Discussion 2). However, other space-group/unit-cell assignments are possible for the β and γ phases. We used Pawley refinement to fit the diffraction patterns and extract unit-cell parameters and volumes (Figure 1B, Figure S5, Tables S1–3). Notably, the *Ia*3̅*d* space group requires equivalent coordination of all Au sites, suggesting that **1** could exhibit Class III mixed-valence (full electronic delocalization between identical Au sites) in the γ phase. However, PXRD data fitting alone cannot detect very slight tetragonal distortions, particularly with the low intensities of data collected in diamond anvil cells and pressure-induced broadening of the reflections at high pressures.

We fit the unit-cell volume changes with pressure (in GSAS-II) using second-order Birch–Murnaghan Equations of State (BM EoS; Figure 1B, Figure S3E), which gave an ambient-pressure bulk modulus (*K*_0_; indicating degree of compressibility) of each phase. The order for the BM EoS was determined through an *F-f* plot (*F* = normalized pressure; *f* = Eulerian strain) of fit bulk moduli and volume trends (Figure S3F, see Methods).^24^ The α-phase *K*_0_ of 14.5(2) GPa is typical for 3D halide perovskites (12–18 GPa)^4^ and the γ-phase *K*_0_ of 55(4) GPa is comparable to, albeit slightly larger than, the *K*_0_ for cubic Cs_2_Au^I^Au^III^Cl_6_ (44 GPa).^25^

Because PXRD measurements only probe average unit-cell symmetry, we performed Raman spectroscopy to probe the local structure of **1** with compression (see Supplementary Discussion 3). The number of Raman modes in the α phase increases above 2 GPa, suggesting a transition to lower symmetry in the β phase (Figure 1C and S6). A similar trend occurs in the infrared modes of **1** (Figure S7). Subsequently, a decrease in the number of Raman modes at 16 GPa, suggests a transition to higher symmetry in the γ phase. Overall, the vibrational spectra corroborate the assignments made through PXRD of orthorhombic symmetry in the β phase and cubic symmetry in the γ phase. However, a gradual increase in the number of Raman modes at pressures above ca. 21 GPa suggests a local symmetry-lowering structural distortion (Figure 1C, black arrow). As in other halide perovskites, all structural changes at high pressures are reversible upon decompression to ambient pressure (Figure S8).

## III. Optical Response to Compression

A Class II to III (semiconductor to metal) mixed-valence transition in an extended solid is evidenced by (1) a structural transition to crystallographically equivalent metal sites and bandgap closure, indicated by (2) complete saturation of mid-infrared (mid-IR) and far-infrared (far-IR) absorption, (3) Drude features in mid- or far-IR reflectivity, indicating the presence of free carriers, and (4) increasing conductivity with decreasing temperature.^10^

The *Ia*3̅*d* cubic space group of the γ phase (with crystallographically identical Au sites), potentially fulfills point (1). But perovskites can exhibit local distortions from average unit-cell symmetry.^26^ We performed high-pressure absorption and reflectivity measurements to look for signs of bandgap closure and free carriers in the γ phase (Figure 2). The ambient-pressure absorption onset of **1** of 850 meV reduces upon compression to fall below mid-IR energies (<80 meV) by 9.9 GPa (Figure S9). We then conducted far-IR absorption studies to determine the absorption onsets at higher pressures. The absorption onset reaches its minimum, of <20 meV, at 11 GPa (Figure 2A), then subsequently increases for all higher pressures measured (Figure 2B). The differences in the noted pressures within the γ phase between diffraction and absorption measurements can be attributed to the change in pressure transmitting media, neon gas and petroleum jelly, respectively. The far-IR absorption does not saturate in the γ phase, indicating that point (2) is not fulfilled. So, **1** retains a nonzero bandgap in the γ phase, which we attribute to the local distortion suggested by the Raman spectra.

**Figure 2:**
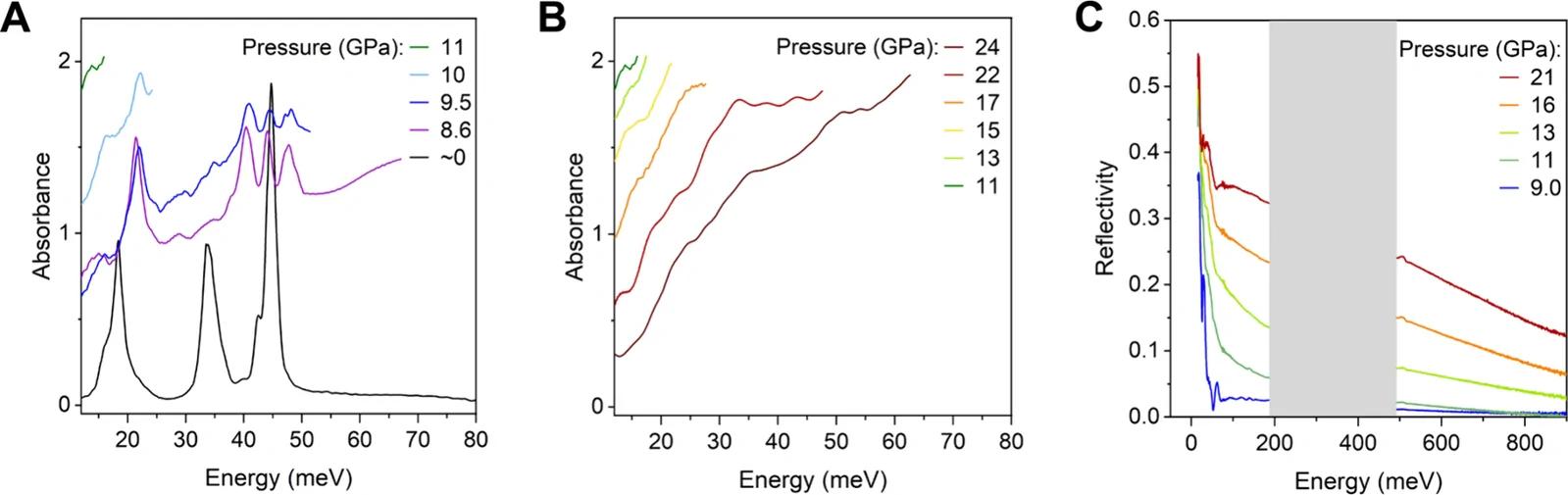
Far-infrared absorption of **1** collected (A) up to 11 GPa and (B) from 11 to 24 GPa. (C) Mid- and far-infrared reflectivity of **1** from 9.0 to 21 GPa, showing sharp increases in reflectivity below 100 meV, tentatively assigned to Drude modes. The gray region excludes absorption features from the diamond and C–H bonds in the petroleum jelly pressure-transmitting medium.

However, far-IR reflectivity measurements in the γ phase show features that appear as Drude modes, increasing in intensity from 9.0 to 21 GPa (Figure 2C) and continually present up to 52 GPa (Figure S10). As these modes are typically diagnostic of the free electronic carriers found in metals, fulfilling point (3), the conclusions from absorption and reflectivity measurements are seemingly contradictory.

## IV. Calculated Local Structure with Compression

To investigate possible local Au environments in **1** with compression, we computed ambient- and high-pressure structures using DFT using the Heyd-Scuseria-Ernzerhof (HSE) hybrid exchange-correlation functional, starting from the SC-XRD structure of **1**. Our DFT calculations used the Vienna Ab initio Simulation Package (VASP) code^27^ and are described in the initial report of **1**.^1^ We include spin-polarization but neglect spin–orbit coupling to reduce computational complexity and cost as its inclusion does not significantly alter electronic properties.^1^ Our DFT-HSE structural relaxations were performed starting with the atomic coordinates from a reduced unit cell of **1** (Figure S11). Internal coordinates, unit-cell size, and symmetry were relaxed at different pressures. While transitions to lower symmetry were not allowed in our calculations, transitions to higher symmetry (to a space group whose symmetry operations are a superset of the initial space group) were allowed. Thus, our calculations do not capture the experimentally observed transition from tetragonal to orthorhombic symmetry in the β phase.

The volume-pressure trends computed with DFT-HSE closely match those of experimental values (Figure S12C). Relaxation of the structure at zero pressure results in *I*4_1_ *cd* symmetry with average Au^3+^–Cl and Au^2+^–Cl bond lengths of 2.297(1) *Å* and 2.429(6) *Å*, respectively, in the square-planar units (Figure 3B) very similar to the experimental values of 2.289(3) *Å* and 2.416(7) *Å*, respectively (standard deviations are within parentheses). The tetragonal distortion of the unit cell is slightly smaller for the relaxed structure (*c* axis – *a* axis = 0.18 *Å*) compared to experiment (*c* axis – *a* axis = 0.32 *Å*). Since we could not obtain atomic coordinates from high-pressure PXRD data (due to limitations of the high-pressure experiments discussed previously), these variable-pressure relaxations corroborate the increasingly similar coordination of Au^2+^ and Au^3+^ in **1** with compression.

**Figure 3:**
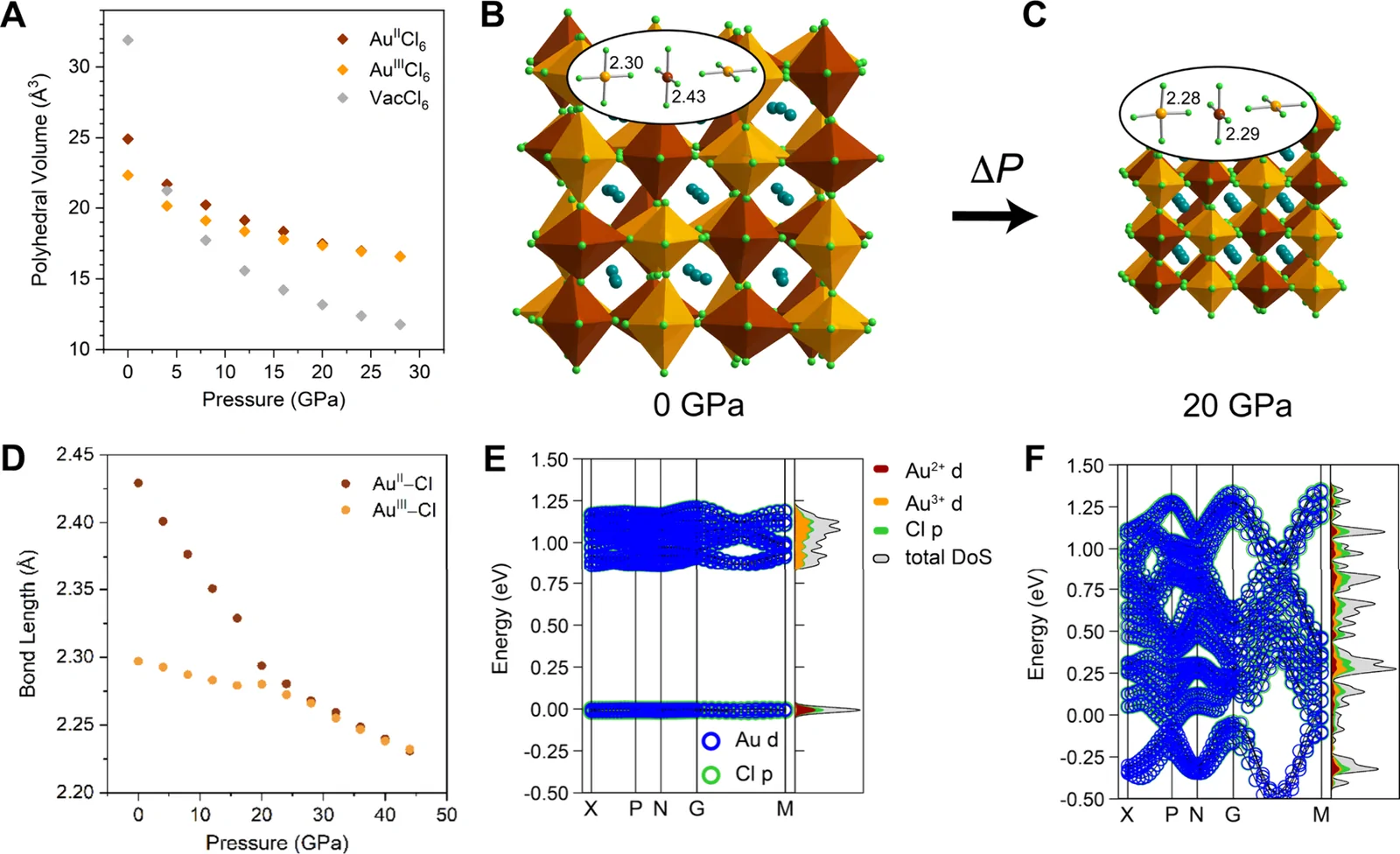
(A) Volumes of the Au^II^Cl_6_ (scarlet), Au^III^Cl_6_ (gold), and (Vac)­Cl_6_ (Vac = B-site vacancy, gray) polyhedra in calculated structural relaxations of **1** with pressure, showing preferential compression of the vacancies. Ambient-pressure SC-XRD structure of **1** after relaxation at (B) 0 GPa and (C) 20 GPa. Teal, scarlet, gold, and green spheres represent Cs^+^, Au^2+^, Au^3+^, and Cl^–^ ions, respectively. Scarlet and gold polyhedra represent Au^II^Cl_6_ and Au^III^Cl_6_, respectively. Inset denotes average bond lengths (in *Å*) of Au^II^Cl_4_ and Au^III^Cl_4_ square planar subunits, shown to 3 significant figures. (D) Average Au^II^Cl_4_ and Au^III^Cl_4_ square planar subunit bond length evolution with pressure obtained from structural relaxation. (E and F) Band structures (left) and corresponding density-of-states (right) of the structures of **1** shown in B and C, respectively. Blue and green circles denote Au d-orbital and Cl p-orbital contributions, respectively. Gold, scarlet, green, and gray shading in the density-of-states plot represent Au^3+^ d, Au^2+^ d, Cl p, and total contributions, respectively. Contributions from the individual Au valences were calculated for the Au^2+^ and Au^3+^ sites in the SC-XRD structure.

Our DFT calculations show significant preferential compression of the B-site vacancies, compared to the Au–Cl coordination spheres (Figure 3A). Within the Au coordination sphere, the largest bond-length compression occurred along the two longest, axial Au–Cl bonds (3.1–3.2 Å) perpendicular to each of the square planar AuCl_4_ units, which decreased by *ca*. 0.6 Å by 20 GPa and became more similar, resulting in approximate D_*4h*_ symmetry (Figure S12A-B). Interestingly, the Au^III^Cl_4_ square-planar subunits maintain relatively constant Au–Cl bond lengths up to 20 GPa, with only a ca. 0.02 Å decrease (Figure 3B-D, Figure S13). However, the Au^II^Cl_4_ square-planar subunits exhibited noticeable bond length shortening to become nearly equivalent to the Au^III^Cl_4_ bond lengths at 20 GPa. Notably, the calculated Au^II^–Cl and Au^III^–Cl bond lengths do not become identical, retaining the slight tetragonal unit-cell symmetry. Indeed, a diffraction pattern simulated from this relaxed structure closely matches the experimental PXRD data at 20 GPa (Figure S12D). Although the PXRD data in the γ phase can be indexed to a cubic space group (*Ia*3̅*d*) requiring equivalent Au sites (and full electronic delocalization), the calculations agree with the measured optoelectronic properties, corroborating that a slight tetragonal distortion remains at 20 GPa, leading to some degree of electronic localization.

## V. Calculated Electronic Structure with Compression

We then calculated electronic band structures and density of states of **1** at 0, 8, 16, 20, and 24 GPa with DFT-HSE (Figure 3E-F, Figure S14, Supplementary Discussion 4). At 0 GPa, **1** exhibits very flat bands with an indirect gap of 859 meV. The electronic structures at 8, 16, 20, and 24 GPa exhibit increasing band dispersion and reduced bandgaps of 543, 260, ∼ 11, and ∼ 11 meV, respectively. As expected for Class II mixed-valence, the density of states plots show that the valence band (VB) and conduction band (CB) comprise mostly Au^2+^ and Au^3+^ d orbitals, respectively, with very minor contributions from Au^3+^ and Au^2+^, respectively. There is some Cl p-orbital character in all bands. At 20 and 24 GPa, Au^2+^ and Au^3+^ d orbitals contribute nearly equally to the valence and conduction bands near the Fermi level, indicating strong electronic coupling between metal centers.

The suborbital contributions of the band extrema allow us to propose an IVCT orbital pathway for **1**. Electron density maps, projected from the *ab*- and *bc*-planes (as defined in Figure 1A) in the relaxed structures were extracted from our DFT-HSE calculations to visualize the suborbital compositions of select bands at various pressures (Figure 4E-F, Figures S15–24, Supplementary Discussion 4). By visualizing the shape of the orbitals in the electron density maps, the VB and CB are assigned to Au^2+^ d_*x* ^2^–*y* ^2^_ and Au^3+^ d_*x* ^2^–*y* ^2^_, respectively, for all pressures, with increased mixing of states at higher pressures. Notably, these orbitals are mutually orthogonal within a trimer of connected polyhedra (Figure 1A), so IVCT cannot occur within this configuration (Figure 5A-B). Therefore, to describe mixed-valence at all pressures, we invoke outer-sphere IVCT, through the overlap of Cl p-orbitals that are bonded to the Au d orbitals and are positioned at a 90° angle to one another across a vacancy (90 °Cl–Cl p-orbital interactions; Figure 5, Figure S25). Similar 90 °Cl–Cl p-orbital interactions across vacancies have been suggested by Robin and Day to explain IVCT in the mixed-valence perovskite, Cs_4_Sb^III^Sb^V^Cl_12_, which features a small bandgap despite having physically isolated Sb^3+^–Cl and Sb^5+^–Cl octahedra.^2,28^

The structure of **1** exhibits different IVCT pathways between the *ab* plane and *ac* and *bc* planes. In the *ab* plane, Au^3+^–Au^2+^–Au^3+^ across-vacancy trimers compose the smallest molecular unit for IVCT through 90 °Cl–Cl p-orbital interactions (Figures 5A,C, and S2). The trimer is asymmetric; with Cl–Cl distances between the Au^3+^–Cl and Au^2+^–Cl square planar units of 3.8 Å and 4.1 Å, indicating that the shorter-distance pathway may be more facile. In the *ac-* and *bc*-planes, two distinct Au^2+^–Au^3+^ across-vacancy dimers compose the smallest molecular units for IVCT through 90 °Cl–Cl p-orbital overlap. One features coplanar Au^2+^ d_*x* ^2^–*y* ^2^_ and Au^3+^ d_*x* ^2^–*y* ^2^_ orbitals, and the other has the Au orbitals in different planes (Figures 5C-E and S2). These across-vacancy dimers have equivalent Cl–Cl distances between the Au^3+^–Cl and Au^2+^–Cl square planar units of 3.8 Å, indicating that both pathways should contribute similarly to IVCT.

The vacancies and the axial elongation of the Au polyhedra in **1** reduce the orbital overlap for both outer-sphere IVCT pathways, resulting in weakly coupled (Class II) mixed valence at 0 GPa. As the vacancies and the long Au–Cl bonds are preferentially compressed over the shorter Au–Cl bonds with pressure (Figure 3A and S12A), this orbital overlap is greatly enhanced, resulting in greater electronic delocalization between the Au sites (Class II–III).

Consistent with the density of states plots (Figures 3E-F and S14), the electron density maps of the VBM and CBM at 0 GPa (Figures 4 and S15–24) obtained from our DFT-HSE calculations, show a VBM and CBM dominated by Au^2+^ and Au^3+^ d_*x* ^2^–*y* ^2^_, respectively, indicating very weak delocalization between the metal centers. At 20 GPa, the d_*x* ^2^–*y* ^2^_ orbitals of Au^2+^ and Au^3+^ show comparable contributions to the VB and CB, indicating considerable electronic delocalization and explaining the small experimental optical gap (<20 meV) and *E*_a_ for conductivity (<10 meV) at the γ phase transition.

**Figure 4:**
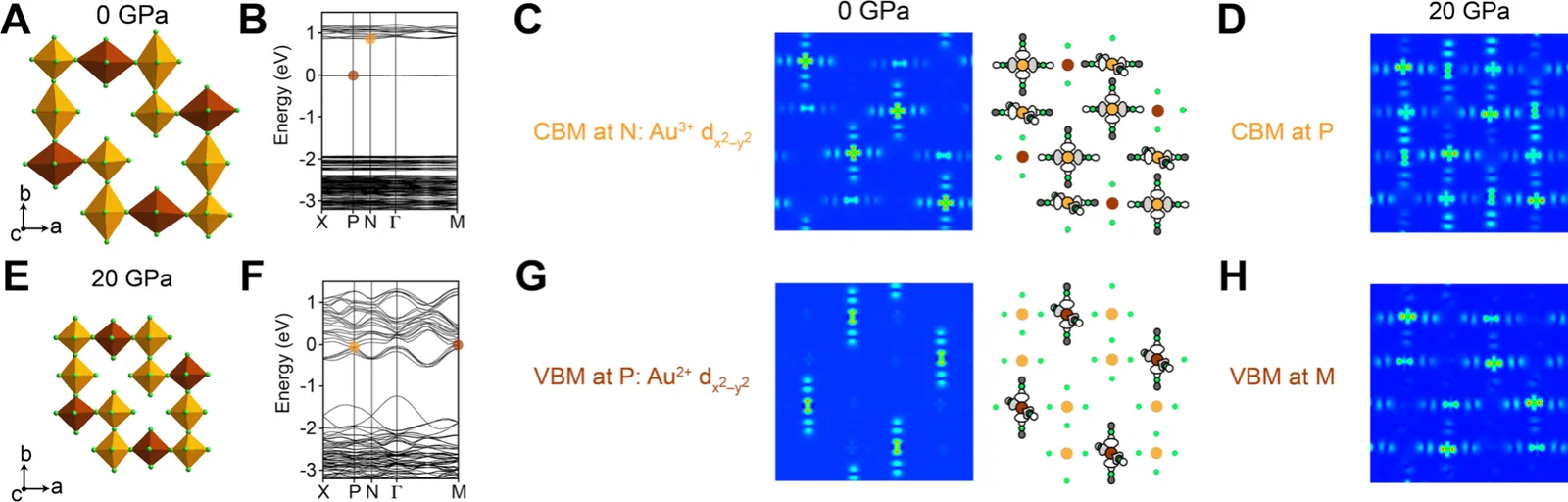
A 2D slice of **1** along the *ab*-plane after structural relaxations of the SC-XRD structure at (A) 0 GPa and (E) 20 GPa. Band structures of **1** calculated from SC-XRD relaxed at (B) 0 GPa and (F) 20 GPa. Electron density projections of the (C) conduction band minimum (CBM) and (G) valence band maximum (VBM) of the band structure of **1** at 0 GPa (shown in B), focused on the 2D slice of the structure shown in A. Corresponding orbital schematics are shown to indicate orbital assignments based on the electron density shapes. Gray and white orbital lobes represent positive and negative lobes, respectively. Scarlet, gold, and green spheres represent Au^2+^, Au^3+^, and Cl^–^ ions, respectively. Electron density projections of the (D) CBM and (H) VBM of the band structure of **1** at 20 GPa (shown in F), focused on the 2D slice of the structure shown in E. Electron density maps are shown on a rainbow scale where red is the highest and blue is the lowest density. Maps are scaled for the maximum and minimum density of each image individually, and should not be compared between images.

These maps also reflect the electronic dimensionality of **1**. Although the IVCT in the related Cs_2_Au^I^Au^III^X_6_ perovskite is localized along the *ab* plane, with 2D electronic dimensionality (Supplementary Discussion 1), **1** has VB and CB orbital contributions along all three axes, resulting in across-vacancy IVCT along all three axes and leading to isotropic electronic properties, which can be accurately probed experimentally through powder measurements.

We then computed carrier effective masses (μ) from the DFT-HSE band structures using finite-difference estimates of the band curvature near the VBM and CBM at 0, 8, 16, 20, and 24 GPa (Figure S26A, Tables S4–8). Briefly, second derivatives of the band energy at each VBM and CBM were tabulated as a 3 × 3 matrix. The average of the absolute values of the eigenvalues of this matrix correspond to 1/μ along the crystallographic *a*, *b*, and *c* axes of our computational unit cell. Although the absolute values of the extracted μ depend somewhat on the choice of *k*-point displacement in our finite difference approach, the trend of heavier hole masses, by an order of magnitude, relative to electron masses is robust across all tested step sizes (Tables S4–8). Indeed, the band structures clearly show flatter valence bands than conduction bands, particularly at low pressure. Both hole and electron effective masses decrease by ca. an order of magnitude between 0 and 20 GPa, consistent with the experimental conductivity increase with pressure. Overall, our calculations strongly indicate a significant asymmetry in carrier mobilities.

Furthermore, our DFT-HSE calculations of the magnetization at the AuCl_4_^2–^ site (assigned at 0 GPa) provide insight into the localization of the unpaired electron (also the exchanging electron for IVCT) with pressure. Consistent with the original report of **1**, the calculated magnetic moment at the AuCl_4_^2–^ units at 0 GPa is 0.88 μB (delocalized over the Au^2+^ and the coordinating Cl^–^ ions), with insignificant spin density at the AuCl_4_^1–^ units.^1^ The spin density at AuCl_4_^2–^ diminishes significantly with pressure, to 0.23 μB at 20 GPa (Figure S26B) as the initially flat Au^2+^-based band becomes much more dispersive with pressure, leading to reduced spin splitting.

## VI. Hush Diagram for IVCT

For simplicity, we first discuss IVCT in **1** with respect to the across-vacancy trimer for IVCT in the *ab* plane (Figure 5C). We start with the potential-energy surfaces for electron transfer, as described by Hush, at ambient and high pressure (Figure 6, see Figure S27 for diagrams for other pathways).^29^ The potential-energy curves are derived from diabatic surfaces, which represent the mixed-valence across-vacancy trimer before and after IVCT, set at arbitrary nuclear coordinates; these are represented in Figure 6A by the scarlet and gold curves, respectively. Note that the gold curves are not symmetric due to the unequal Cl–Cl distances in the across-vacancy trimer.

**Figure 5:**
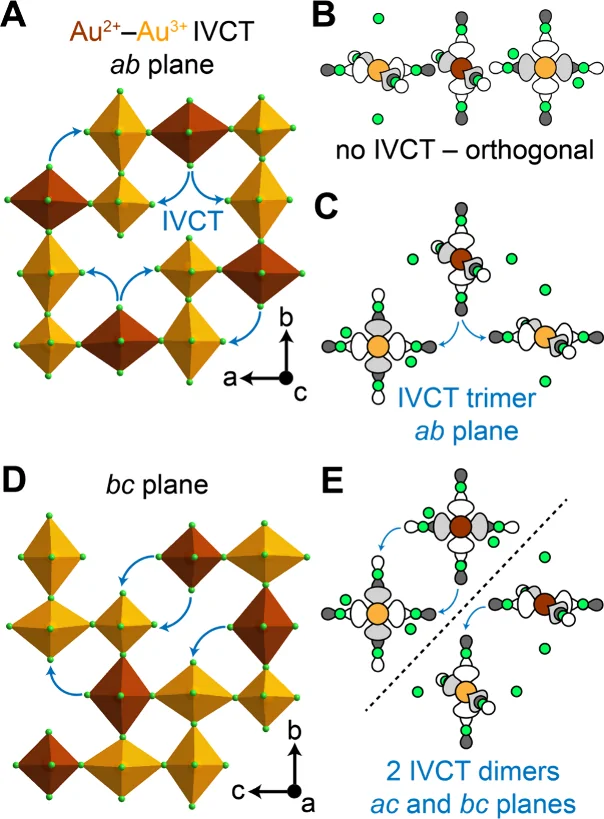
Two slices of the SC-XRD structure of **1** at ambient pressure along the (A) *ab* plane and the (D) *bc* plane (see Figure S2). Outer-sphere Au^2+^→Au^3+^ IVCT pathways through Cl p orbitals that are σ-bonded to Au d orbitals, which can occur along all three axes, are shown as blue arrows. (B–C, E) Schematics of the frontier orbital alignments within molecular units in the 2D slices in A and D. The connected trimer in (B) has mutually orthogonal frontier orbitals, preventing IVCT. (C) The across-vacancy trimer in the *ab* plane with orbital overlap for IVCT (Cl–Cl distance: 3.8 Å and 4.1 Å). (E) The two across-vacancy dimers in the *ac* and *bc* planes with orbital overlap for IVCT (Cl–Cl distance: 3.8 Å). White and gray orbital lobes denote positive and negative phases, respectively. Scarlet, gold, and green spheres represent Au^2+^, Au^3+^, and Cl^–^ ions, respectively. Scarlet and gold polyhedra represent Au^II^Cl_6_ and Au^III^Cl_6_, respectively.

**Figure 6:**
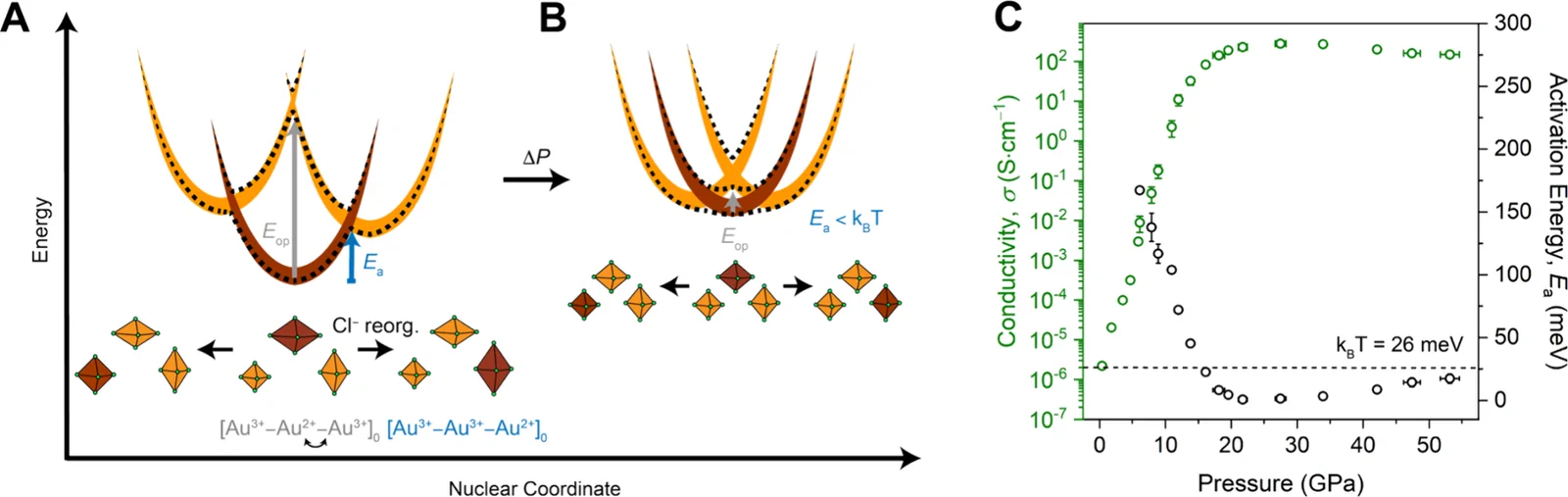
Schematic potential energy curves for intervalence charge transfer (IVCT) in a molecular trimer in **1** along the *ab* plane at (A) 0 GPa and (B) 20 GPa. Scarlet and gold curves represent the diabatic potential-energy surfaces before and after IVCT, respectively. Black dashed curves represent adiabatic surfaces created through the intersection (wave function overlap) of the diabatic surfaces. The ground-state trimer configurations of Au^II^Cl_6_ (scarlet) and Au^III^Cl_6_ (gold) polyhedra are schematics drawn to represent the Au coordination in structural relaxation at 0 GPa in A and 20 GPa in B. The gold curves are arbitrarily placed higher in energy than the scarlet curves. Blue and gray arrows represent the thermal energy barrier (*E*_a_) and the optical energy barrier (*E*_op_) for IVCT, respectively. (C) Conductivity at 300 K (green) and *E*_a_ for conductivity (black) of **1** with pressure. The *E*_a_ values were determined through Arrhenius fits from 250 to 300 K for each pressure.

Wave function mixing of the diabatic states at their intersection results in the formation of three adiabatic surfaces, depicted by the black dashed curves in Figure 6. The bottom and middle surfaces are split by an energy corresponding to the scale of electronic coupling between the middle and terminal Au ions.^30,31^ As there is little mixing of Au^2+^ and Au^3+^ states in the calculated band edges of **1**, we expect weak electronic delocalization and a small splitting of the adiabatic surfaces. These surfaces dictate the experimentally observed optical and thermal IVCT energy barriers. The optical IVCT (*E*_op_; Figure 6A), which sets the bandgap of **1**, is a vertical transition from the pre-IVCT potential-curve ground state to a vibrationally excited state in the post-IVCT potential curve.

Since our Raman and far-IR absorption data indicate a local distortion and a nonzero bandgap in the cubic γ phase, respectively, we propose that, with compression, the coordination environments of Au^2+^ and Au^3+^ become extremely similar, but not identical, resulting in very close nuclear coordinates for the across-vacancy trimer before and after IVCT (Figure 6B), explaining the decreasing optical gap with pressure.

## VII. Electronic Response to Compression

In addition to optical IVCT, thermal excitation can also promote electron transfer in **1**. The energy barrier for this horizontal transition between adiabatic minima corresponds to the activation energy of conductivity (*E*_a_). Symmetric IVCT should have a *E*_a_:*E*_op_ ratio of 1:4. The experimental *E*_a_:*E*_op_ ratio of 1:1.4 indicates asymmetric IVCT at ambient pressure as expected for the Au^3+^–Au^2+^–Au^3+^→Au^2+^–Au^3+^–Au^3+^ transition, with distinguishable initial and final states and differing Cl–Cl distances (Supplementary Discussion 5, Table S9).

The measured *E*_a_ value should correspond to the lowest *E*_a_ barrier in the solid. We expect the across-vacancy trimer and dimer configurations to have similar *E*_a_ barriers for the lowest-energy pathway, since the shortest Cl–Cl distance in both configurations is 3.8 Å. Notably, the *E*_a_:*E*_op_ ratio changes to 1:1.6 at 6 GPa suggesting that the IVCT becomes more symmetric with pressure, as the coordination environments of Au^2+^ and Au^3+^ become more similar.

The Drude features in the far-IR reflectivity of **1** in the γ phase—with far-IR absorption still showing a bandgap—can be understood through the evolution of *E*_a_ with pressure. The reported conductivity of **1** of 2.58(1) × 10^–6^ S·cm^–1^ increases by × 10^8^ through compression to 140(10) S·cm^–1^ by 18 GPa (Figure 6C), thereafter reaching its highest conductivity of 278(6) S·cm^–1^ at 27 GPa. This dramatic increase in conductivity is complemented by a sharp decrease in *E*_a_ from 600(5) meV at ambient pressure to 8.3(7) meV by 18 GPa, and reaching as low as 0.74(4) meV by 27 GPa, as determined through Arrhenius plots of variable-temperature conductivity at these pressures (Figures 6C and S28–30). Arrhenius fits in the γ phase were only performed between 250 and 300 K; at lower temperatures, the conductivity exhibited non-Arrhenius behavior, suggesting different transport pathways.

In the α and β phases, the *E*_a_ values are well above thermal energy at room temperature (∼26 meV; see Figure S29 for the hopping models used to extract the *E*_a_ in the β phase). In these pressure regions, **1** has more localized electrons and Class II mixed valence, with a thermal barrier for electronic conductivity.

At the γ phase transition, the increasingly similar coordination of Au^2+^ and Au^3+^ and their decreasing internuclear distance with compression reduces the energy and nuclear-coordinate separation of the potential surfaces (Figure 6B), significantly lowering the *E*_a_ (Figures 6C and S30). So, thermal energy at room temperature can overcome the *E*_a_, resulting in free electronic carriers, explaining the Drude features in far-IR reflectivity and high conductivity values in the γ phase, despite the nonzero bandgap. The near-disappearance of the electron transfer thermal barrier in the γ phase is consistent with a transition from Class II to II–III mixed-valence.^32^

We propose that this transition to partial delocalization, rather than complete delocalization, arises from local distortions from average symmetry, observed in Raman spectroscopy above 21 GPa, which prevent equivalent Au coordination environments and complete delocalization. Indeed, compression above 27 GPa results in slight dips in conductivity and small increases in *E*_a_, reaching values of 148(2) S·cm^–1^ and 17(4) meV, respectively, at 53(1) GPa (Figure 6C), as the local distortions observed in the Raman spectra grow stronger (Figure 1C). This local distortion is also reflected in the slight decrease of Drude feature intensity above 21 GPa and the increase in absorption onset observed above 11 GPa (Figure S10B and 2B, respectively). The difference in pressures where the effects of the local distortion is seen in IR reflectivity and optical measurements is likely due the different pressure transmitting media required in these experiments: KCl for the reflectivity data (which typically exhibits similar phase transition pressures to the Ne gas used for PXRD and Raman measurements) and petroleum jelly for the far-IR absorption (which becomes nonhydrostatic at much lower pressures).

The conductivity of **1** generally decreases with decreasing temperature, as expected for a semiconductor. Within a narrow temperature window (∼50–70 K) when the conductivity reaches its highest values at 27 and 34 GPa, the conductivity increases with decreasing temperature, showing metallic behavior (Figure S30H–I). Otherwise, **1** shows metal-like behavior: i.e., when a sufficient concentration of carriers can overcome the <1 meV bandgap from ambient thermal energy to drive IVCT. This results in some signatures of free carriers, namely Drude features in IR reflectivity spectra, despite maintaining a very small bandgap.

Overall, these data indicate that although **1** does not undergo a transition to Class III mixed valence in the γ phase, the thermal IVCT barrier drops so low in energy that it displays metal-like conductivity and IR reflectivity with substantial electronic delocalization, which is somewhat lessened through a local distortion above 21 GPa.

## VIII. Magneto-Electronic Response to Compression

Magnetoresistance (MR), or the change in electrical resistance of a material in a magnetic field, enables important applications in sensing and data storage. MR has been attributed to arise from differences in hole and electron densities and mobilities in nonmagnetic materials,^33–42^ whereas charge-carrier scattering from order/disorder transitions of magnetic spins has been invoked in magnetic materials.^43–47^ In heavy-electron magnetic materials with no long-range ordering, MR has been attributed to the Kondo effect, where strong, coherent interactions between localized spins and itinerant electrons at low temperatures typically result in positive MR.^48,49^

There are very few reports of MR in halide perovskites. Negative MR at ambient pressure and very low temperature (0.5–1 K) has been reported in heavily hole-doped CsSnI_3_, attributed to the alignment of hopping electrons with an applied magnetic field, which decreases carrier scattering and increases conductivity.^50^ Positive MR at room temperature in APbX_3_ (A = methylammonium, formamidinium; X = Cl^–^, Br^–^, I^–^) has been attributed to a decreasing ratio of triplet to singlet excitons with an applied field, and a decrease in free carriers that are primarily released from the dissociation of the triplet excitons.^51^ To our knowledge, there have been no prior reports of magnetic and conductive perovskites. The appreciable high-pressure conductivity of **1**, the presence of magnetic Au^2+^, and the different electron and hole mobilities calculated by DFT promoted us to pursue the first magnetoresistance and Hall effect measurements of a halide perovskite at high pressures.

We established Ohmic behavior of **1** (Figure S31) prior to MR and Hall resistance measurements through magnetic field sweeps at various temperatures (Figure 7, Figures S32–33). MR measurements under applied fields of up to 9 T were collected from 2 to 300 K at pressures of 11–55 GPa. High-pressure Raman measurements at 80 K and PXRD measurements from 4 to 450 K at ambient pressure suggest that the pressure-induced phase transitions of **1** are maintained at the lower temperatures of the MR measurements (Figures S34–35, see Supplementary Discussion 6). The MR dramatically increased with cooling for all pressures (Figure S32). A small low-field (∼1 T) positive MR feature occurs throughout the 2–300 K temperature range, between 6.1 and 18 GPa, disappearing at higher pressures (Figures S36A and S37–40). At temperatures below 300 K, a second stronger positive MR feature appears at higher fields (1–9 T; Figures S37–47) from 12 to 55 GPa. We did not observe hysteresis with magnetic field sweeps (Figure S39I).

**Figure 7:**
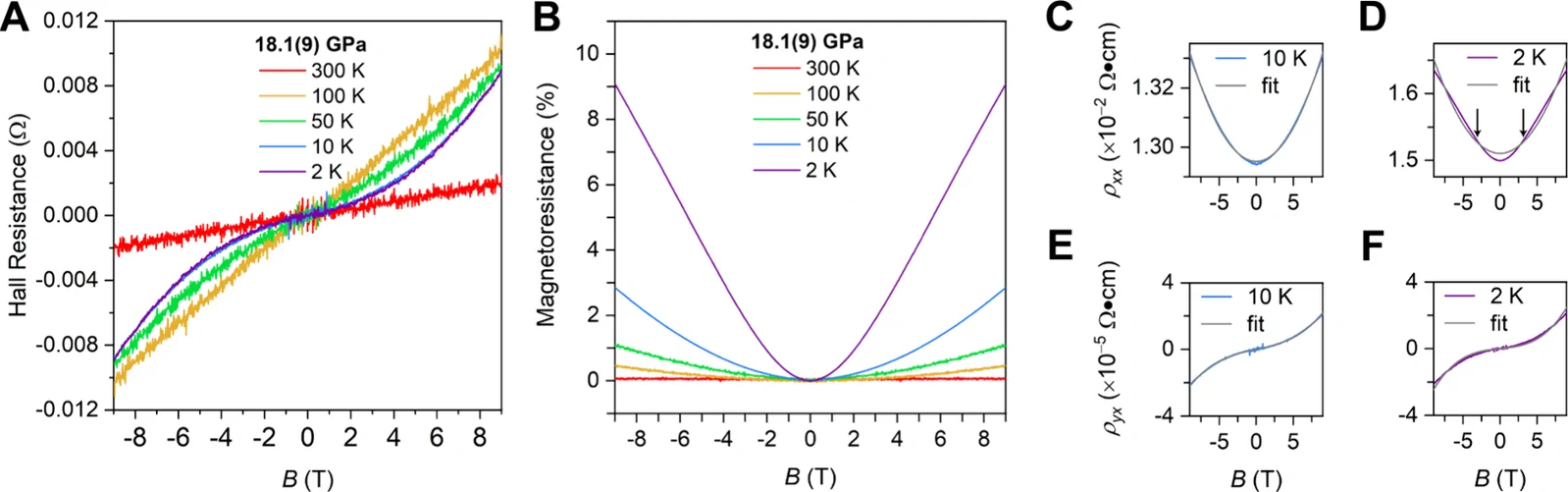
Representative (A) Hall resistance and (B) magnetoresistance (% increase) with magnetic field (*B*) of **1** at 18 GPa. (C–D) Magnetoresistivity (*ρ*_*xx*_) and (E-F) Hall resistivity (*ρ*_*yx*_) at 10 and 2 K, respectively, calculated from the data in A and B. Gray lines correspond to global fitting of *ρ*_*xx*_ and *ρ*_*yx*_ at each temperature to a two-carrier model (see text for details). Black arrows denote changes from quadratic to linear curvature of magnetoresistance in the purple trace. Red, yellow, green, blue, and purple lines correspond to measurements collected at 300, 100, 50, 10, and 2 K, respectively.

The 1-T feature is most intense at pressures below the γ-phase transition (16 GPa) when the IVCT is more localized. Low-field MR has been attributed to grain boundary effects in other materials and may cause the 1-T feature in polycrystalline **1**. First discovered in Ni/SiO_2_ granular films, MR features can arise from grain boundaries that cause packing-dependent magnetic states that alter interfacial electron transfer between particles.^52,53^ With increasing pressure in the γ phase of **1**, greater charge delocalization through more facile IVCT and the more densely packed grains should diminish these effects, potentially explaining the loss of the 1-T feature above 18 GPa.

We then interrogated the high-field MR feature in **1**. Measurements at 2 K and 9 T reach a maximum MR(%) of 9.3% at 16 GPa, the γ phase transition pressure, where the MR (%) is calculated by eq 1:

1 $$MR\left(\%\right)=\frac{MR\left(B\right)-MR\left(0\right)}{MR\left(0\right)}\times 100\%$$

Here, MR­(*B*) and MR(0) correspond to resistance values with and without an applied field, respectively. The MR(%) maxima plateau at higher pressures and subsequently decrease beginning at 20 GPa, near the pressure of the local distortion in **1** (Figure S36B).

Based on the calculated band structures in the vicinity of the Fermi level (Figure 3F) and the apparent inadequacy of a single-carrier model to fit the data, we interpret the magnetotransport behavior within a two-carrier framework, while, for the moment, neglecting possible magnetic contributions. This two-carrier analysis captures the overall experimental trends reasonably well. However, due to the complexity of the mixed-valence material, possible material inhomogeneity and nonhydrostaticity within the DAC, etc. it should be considered as phenomenological model that allows us to extract effective carrier densities and mobilities and track their evolution with pressure. The small activation energy implies that the two-carrier analysis is not only compatible with conventional two-band transport involving intrinsically itinerant carriers, it can also phenomenologically describe a narrow-gap semiconductor where thermally activated carriers coexist with residual carriers in defect/trap states and grain boundaries.

The Hall resistance data oscillate between showing linear (one-carrier dependence) and nonlinear (two-carrier dependence) field dependence at different pressures and temperatures. The Hall resistance increases linearly with applied field at 300 K from 14 to 22 GPa, indicating a one-carrier dependence on holes (Figure S33). At lower temperatures in this pressure regime, nonlinear positive slopes point to a hole-dominant two-carrier dependence, indicating that electrons start to play a more comparable role to holes. Above 22 GPa, the Hall resistance oscillates between showing a one- and two-carrier dependence, as well as between hole and electron dominance.

Given the calculated band structures and carrier effective masses in Section V, we simultaneously fit the 0 – 9 T Hall resistivity (*ρ*_*yx*_) and magnetoresistivity (*ρ*_*xx*_) to a two-carrier model, according to eqs 2 and 3, respectively, to extract hole (*μ*_*h*_) and electron (*μ*_*e*_) mobilities and hole (*n*_*h*_) and electron (*n*_*e*_) densities; *B* is the applied field and *e* is the electron charge (Figures 8 and S37–47).

2 $$\rho_{yx}\left(B\right)=\frac{B}{e}\frac{\left(n_{h}{\mu_{h}}^{2}-n_{e}{\mu_{e}}^{2}\right)+\left(n_{h}-n_{e}\right){\left(\mu_{e}\mu_{h}B\right)}^{2}}{{\left(n_{e}\mu_{e}+n_{h}\mu_{h}\right)}^{2}+{\left(n_{h}-n_{e}\right)}^{2}{\left(\mu_{e}\mu_{h}B\right)}^{2}}$$

3 $$\rho_{xx}\left(B\right)=\frac{1}{e}\frac{\left(n_{e}\mu_{e}+n_{h}\mu_{h}\right)+\left({n_{h}\mu }_{e}+{n_{e}\mu }_{h}\right)\mu_{e}\mu_{h}B^{2}}{{\left(n_{e}\mu_{e}+n_{h}\mu_{h}\right)}^{2}+{\left(n_{h}-n_{e}\right)}^{2}{\left(\mu_{e}\mu_{h}B\right)}^{2}}$$

Room-temperature measurements exhibited too much noise for reliable fitting across all pressures; the pressure dependence of carrier properties from 2–100 K are shown in Figure 8.

**Figure 8:**
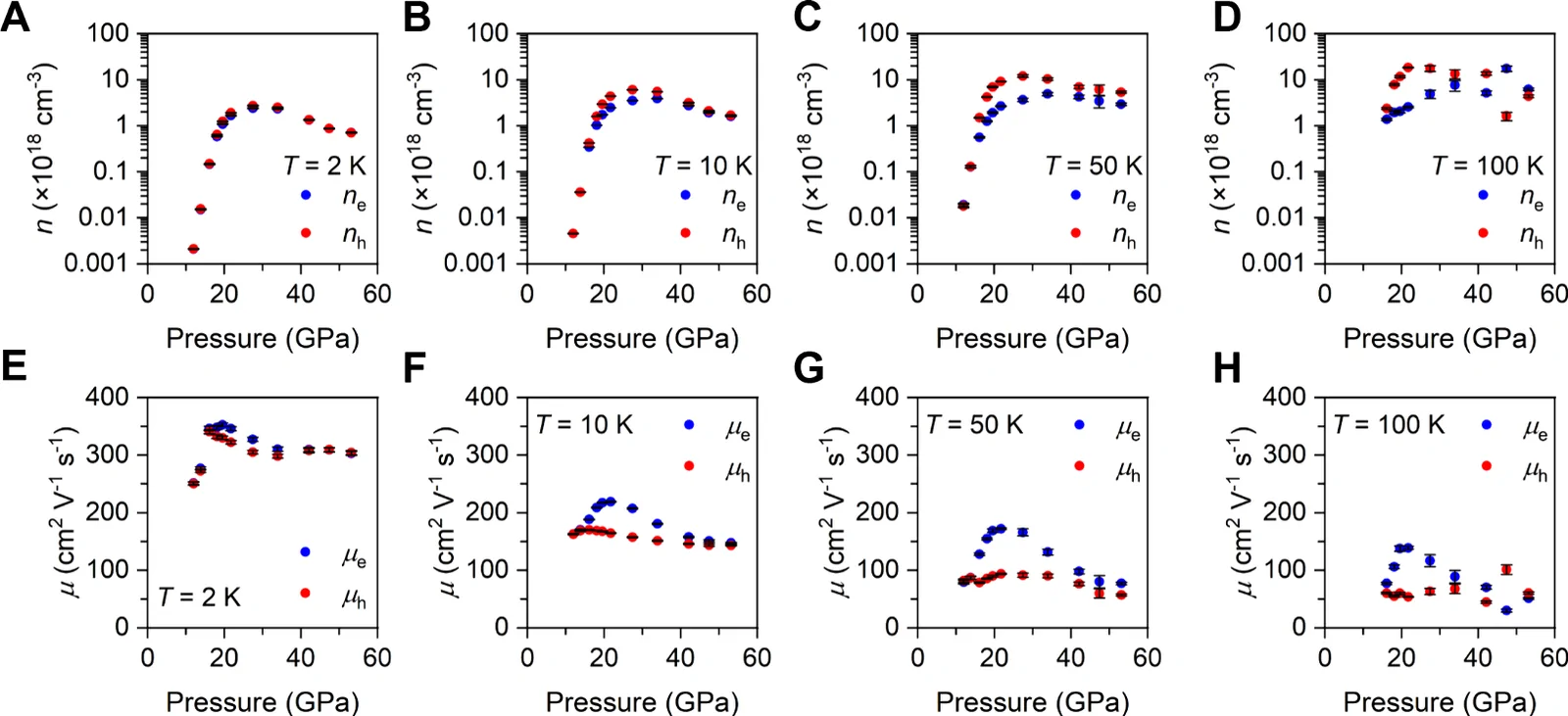
Pressure dependence of (A-D) carrier density (*n*) and (E-H) carrier mobility (*μ*) in **1** at 2, 10, 50, and 100 K, respectively, determined through global fitting of a two-carrier model to *ρ*_*yx*_ and *ρ*_*xx*_ (eqs 2 and 3, respectively) at each temperature. Subscripts *e* and *h* correspond to properties of electrons (blue) and holes (red), respectively. Error bars denote parameter uncertainties derived from simultaneous fits of the Hall and magnetoresistance data to the two-carrier model.

Between 50 and 2 K, carrier densities generally increase by × 10^3^ with compression from 12 to 27 GPa, then the carrier densities decrease slightly at 34 GPa. We attribute this behavior to the extremely small *E*_a_ for conductivity measured between 18 and 27 GPa that slightly increases at higher pressures. Since *E*_a_ drops below 10 meV between 18 and 27 GPa (and to ∼ 1 meV from 22 to 27 GPa), thermal energy is sufficient to generate a large population of free carriers, even at 2 K when *k*_B_ *T* = 0.2 meV. At 100 K (*k*_B_ *T* = 8.6 meV), carrier densities of 10^18^ cm^–3^ can be measured even at 12 GPa, when the *E*_a_ is 72 meV.

The carrier densities of electrons and holes are within an order of magnitude of each other at all pressures, with hole densities typically higher than electron densities (Figure 8A-D). These carrier densities decrease as the temperature is lowered, corroborating the semiconducting behavior indicated by the experimental *E*_a_ values for conductivity.

Between 100 and 10 K, the electron mobilities increase with pressure from 16 to 22 GPa, whereas the hole mobilities remain relatively constant (Figure 8F–H). The electron mobilities show a sharp decrease at 27 GPa, near the pressure when the local structural distortion occurs, suggesting that mobilities are more sensitive to the distortion (that affects band dispersion), compared to carrier densities, which do not begin to decrease until 34 GPa (Figure 8). Both electron and hole mobilities increase with decreasing temperature from 100 to 10 K, with higher electron mobilities than hole mobilities for almost all pressures (Figure 8F-G). The mobilities are higher still at 2 K, though the hole and electron mobilities are now much more comparable (Figure 8E). Notably, the MR(%) is largest at 16 GPa from 2 to 300 K, when the properties of holes and electrons are most different, and MR(%) decreases at higher pressures when the carrier properties become more similar. Overall, the general trend of higher mobility at lower temperatures between 100 and 2 K points to electron–phonon scattering as the dominant transport mechanism in **1**.

The two-carrier model fits the high-field MR data well between 100 and 10 K. From this analysis, we uncover opposing trends for hole and electron densities and mobilities with pressure and temperature. Hole densities are typically higher than electron densities in **1**, whereas electron mobilities are typically higher than hole mobilities at most pressures and temperatures. Therefore, we expect that the vacillating Hall resistance trends with pressure and temperature stem from electrons having higher mobilities and lower densities compared to those of holes, with different evolution with temperature and pressure. Since MR and Hall resistance directly depend on the product of carrier density and mobility, we can expect substantial variance in hole and electron dominance and in one- and two-carrier dependence at different temperatures, even at the same pressure.

Since our data from 100 to 10 K are fit reliably at high fields, the MR and Hall effect appear well-described by a two-carrier model, without considering the magnetization of the Au^2+^ in an applied field. However, the two-carrier model does not fit the high-pressure data at high fields (ca. 3–9 T) at 2 K where the curvature begins to flatten (Figure 7 D). This decrease in curvature suggests the start of a possible plateau in MR at 2 K, which will require higher fields to observe. Thus, the densities and mobilities extracted by our model are less reliable at 2 K compared to those at other temperatures. A *B* ^2^ dependence of MR requires being in the weak-field limit (ω_c_τ ≪ 1, where ω_c_ is the carrier cyclotron frequency and τ is the scattering time), and the MR of metals can show a linear dependence when they are no longer in this weak-field limit.^54^ However, we expect to be in the weak-field limit up to 9 T since the mobilities we determine require fields of >25 T to reach the strong-field limit (ω_c_τ > 1).

With the increased dispersion and reduced spin-splitting of the Au^2+^-derived bands at high pressures, the MR of **1** from 10 to 100 K may arise solely from differences in electron and hole densities and mobilities. However, even at these high pressures, the Au^2+^ spin may play a role in MR at temperatures where k_B_ *T* < *E*_a_ for conductivity. The smallest *E*_a_ measured at 27 GPa is ∼ 1 meV, whereas *k*_B_ *T* ∼ 0.2 meV at 2 K. This suggests some amount of spin localization at 2 K, as also seen from calculations at 20 GPa and 0 K (Section V).

Thus, the high-field MR feature at 2 K may potentially stem from the Kondo effect, where some amount of electron itineracy and localization coexist. The Kondo effect is typically realized in electronic structures with hybridization between flat, half-filled “impurity” bands and more dispersive conduction bands. The high-pressure band structure of **1** shows that the band with primarily Au^2+^ states at 0 GPa nearly crosses many conduction bands at 20 GPa (∼10 meV separation at 0 K) although the band dispersion is much greater than that of typical magnetic “impurity” bands in materials showing the Kondo effect (Figure 3F and S14). MR arising from the Kondo effect typically exhibits a positive, quadratic dependence to low-to-moderate fields, then a linear positive slope, followed by a negative curvature at the highest fields.^48,55^ We see a change in curvature above ∼ 3 T at 2 K for each pressure, from quadratic to linear behavior (Figures 7D and S37–47), although we do not see negative MR up to the maximum field we can measure (9 T). Given the extremely small energy scale involved, other effects—including grain-boundary effects (conduction, scattering, depletion), disorder-induced electronic localization, and shallow-defect ionization—may also contribute comparably to the MR, making a definitive interpretation difficult at present. So, specialized high-field (>9 T) and low-temperature (<2 K) measurements are required to validate this proposal.

Upon decompression, we see reversible electronic properties with some hysteresis. The conductivity at 300 K and 0 T decreases sharply between 55 and 15 GPa, then follows a similar pressure dependence to the compression data (Figure S48A). Similarly, the activation energies of conductivity are comparable to those obtained during compression at all pressures (Figures S48B and S49). Like the conductivity data, the MR(%) decreases sharply between 55 and 19 GPa, with substantially reduced values at 19 GPa compared to the value at similar pressures upon compression (Figure S50). Two-carrier fits of magnetoresistivity and Hall resistivity at 55 and 19 GPa show similar electron and hole mobilities between them, but with the carrier densities decreased by × 10 at 19 GPa (Figures S51–52). Densities at 19 GPa were × 10^2^ less than those at comparable pressures upon compression. All measured properties appeared fully reversible at pressures below 15 GPa.

## Conclusions

We performed the first studies of Au^2+^–Au^3+^ mixed-valence at high pressures with the halide perovskite: Cs_4_Au^II^Au^III^_2_Cl_12_ (**1**). At ambient pressure, tetragonal **1** exhibits Class II mixed-valence. Our calculations identify the IVCT pathway as an outer-sphere electron transfer between Au^2+^ d_*x* ^2^–*y* ^2^_ and Au^3+^ d_*x* ^2^–*y* ^2^_ via terminal chlorides that interact across the vacancies. Pressure dramatically compresses these vacancies and then makes the Au^2+^–Cl and Au^3+^–Cl coordination spheres similar, enhancing the electronic conductivity and optical absorption of **1**. At the phase transition to nearly cubic symmetry at 15 GPa, the electronic conductivity increases by × 10^8^, the optical absorption onset falls below 20 meV, and the activation energies of conductivity drop to <26 meV—below *k*_B_ *T* at room temperature. This results in large thermally activated populations of free carriers, affording metal-like behavior: prominent reflectivity features in the far-IR and conductivity values on par with those of metals. These electronic properties are consistent with a Class II–III mixed-valence assignment, with substantial but incomplete electronic delocalization between Au centers. The high-pressure conductivity of **1**, the presence of Au^2+^ as a magnetic center, and the calculated asymmetry in hole and electron effective masses led us to conduct the first high-pressure magnetoresistance and Hall effect measurements of a halide perovskite.

We observed a 9.3% increase in resistivity at 2 K with an applied field of 9 T at the high-pressure nearly cubic phase transition at 16 GPa. In this phase, we see a nonlinear field dependence of the Hall effect from 2 K to ∼ 50 K, indicating that electrons and holes contribute comparably to Hall resistance and magnetoresistance. Although the magnitude of the MR is modest, we obtain the first experimental characterization of high-pressure MR, carrier densities, and carrier mobilities in a halide perovskite and quantify their evolution with pressure. Between 12 and 27 GPa, we observed large carrier density and mobility increases with higher densities but lower mobilities for holes than those of electrons. Thus, we uncover the intricate patterns of how the carriers in a Au^2+^–Au^3+^ halide perovskite evolve with pressure as it undergoes a Class II to Class II–III mixed-valence transition with metal-like properties and coupled magnetoelectronic properties.

## Online Content

Methods, additional references, Supporting Information, acknowledgments, details of author contributions and competing interests, and statements of data and code availability are available online.

## Methods

### Experimental Details

All reagents were used as received after purchase from commercial vendors. Solvents were of reagent grade or higher purity. All measurements were collected at ambient temperature besides specified transport, Raman spectroscopy, and absorption measurements. Abbreviations used: DAC = diamond anvil cell, PXRD = powder X-ray diffraction, **1** = Cs_4_Au^II^Au^III^_2_Cl_12_.

### Synthesis of Cs_4_Au^II^Au^III^_2_Cl_12_ (**1**)

Small crystals of **1** were synthesized by the slow evaporation method in our reported procedure.^1^ This preparation was used for all studies in DACs. Solutions of 70% v/v ethanol were prepared in 20 mL batches with 14 mL of anhydrous (>99.5%) ethanol and 6 mL of deionized water. Stock solutions of the precursors for synthesis: 1 M CsCl from 168 mg (1.00 mmol) of CsCl in 1 mL 70% v/v ethanol, 0.5 M AuCl_3_ from 197 mg (0.500 mmol) HAuCl_4_·3H_2_O in 1 mL 70% v/v ethanol. In a typical synthesis, 625 μL of 1 M CsCl, 415 μL of 0.5 M AuCl_3_, and 2.5 mL 70% v/v ethanol were combined in a 5 mL scintillation vial with a plastic cap. A small amount of red precipitate formed but redissolved upon shaking. A 21 G × 1 1/2 (0.8 mm × 40 mm) needle was inserted in the cap to allow for slow evaporation and crystal formation. The vial was left in the dark for the solvent to evaporate for 10 days, after which small black crystals were visible at the bottom of the vial. These crystals were isolated from the mother liquor by filtration, washed with ∼ 10 mL of ice cold, anhydrous (>99.5%) ethanol, and dried under reduced pressure. These crystals were ground with a mortar and pestle to a powder and stored in a closed vial in a desiccator. Purity was confirmed by PXRD prior to sample chamber loadings.

For variable-temperature PXRD measurements, large quantities of powder were needed, so the above synthesis was not feasible for scale. Instead, the reported powder synthesis method in acid was used.^1^ Briefly, stock solutions were prepared of 1 M CsCl from 168 mg (1.00 mmol) of CsCl in 1 mL of 6 M HCl, 0.5 M AuCl_3_ from 197 mg (0.500 mmol) of HAuCl_4_·3H_2_O in 1 mL of 6 M HCl, and 0.1 M ascorbic acid from 35.2 mg (0.200 mmol) of l-ascorbic acid in 2 mL of 6 M HCl. The ascorbic acid stock solutions were freshly prepared on the day of the synthesis. In a typical synthesis, 375 μL of CsCl and 250 μL of AuCl_3_ stock solutions were combined in a 4 mL vial. A yellow precipitate formed immediately, identified as CsAuCl_4_. Then, 208 μL of the ascorbic acid solution was added, the solution was stirred with periodic manual agitation to ensure complete mixing, and allowed to rest for 10 min. The reaction temperature was kept at <30 °C. The dark-green solid was isolated from the mother liquor by filtration and dried under reduced pressure. A small CsAuCl_4_ impurity was observed in powder diffraction, which could be fit in Rietveld refinement to get phase fractions of 5–15% in variable-temperature PXRD.

### High-Pressure PXRD Measurements and Analysis

In-situ PXRD was performed at Beamline 12.2.2: Diffraction Under Non-Ambient Conditions at ALS at LBNL and Beamline 13-BM-C: GSECARS at APS at ANL. Using a photon energy of 25 keV, 2D Debye–Scherrer diffraction rings from powder measurements were collected on a Dectris PILATUS3 S 1 M image plate and were integrated using *Dioptas* software.^56^ The X-ray wavelength and detector distance were calibrated using a CeO_2_ diffraction standard in *Dioptas*.

Pressure calibration of PXRD measurements was done through ruby fluorescence^57^ stimulated by a 100 mW, 447 nm diode laser and measured with a Princeton Instruments Acton 300i spectrometer with fiber-optic coupling. Fluorescence measurements were collected before and after each diffraction measurement, and the average of the two was used to calculate the pressure value of the scan. The errors shown in Tables S1–3 are the difference between the collected and average values. The range of pressures were typically less than ca. 5% measurement error due to random error in shock wave experiments used in ruby fluorescence calibration.^57^ Pressures were increased using a pressure membrane at the beamline, and time was taken after a pressure increase for ruby fluorescence (pressure) to become invariant between subsequent collections.

The 2D Debye–Scherrer diffraction rings were integrated to 1D diffraction patterns using *Dioptas*, and these patterns were imported into GSAS-II^23^ for phase indexing and lattice parameter refinements. Pawley refinements were performed on the α, β, and γ phases to determine space group symmetry and obtain lattice parameters for equation of state fitting. Refinements were performed three times for each pressure, with the average lattice parameters and volumes reported in Tables S1–3. Errors were calculated from the largest difference between the refinement values and the average. Rietveld refinement was unreliable in the α phase due to data quality issues in a DAC (see Supplementary Discussion 2 for details). Using EOSFit,^58^ the second-order Birch–Murnaghan equation of state below in eq 4 was used to fit volume variation with pressure and determine initial volume and bulk modulus values for each phase

4 $$P\left(V\right)=\frac{3}{2}K_{0}\left[{\left(\frac{V_{0}}{V}\right)}^{7/3}-{\left(\frac{V_{0}}{V}\right)}^{5/3}\right]$$

where *P­(V)* is pressure as a function of volume, *V* is volume, *V*_0_ is initial volume, and *K*_0_ is the bulk modulus at ambient pressure. The use of a second-order fit was verified through construction of an *F-f* plot, which exhibited a horizontal slope for each phase.^24^

### Variable-Temperature, Ambient-Pressure PXRD Measurements and Analysis

X-ray total scattering measurements were performed at beamline 28-ID-1 (λ = 0.1665 Å) of the National Synchrotron Light Source II at Brookhaven National Laboratory. Powder samples were loaded into Kapton capillaries with an outer diameter of 1 mm, which were then loaded into a liquid He cryostat to control the temperature. Data were collected from 4 – 449 K upon heating. Diffraction data were collected using a PerkinElmer area detector with sample-to-detector distance of 0.8712 m. The 2D diffraction images were corrected for the polarization, detector transmission, and flat field. Badly performing pixels were masked prior to integration to 1D patterns using pyFAI.^59^ The X-ray wavelength and detector distance were calibrated using a nickel diffraction standard in pyFAI.

The 2D Debye–Scherrer diffraction rings were integrated to 1D diffraction patterns using pyFAI, and these patterns were imported into TOPAS-7 for Rietveld refinement.^60^ Single-crystal structures collected at 100 K (*I*4_1_ *cd*), 300 K (*I*4_1_ *cd*), and 400 K (*Ia*3̅*d*) were used for the refinements from 4 to 149 K, 155 to 359 K, and 369 to 449 K, respectively. Thermal parameters were constrained to the CIF values. Without constraints, thermal parameters for Cs sites widely diverged, likely due to large thermal displacement within the A-site cavity, and thermal parameters for Cl became negative, likely due to its weaker diffraction than the heavy Cs and Au atoms present in the sample.

### High-Pressure Raman Measurements

High-pressure Raman Spectroscopy measurements at 300 K were performed using a Horiba XploRA+ Confocal Raman spectrometer with a MicroRaman Optical microscope in the Stanford Nano Shared Facilities. A 10× magnification long-working distance objective was used. Raman spectroscopy measurements were performed using 638 and 785 nm excitation lines. Ruby fluorescence was used to measure pressure^57^ stimulated by a 532 nm excitation line. The laser power was estimated to be less than 1 mW (laser spot size ∼ 5 μm). Error in measured pressures is ca. 5% due to random error in shock wave experiments used in ruby fluorescence calibration.^57^ See the section detailing high pressure optical absorption measurements for the cryostat and optical table setup used for Raman collection at 80 K, which was collected using a 532 nm laser line excitation with <1 mW of power to avoid sample degradation.

### High-Pressure Resistivity Measurements and Analysis (Adapted from Reference 61)

#### Measurement and Magnetoresistance and Hall Effect Modeling

Low-temperature magnetotransport measurements were performed down to 2 K and in magnetic fields up to 9 T in a Quantum Design PPMS using a ^4^He cryostat. Measurements collected at 300 K and zero-field were performed using galvanostatic electrochemical impedance spectroscopy (GEIS) with a Bio-Logic SP 200 potentiostat/galvanostat. Van der Pauw (four-point) resistances were measured with AC resistance bridges with a voltage limit of 95 mV to avoid sample degradation. They were subsequently converted to longitudinal resistivity (*ρ*_*xx*_) and Hall resistivity (*ρ*_*yx*_) based on the sample thickness at each pressure. The sample chamber was checked at each pressure point to ensure that proper van der Pauv configuration of the leads was maintained. The method for longitudinal resistivity is described in detail in ref 61 and adapted here.

Briefly, for longitudinal resistivity using the van der Pauw method, voltage was measured across two adjacent leads while current was passed between the other two. Two resistance values are required to calculate the sheet resistance (*R*_s_): a horizontal resistance *R*_h_ found by measuring voltage across the first and second leads, for example, then a vertical resistance *R*_v_ from the voltage across the second and third leads. Here, *R*_s_ (Ω) is given by the following van der Pauw eq (eq 5):

5 $$e^{-\pi R_{h}/R_{s}}+e^{-\pi R_{v}/R_{s}}=1$$

Alternatively, the van der Pauw equation can be solved for *R*_s_ as follows in eq 6:

6 $$R_{s}=\frac{\pi }{\ln\left(2\right)}\cdot \frac{R_{v}+R_{h}}{2}\cdot f\left(R_{h},R_{v}\right)$$

In this equation, *f*(*R*_h_, *R*_v_) is the tabulated^62–64^ van der Pauw geometric factor that depends on the ratio of *R*_h_ and *R*_v_. From *R*_s_ (Ω), the longitudinal resistivity, ρ (Ω·cm), is calculated by multiplying *R*_s_ by the thickness, *t* (cm). The initial sample chamber thickness value can be obtained by an interference measurement on a closed DAC immediately before sample loading by using the following set of eqs (eqs 7–9)­

7 $$2nd=k\lambda_{0}$$

8 $$2nd=\left(k-i\right)\lambda_{i}$$

9 $$\frac{k}{\left(k-i\right)}=\frac{\lambda_{i}}{\lambda_{0}}$$

where *n* is the refractive index (*n* = 1 for air), *d* is the thickness between diamond culets, *k* is the interference order number, and λ_0_ and λ_i_ are the wavelengths (λ_0_ < λ_i_) at interference maxima with *k* differing by an integer value of *i* (*i* = 1 for adjacent maxima). First, *k* is solved, allowing a solution for *d*. Sample thickness of 37.44 μm was measured at room temperature and estimated upon compression from a separate experiment where interference was measured through a transparent potassium bromide sample at different pressures.^65^ The estimated sample thickness at the highest pressure was used as the thickness for all decompression measurements.

For Hall resistivity, voltage was measured across two diagonal leads while current was passed between the other two diagonal leads to measure the Hall resistance. Hall resistance was converted to Hall resistivity based on the sample thickness at each pressure as described previously.

The field-dependent longitudinal and Hall resistivities were measured using the van der Pauw configuration described above and were subsequently symmetrized and antisymmetrized, respectively, prior to further analysis. A two-band model (eqs 2a and 3a) was employed to fit the field-dependent resistivities *ρ*_*xx*_(*B*) and *ρ*_*yx*_(*B*):

2a $$\rho_{yx}\left(B\right)=\frac{B}{e}\frac{\left(n_{h}{\mu_{h}}^{2}-n_{e}{\mu_{e}}^{2}\right)+\left(n_{h}-n_{e}\right){\left(\mu_{e}\mu_{h}B\right)}^{2}}{{\left(n_{e}\mu_{e}+n_{h}\mu_{h}\right)}^{2}+{\left(n_{h}-n_{e}\right)}^{2}{\left(\mu_{e}\mu_{h}B\right)}^{2}}$$

3a $$\rho_{xx}\left(B\right)=\frac{1}{e}\frac{\left(n_{e}\mu_{e}+n_{h}\mu_{h}\right)+\left({n_{h}\mu }_{e}+{n_{e}\mu }_{h}\right)\mu_{e}\mu_{h}B^{2}}{{\left(n_{e}\mu_{e}+n_{h}\mu_{h}\right)}^{2}+{\left(n_{h}-n_{e}\right)}^{2}{\left(\mu_{e}\mu_{h}B\right)}^{2}}$$

Here, *B* is the magnetic field, *e* is elementary charge, *n*_e_ and *n*_h_ are the carrier densities of electrons and holes, respectively, and *μ*_e_ and *μ*_h_ are their corresponding mobilities. These two equations were fit to the Hall resistivity and magnetoresistivity in a simultaneous global fitting for each temperature at each pressure.

#### Resistivity Analysis to Determine Activation Energies of Conductivity

Variable-temperature conductivity measurements with no applied magnetic field exhibited complex behavior, likely from the presence of different barriers at different pressures and temperatures (Figures S29–30). Attempts to fit conductivity behavior for all temperatures to various transport models necessitated multiple transport expressions for a significant fit. Variable-temperature conductivity measurements up to 8.9(1) GPa were best fit with two hopping expressions (likely for electrons and holes). Variable temperature conductivity in the γ phase (fit at 18, 22, and 53 GPa) necessitated three Arrhenius expressions for a significant fit of the entire temperature range, which produced unreasonable activation energy values. As a result, we only consider a single Arrhenius fit in the linear region of each variable temperature conductivity sweep (250 to 300 K) in the γ phase to determine *E*_a_ near room temperature. eqs 10 and 11 below describe one expression for each pathway, where *A* is the preexponential factor, *E*_a_ is the activation energy, *k*_*B*_ is Boltzmann’s constant, and *T* is temperature in Kelvin.

10 $$Arrheniu\mathrm{s:}\sigma =Ae^{\left(-E_{a}/k_{B}T\right)}$$

11 $$Hoppin\mathrm{g:}\sigma =A\frac{1}{T}e^{\left(-E_{a}/k_{B}T\right)}$$

The transition from hopping to an Arrhenius mechanism suggests a transition from localized, outer-sphere IVCT to complex band-like transport, which is consistent with our electronic structure calculations.

### High-Pressure Optical Absorption and Reflectivity

Optical absorption and reflectivity measurements were performed at Beamline 22-IR-1 of the National Synchrotron Light Source II (NSLS-II) at Brookhaven National Laboratory (BNL). Far-infrared (65 cm^–1^ to 650 cm^–1^) measurements used a Bruker Vertex 80v FT-IR spectrometer coupled to a custom IR microscope (WD = 40 mm, NA = 0.5) with a Bolometer detector. Both mid-infrared (MIR) (500 cm^–1^ to 8000 cm^–1^) and near-infrared (NIR) (2,500 cm^–1^ to 11,000 cm^–1^) absorbance measurements were collected using a Bruker Vertex 80 FT-IR spectrometer coupled to a Hyperion-2000 IR microscope with a KBr or CaF_2_ beamsplitter and a MCT detector. Interference fringes caused by multiple reflections from diamonds and the sample were removed from the spectra using a Fourier transform analysis in Datlab.^66^ Measurements at 80 K were conducted on the same optical setups using a compact cryostat made by Cryoindustries of America, Inc.

### Absorption Onset Determination

Bandgaps were not fit using Tauc plots due to the localized nature of intervalence charge transfer. Instead, each absorption data onset (with interference fringes removed) was linearly fit and the *x*-intercept was extrapolated to determine the optical gap for each pressure. Only spectra in the near- and mid-IR that had substantial portions of the onset visible in the measured data were fit.

## Supplementary Material



## Data Availability

The data that support the findings of this study are openly available in the Stanford Digital Repository at https://purl.stanford.edu/pz597qj6459.

## Notes

Supplementary Methods, Supplementary Figures 1–48, Supplementary Tables 1–9, Supplementary Discussions 1–6, and Supplementary References (PDF)
