# Supplementary material for: Pressure-Induced Metal-like Transport and Magnetoresistance in a Au2+–Au3+ Halide Perovskite
Source: ACS Cent Sci. 2026 Jun 24;12(8):1132–45. doi: 10.1021/acscentsci.6c00315 (PMC13523498; doi:10.1021/acscentsci.6c00315)
Supplement: Supplementary file 1 [file oc6c00315_si_001.pdf]

Supplementary Information

Pressure-induced metal-like transport and magnetoresistance in a Au^{2+}–Au^{3+} halide perovskite

Christina R. Deschene,[†] Armin Eghdami,[‡] Yijun Yu,[§,¶] Alex Smith,[‡] Zhenxian Liu,[‖] Frederick P. Marlton,[†,χ] Wendy L. Mao,[§,¥] Harold Y. Hwang,[§,¶] Jeffrey B. Neaton,[‡,Š,€] and Hemamala I. Karunadasa[†,§,*]

[†] Department of Chemistry, Stanford University, Stanford, California 94305, United States
[‡] Department of Physics, University of California Berkeley, Berkeley, California 94720, United States
[§] Stanford Institute for Materials and Energy Sciences, SLAC National Laboratory, Menlo Park, California 94025, United States
[¶] Department of Applied Physics, Stanford University, Stanford, California 94305, United States
[‖] Department of Physics, University of Illinois at Chicago, Chicago, Illinois 60607, United States
[χ] Centre for Clean Energy Technology, School of Mathematical and Physical Sciences, Faculty of Science, University of Technology Sydney, Sydney, NSW, 2007, Australia
[¥] Department of Earth and Planetary Sciences, Stanford University, Stanford, California 94305, United States
[Š] Materials Sciences Division, Lawrence Berkeley National Laboratory, Berkeley, California 94720, United States
[€] Kavli Energy NanoSciences Institute at Berkeley, Berkeley, California 94720, United States
*e-mail: hemamala@stanford.edu

Table of Contents:

Supplementary Methods 2
Supplementary Figures 5
Supplementary Tables.. 50
Supplementary Discussion.. 56
Supplementary References.. 62

Supplementary Methods

Methods for Diamond-Anvil Cell (DAC) Loading

DACs were prepared by pre-indenting metal gaskets to ca. 25 GPa, 20 GPa, and 18 GPa for measurements with 300 µm, 400 µm, and 500 µm culets, respectively, to achieve a thickness at the indentation of ~25-40 µm. A sample chamber was drilled in the center of the indentation by electrical discharge machining (Hylozoic Products), and the sample was loaded in the chamber with a ruby sphere (Betsa, 3600 ppm Cr^{3+}) to be used as a pressure calibrant.[1] All optical measurements were performed in axial geometry where the diamonds served as optical windows.

Powder X-ray Diffraction (PXRD)

DACs with 300 µm and 400 µm culets were prepared for PXRD measurements to allow for measurements across a large pressure range (300 µm) and measurements with finer pressure steps at phase changes (400 µm). Tungsten gaskets were pre-indented and drilled with a 120 µm (165 µm) drill bit for 300 µm (400 µm) culet indentation. Sample powders were centrally loaded to take up half of the sample chamber, and a ruby sphere was placed in the space between the sample and gasket. The DACs were loaded with neon as a pressure-transmitting medium at the Advanced Light Source (ALS) at Lawrence Berkeley National Laboratory (LBNL) or GSECARS/COMPRES Gas Loading System at the Advanced Photon Source at Argonne National Laboratory.

Raman Spectroscopy

The same DAC preparation procedure for PXRD was used for Raman measurements at 300 K. Only a DAC with 300 µm culets was used. The DAC was loaded with neon as a pressure-transmitting medium using the GSECARS/COMPRES Gas Loading System at the Advanced Photon Source at Argonne National Laboratory. Measurements at 80 K were prepared with the same gasket and culet specifications but were loaded with liquid argon as a pressure-transmitting medium.

Absorbance/Reflectivity Measurements

DACs with 300 µm and 500 µm culets were prepared for absorbance and reflectivity measurements to allow for data collections across a large pressure range in the visible, mid-, and near-infrared (300 µm) regions and measurements with adequate signal-to-noise ratios in the far-infrared (500 µm) region. Absorption measurements at 80 K were prepared with the same gasket and culet specifications but were loaded with liquid argon as a pressure-transmitting medium.

Visible/Near-Infrared/Mid-Infrared (Vis-NIR-MIR) Measurements

DACs with 300 µm culets were used to pre-indent tungsten gaskets. Sample chambers were drilled using a 130 µm drill bit. Sample powders were lightly compressed between exposed diamonds to make a thin (~10 µm) pellet that was optically translucent. The sample chambers were packed with KCl as a pressure-transmitting medium. The KCl powder was packed until it became optically transparent, after which a thin layer was carved out of half of the sample chamber. A portion of the thin sample pellet was used to fill this space, and a ruby ball was placed on the KCl on the opposite side of the sample chamber (Figure S9C inset). In this loading, the sample chamber half, which was only filled with KCl, served as an absorbance background.

Far-Infrared (FIR) Measurements

DACs with 500 µm culets were used to pre-indent tungsten gaskets. Sample chambers were drilled using a 205 µm drill bit. Sample powders were lightly compressed between exposed diamonds to make a thin (~10 µm) pellet that was optically translucent. A portion of this pellet near the size of the sample chamber was added to the chamber, and a ruby ball was attached to the edge of the gasket just inside the sample chamber. Petroleum jelly was added as a pressure transmitting medium and moved with a needle to guide the ruby further inside the sample chamber. We note that measurements conducted with 300 µm culets and a 120

μm drill bit appeared to exhibit no absorption in the far-infrared at intermediate pressures due to a low signal-to-noise ratio of the smaller sample chamber, and the measurements with 500 μm culets and the larger sample chamber were required to resolve absorption features at pressures where the absorption onset moves to low energies in the far-infrared.

<u>Magnetoresistance/Hall-Effect Measurements</u>

DACs with 300 μm culets were used to pre-indent rhenium gaskets (thickness ~250 μm) to a thickness of *ca.* 25 μm. Sample chambers were drilled using a 205 μm drill bit. Gaskets were insulated through compression (*ca.* 25 GPa) of cubic boron nitride (c-BN) epoxy[2] into the sample chamber such that it covered the sample chamber and gasket-diamond interfaces up to the top of the gasket indent. Gasket surfaces were insulated with a thin layer of epoxy. Smaller sample chambers (*ca.* 100 μm in diameter) were carved out of the c-BN epoxy on the piston diamond. Initial sample chamber thickness values were determined through interference measurements on a closed DAC immediately before sample loading following equations 1 - 3:

$$2nd = k\lambda_1 \tag{1}$$
$$2nd = (k-i)\lambda_i \tag{2}$$
$$\frac{k}{(k-i)} = \frac{\lambda_i}{\lambda_1} \tag{3}$$

Here, n is the refractive index (n = 1) for air, d is the thickness between diamond culets (in nm), k is the interference order number, and λ_1 and λ_i are the wavelengths ($\lambda_1 < \lambda_i$) at interference maxima with k differing by an integer value of i (i = 1 for adjacent maxima). A solution for d is obtained through first solving for k. A sample chamber thickness of 37 μm was measured for this experiment.

A powder sample of $Cs_4Au^{II}Au^{III}_2Cl_{12}$ was loaded into the sample chamber carved out of the c-BN epoxy, and a ruby sphere was placed centrally on the sample. Four Pt electrical leads were overlaid on the sample in a van der Pauw configuration (Figure S28A-B). No pressure-transmitting medium was used to ensure good contact between the sample and the leads. The Pt leads were connected to external Cu wires using Ag paste. The Cu wires were soldered onto a Physical Property Measurement System puck. The puck was insulated from the DAC through placing several layers of glass coated in Kapton tape between them (Figure S28C).

DFT calculations

We performed density functional theory (DFT) calculations using the HSE range-separated hybrid functional[3] as implemented in VASP,[4] with $\alpha = 0.25$ and range-separation parameter $\omega = 0.2$. Spin-polarized calculations were performed in all cases. A plane-wave basis set with an energy cut-off of 500 eV was used. Brillouin zone sampling employed Γ-centered k-point meshes (2×2×2 unless otherwise noted). Projector-augmented wave pseudopotentials[5] included 19 valence electrons for Au, 9 for Cs, and 7 for Cl.

The initial structure was based on single-crystal XRD data with space group $I4_1cd$ (no. 110) and primitive cell vectors: $a = (-x, x, y)$, $b = (x, -x, y)$, c = $(x, x, -y)$ where x = 10.48525 Å and y = 10.64740 Å, containing 24 Au, 96 Cl, and 32 Cs atoms (Figure S11). Full structural relaxations with HSE yielded slightly expanded lattice parameters, which varied with applied pressure as follows:

0 kBar: $x \approx 10.68$ Å, $y \approx 10.85$ Å
80 kBar: $x \approx 9.69$ Å, $y \approx 9.79$ Å
160 kBar: $x \approx 9.33$ Å, $y \approx 9.39$ Å
200 kBar: $x \approx 9.21$ Å, $y \approx 9.23$ Å
240 kBar: $x \approx 9.11$ Å, $y \approx 9.11$ Å

For pressure-dependent calculations, we applied external pressure with full cell shape and volume relaxation and a convergence criteria of 1×10^{-5} eV for the electronic self-consistent loop. For the ionic relaxation, we set the convergence criteria to stop once the norms of all forces were smaller than 0.03 eV/Å.

The Wigner–Seitz radii used for magnetic moment and partial density-of-states (DoS) calculations were 1.376 Å (Au), 1.005 Å (Cl), and 1.482 Å (Cs).

We verified the robustness of the Au^{2+} site magnetization using the meta-GGA SCAN functional (in VASP: METAGGA = SCAN), which yielded a magnetization of 0.420 μ_β at 0 GPa, comparable to 0.436 μ_β from HSE, confirming the magnetization is not an artifact of the hybrid functional and further that SCAN can capture the magnetization as well.

Band-structure calculations employed single-point non-self-consistent runs along high-symmetry paths (X: 0 0 0.5, P: 0.25 0.25 0.25, N: 0 0.5 0, Γ: 0 0 0, M: 0.5 0.5 -0.5) using the zero-weight k-points method.

DoS calculations used 1000 grid points (in VASP: NEDOS = 1000) over an energy window of 3 eV with a smearing of 0.02 eV (in VASP: SIGMA = 0.02).

Effective-mass calculations used the VASP generated k-grid, supplemented by a uniform k-grid centered around band extrema of interest, auto-generated via the "Effective-Mass Calculator" code,[6] which was also used to post-process the results to obtain the effective masses. To assess the robustness of the effective-mass trends, we computed values using three different k-point step sizes: 0.015, 0.025, and 0.035 (in units of 1/Bohr). The resulting mesh combined the standard k-grid with 19 additional zero-weight points to optimize local curvature estimation.

Band-projected electron density plots were created for specific bands and k-points using the LPARD and LSEPK VASP flags.

Supplementary Figures

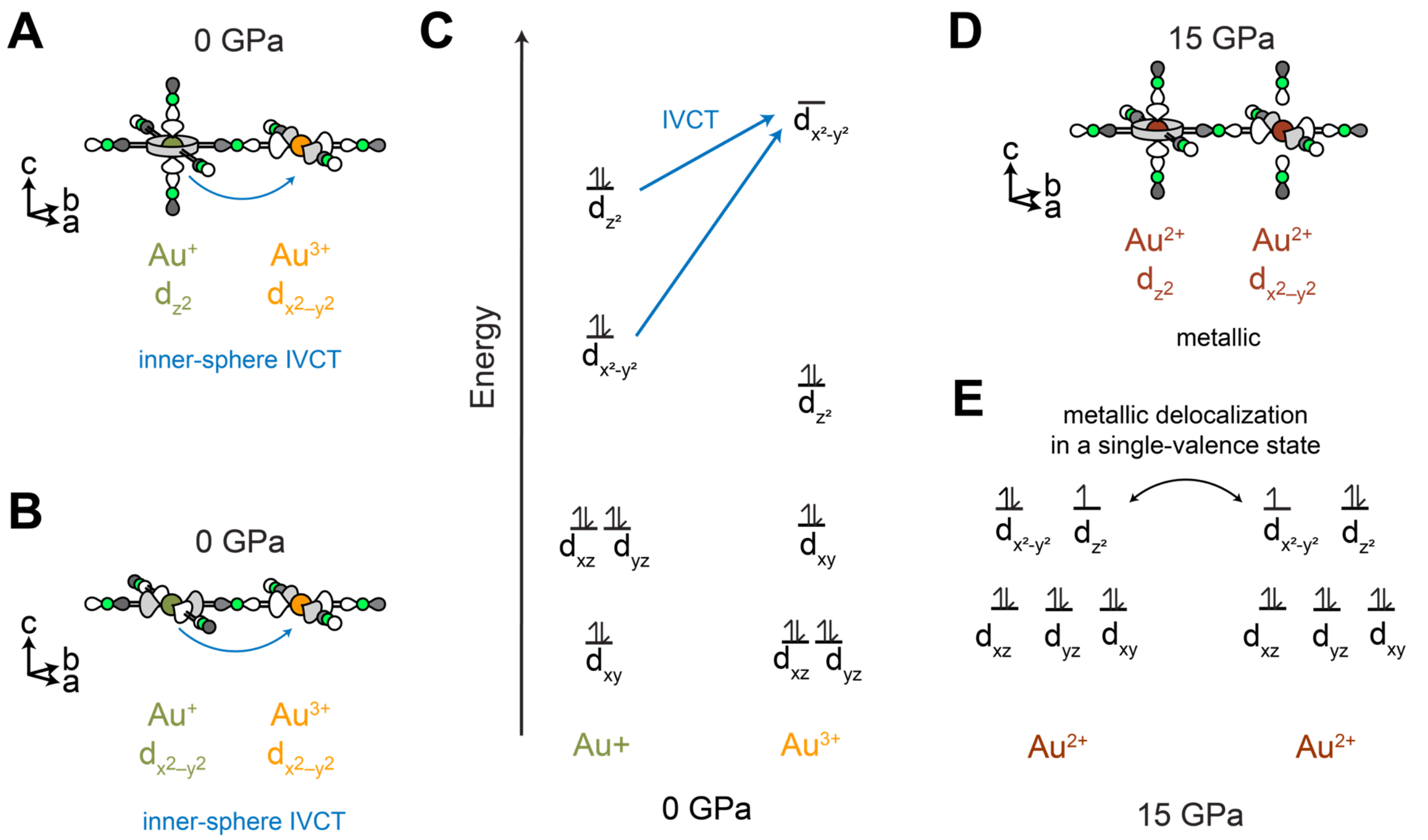

Figure S1. Schematics of possible Au $d_{x^2-y^2}$, Au d_{z^2}, and Cl p-orbital arrangements of Au^+–Cl, Au^{2+}–Cl, and Au^{3+}–Cl, subunits present in (**A-B**) $Cs_2Au^IAu^{III}Cl_6$ at 0 GPa and (**D**) "$CsAu^{II}Cl_3$" at 15 GPa. **A** and **B** are inner-sphere pathways proposed in ref. 8, determined through group theory analysis and polarized reflectivity. Gray and white orbital lobes represent positive and negative lobes, respectively. Olive, scarlet, gold, and green spheres represent Au^+, Au^{2+}, Au^{3+}, and Cl^- ions, respectively. Relative d-orbital energies of (**C**) $Cs_2Au^IAu^{III}Cl_6$ at 0 GPa (ref. 8) and (**E**) "$CsAu^{II}Cl_3$" at 15 GPa. Single-headed blue arrows represent IVCT, and double-headed black arrows represent metallic delocalization between partially filled orbitals.

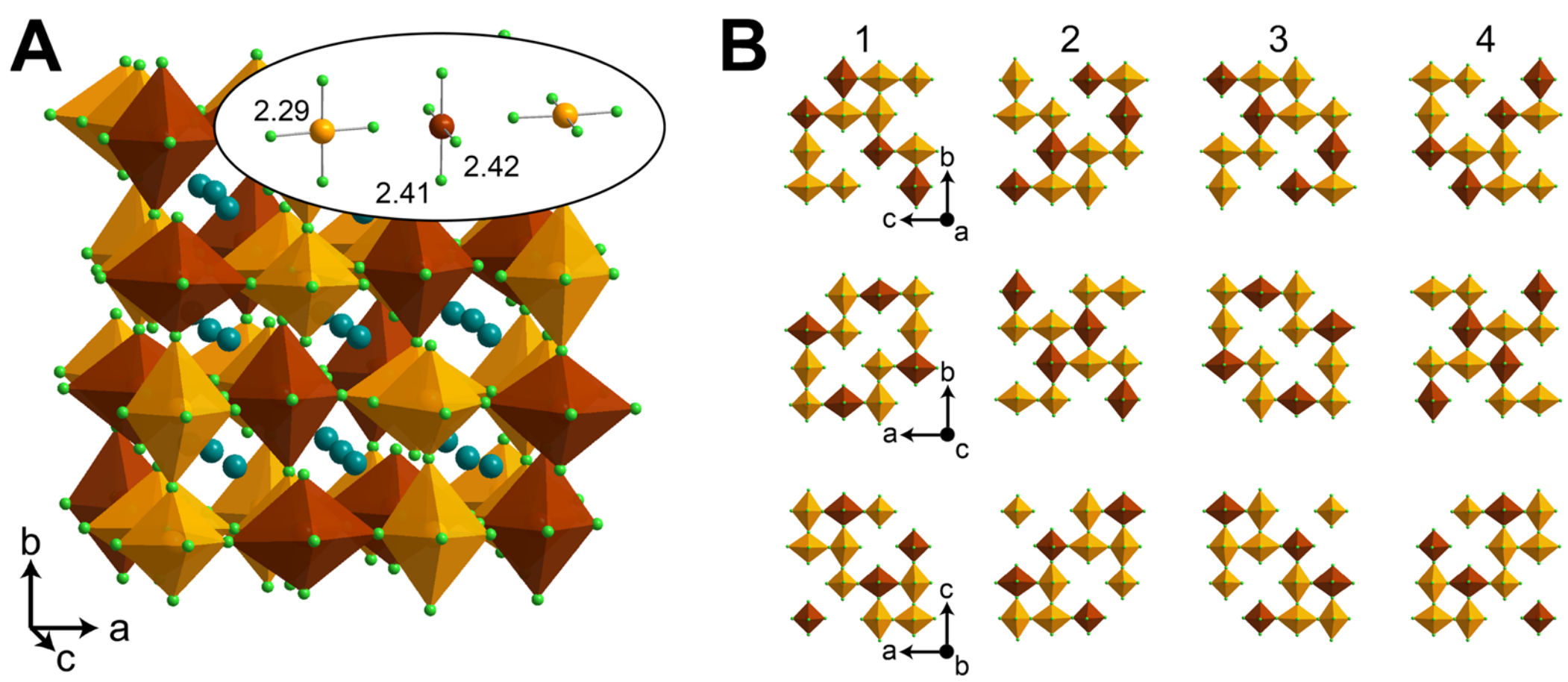

Figure S2. (**A**) Single-crystal X-ray diffraction structure of $Cs_4Au^{II}Au^{III}_2Cl_{12}$ (**1**). Teal, scarlet, gold, and green spheres represent Cs^+, Au^{2+}, Au^{3+}, and Cl^- ions, respectively. Scarlet and gold polyhedra represent $Au^{II}Cl_6$ and $Au^{III}Cl_6$, respectively. Inset: Au–Cl bond lengths (in Å) of $Au^{II}Cl_4$ and $Au^{III}Cl_4$ square-planar complexes present in the structure. (**B**) 2D representations of the single-crystal structure of **1**. Numbers represent the stacking sequence of the layers along the *a* axis (top), *c* axis (middle), and *b* axis (bottom). Representations along the *a* axis are vertical slices perpendicular to the page from the left to the right of the crystal structure in **A**, representations along the *c* axis are slices parallel to the plane of the page from the front to the back of the crystal structure in **A**, and representations along the *b* axis are slices perpendicular to the page from the bottom to the top of the crystal structure in **A**.

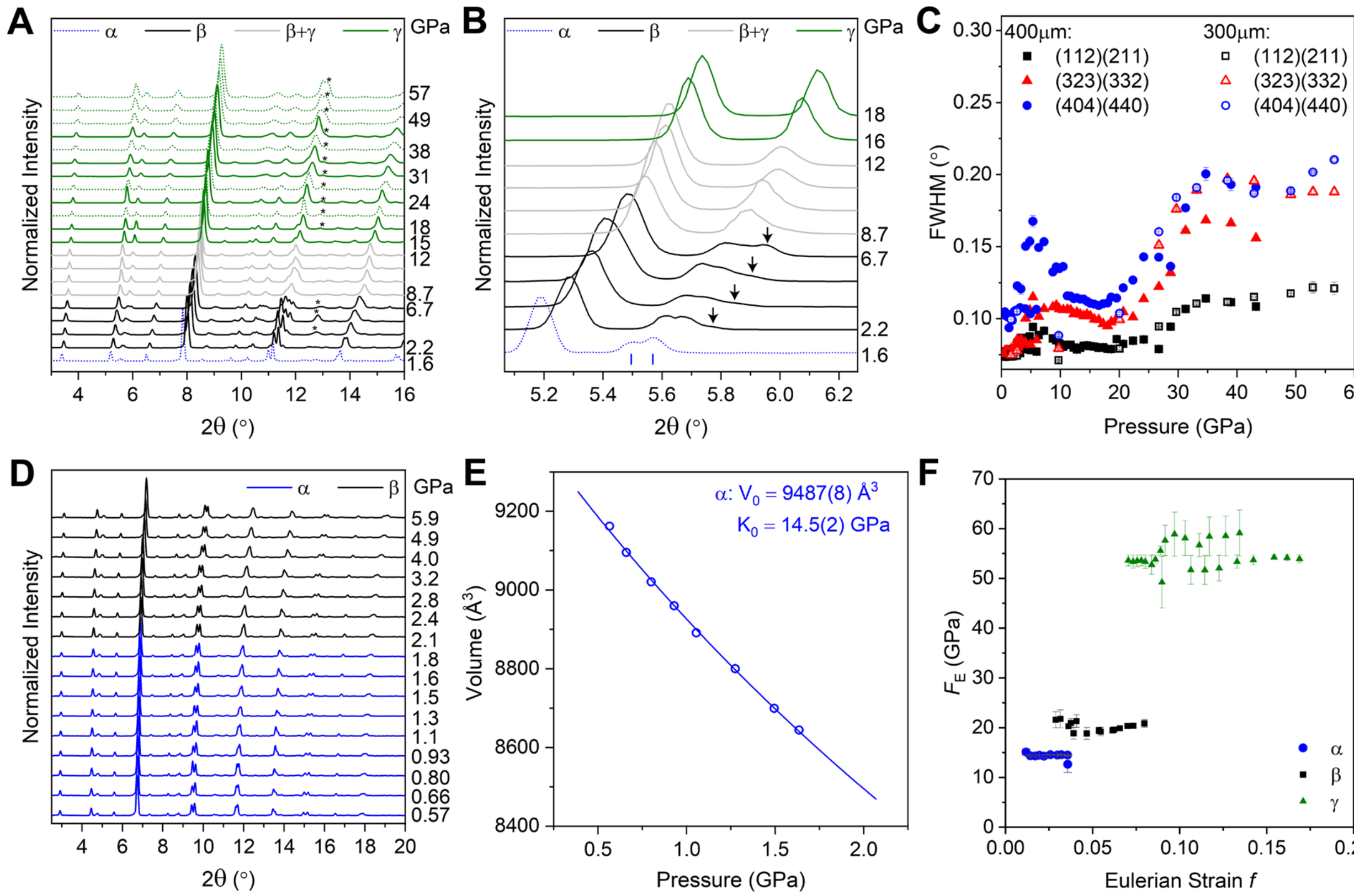

Figure S3. (**A**) High-pressure powder X-ray diffraction (PXRD; λ = 0.495 Å) spectra of **1** from 1.6 GPa to 57 GPa. Spectra plotted with solid and dashed lines were collected using 400 μm and 300 μm culets, respectively. The α (blue), β (black), and γ (green) phases correspond to *I*4$_1$*cd*, *Iba*2, and *Ia*$\overline{3}$*d* space-group assignments, respectively. Gray patterns contain both β and γ phases. Asterisks denote reflections from the tungsten gasket. (**B**) Diffraction data in **A** centered around the (004) and (400) reflection peaks (left and right blue hash marks, respectively) present in the tetragonal structure at 1.6 GPa. A low-angle shoulder appears at 2.2 GPa corresponding to the β phase (black arrows), and all three peaks merge by 15 GPa in the γ phase. (**C**) Full-width at half-maximum (FWHM) singular pseudo-Voigt peak fitting of select diffraction peaks that contain reflections from the same family. Narrowing of the FWHM with pressure between 5 and 20 GPa denotes formation of the γ phase. Increase of FWHM after 20 GPa is ascribed to worsening hydrostaticity of the sample chamber with increased pressure evidenced by the increased rate of broadening in measurements with 300 μm culets that have smaller sample chambers and contain less Ne pressure-transmitting medium than 400 μm culet loadings. (**D**) High-pressure PXRD (λ = 0.434 Å) spectra of **1** from 0.57 GPa to 5.9 GPa. Spectra were collected using 400 μm culets. The α (blue) and β (black) phases correspond to *I*4$_1$*cd* and *Iba*2 space-group assignments, respectively. (**E**) Evolution of the unit-cell volume of **1** with respect to pressure in the α phase, determined through phase indexing and Pawley refinement of diffraction patterns in **D**. A second-order Birch-Murnaghan equation-of-state fit to the *I*4$_1$*cd* phase is shown with a corresponding bulk modulus (K_0; bulk modulus at 0 GPa) and initial volume parameter (V_0). (**F**) *F*-*f* plot of the Birch-Murnaghan equation-of-state fits of the α (blue), β (black), and γ (green) phases of **1**. Horizontal slopes confirm the choice of a second order equation-of-state fit for each phase.

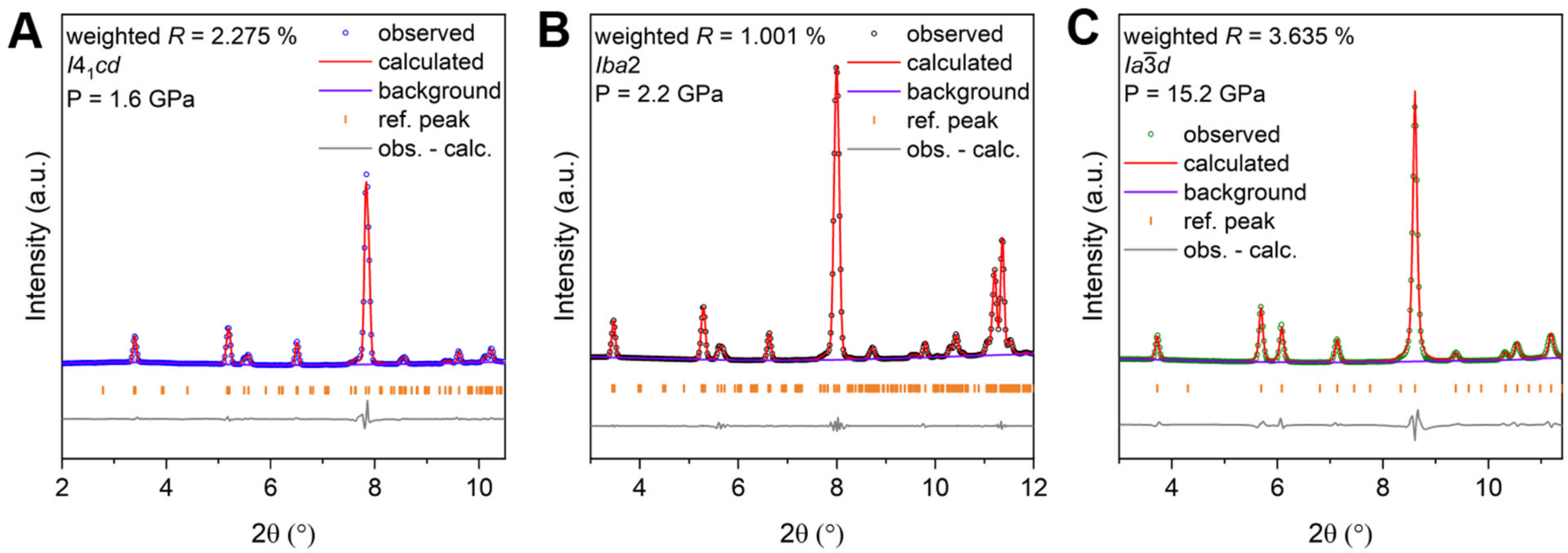

Figure S4. Indexing and Pawley refinement of powder diffraction of **1** at (**A**) 1.6 GPa, (**B**) 2.2 GPa, and (**C**) 15 GPa.

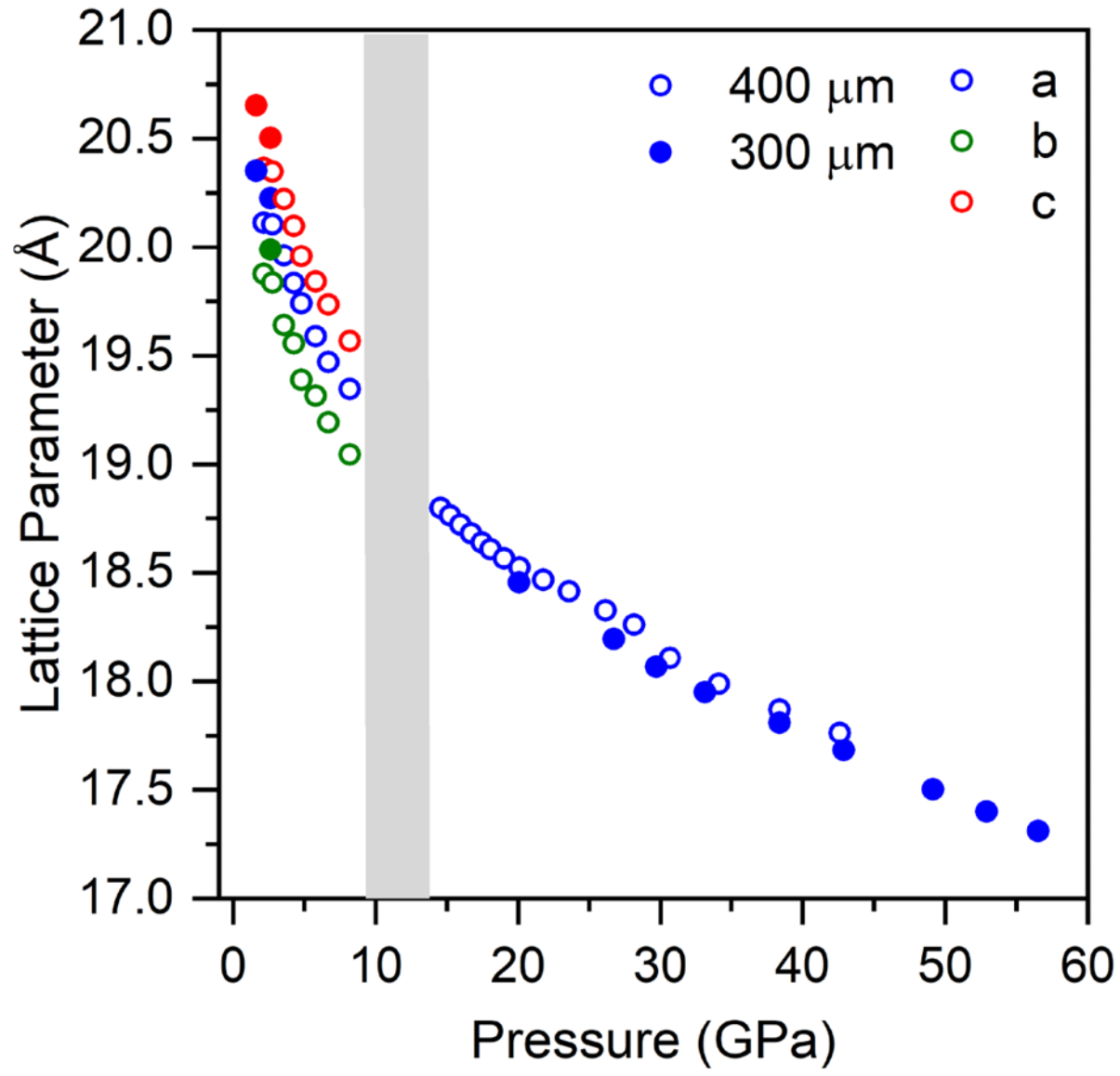

Figure S5. Lattice parameter evolution of **1** upon compression up to 57 GPa as determined through Pawley refinement. Data points plotted with hollow and solid circles were collected using 400 μm and 300 μm culets, respectively. The gray region denotes pressures where both the α and β phases were present, which prevented reliable fitting.

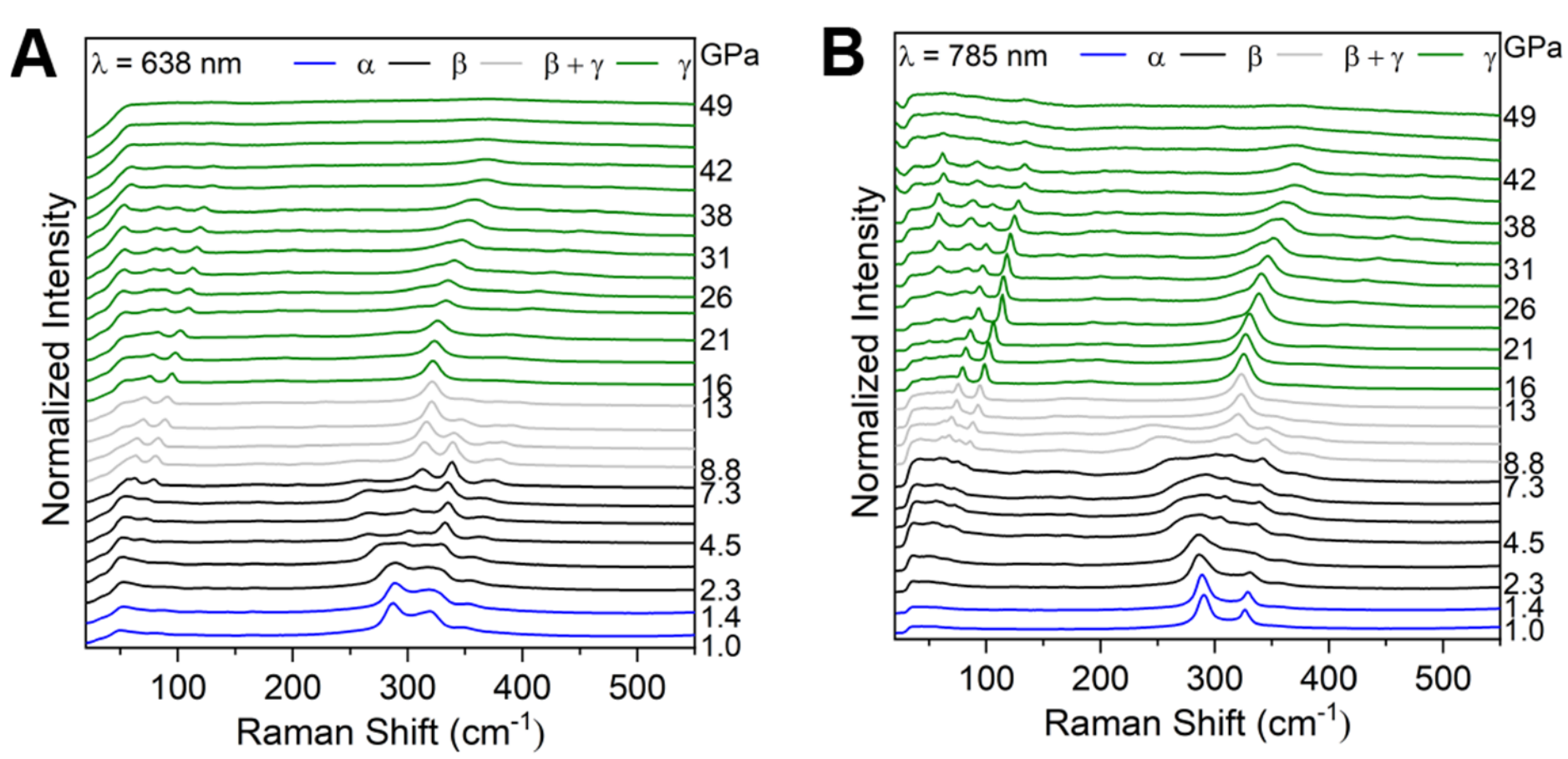

Figure S6. Raman spectra of **1** collected using (**A**) 638 nm and (**B**) 785 nm laser excitation. The α (blue), β (black), and γ (green) phases correspond to $I4_1cd$, $Iba2$, and $Ia\bar{3}d$ space-group assignments, respectively, labeled according to PXRD space-group assignments at corresponding pressures. Gray patterns contain both β and γ phases.

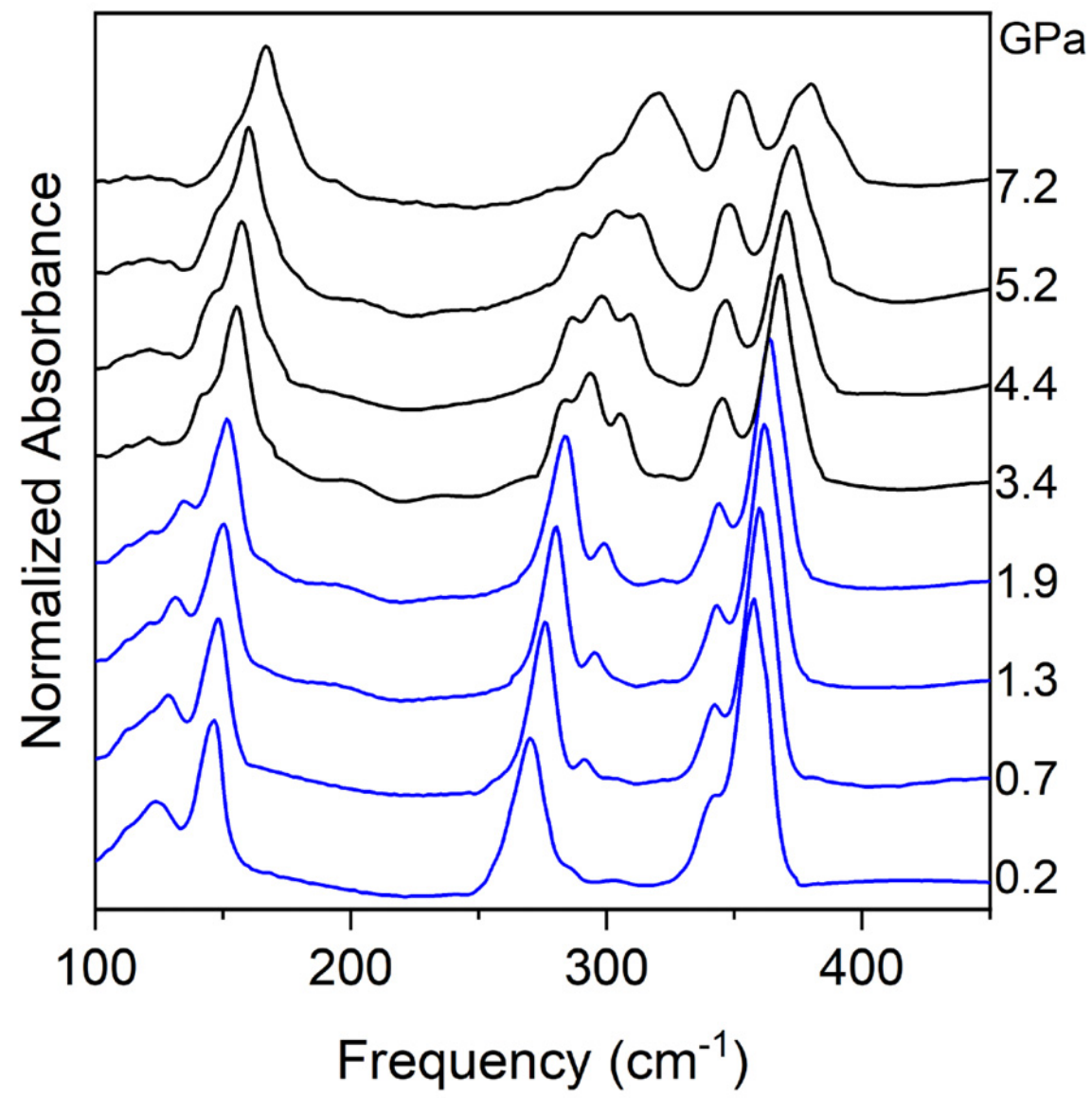

Figure S7. Far-infrared absorption spectra showing IR-active vibrational modes in the α (blue) and β (black) phases of **1**.

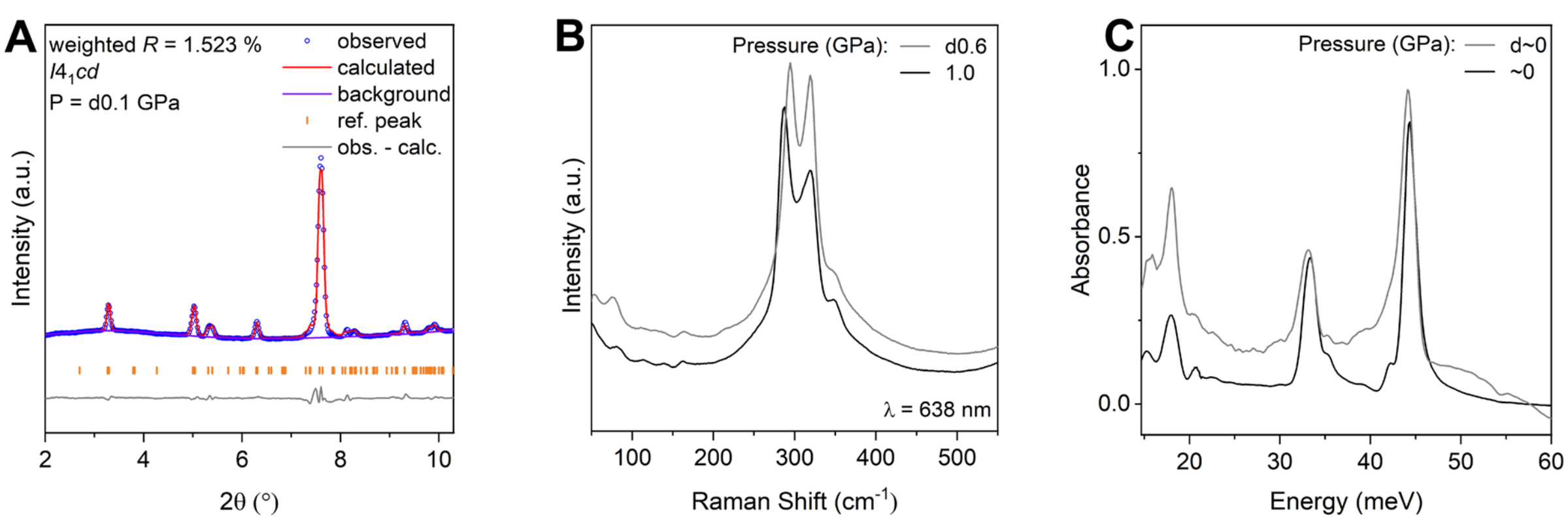

Figure S8. (**A**) Indexing and Pawley refinement of PXRD data of **1** when decompressed from 57 GPa to 0.1 GPa. (**B**) Raman (638 nm excitation) and (**C**) far-infrared absorption spectra of **1** at the first pressures measured (black) and upon decompression (gray).

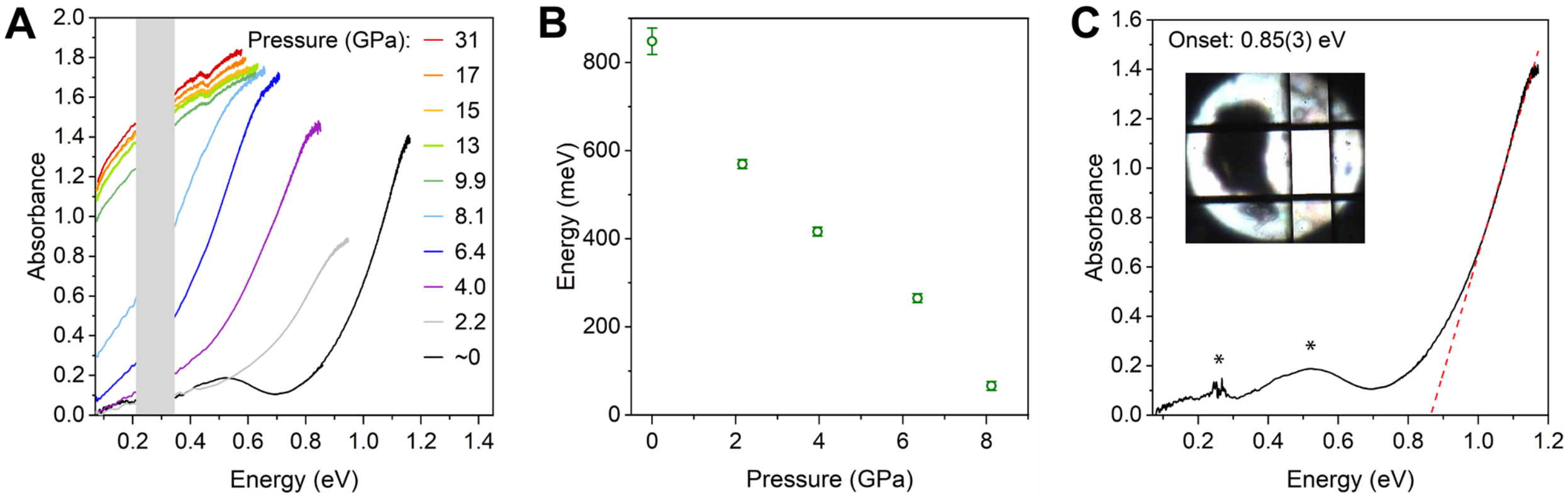

Figure S9. (**A**) Near- and mid-infrared absorbance of **1** with pressure. Gray region excludes absorption features from diamonds. (**B**) Approximate absorption onsets obtained via linear fits of the spectra in **A**. Errors are shown for the R^2 values of the linear fits. (**C**) Near and mid-infrared absorption of **1** at ambient pressure with a representative absorption onset fit to obtain values shown in **B**. Asterisks denote diamond absorption (~0.3 eV) and interference fringes (~0.5 eV). Inset shows a sample chamber image at ambient pressure.

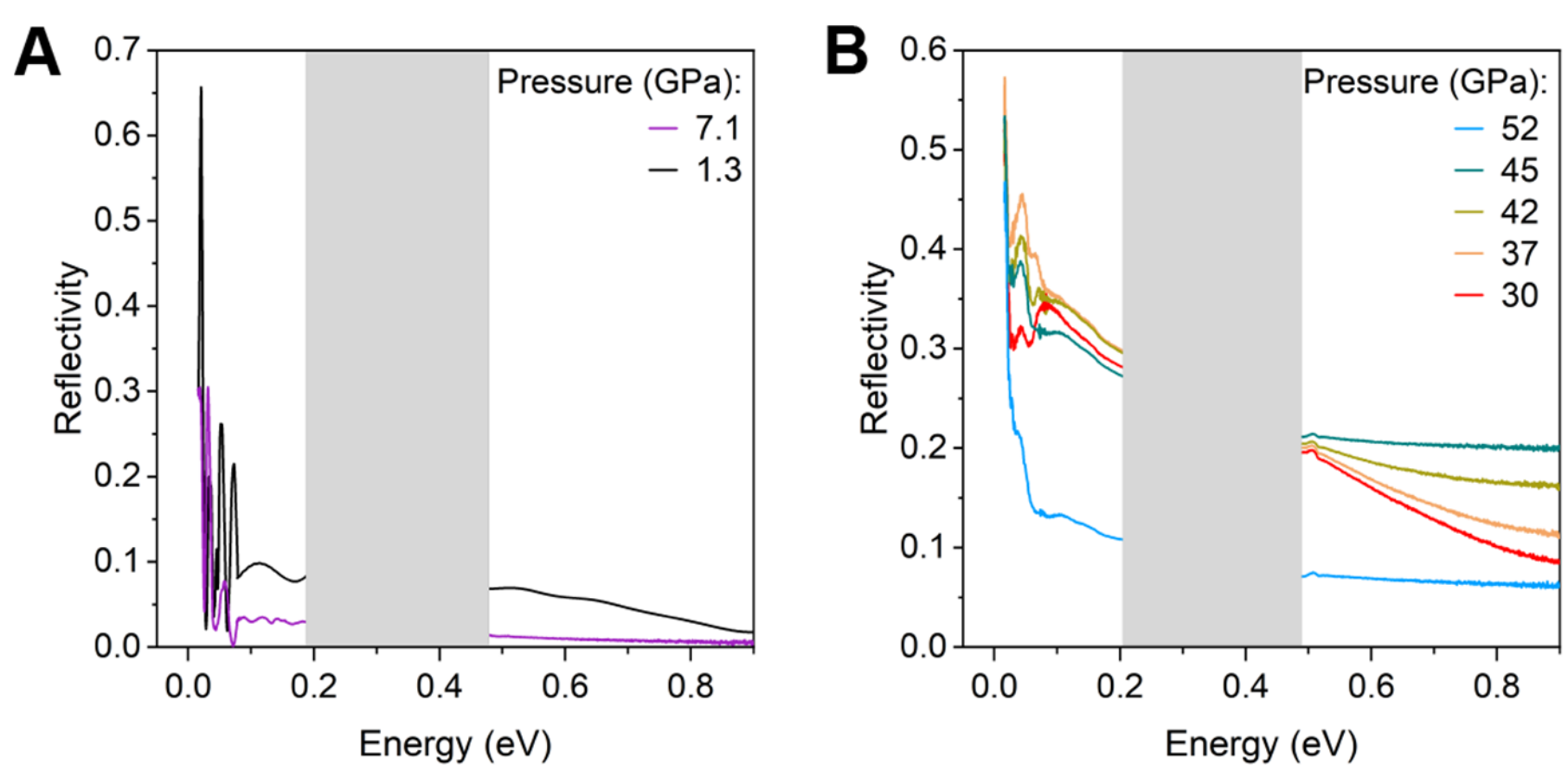

Figure S10. Reflectivity spectra of **1** at (**A**) 1.3 GPa and 7.1 GPa and (**B**) from 30 GPa to 52 GPa. Gray regions exclude absorption features from diamonds and C–H bonds in the petroleum jelly pressure-transmitting medium. Sinusoidal far-infrared reflectivity in **A** confirms the open optical gap of **1** in this pressure region.

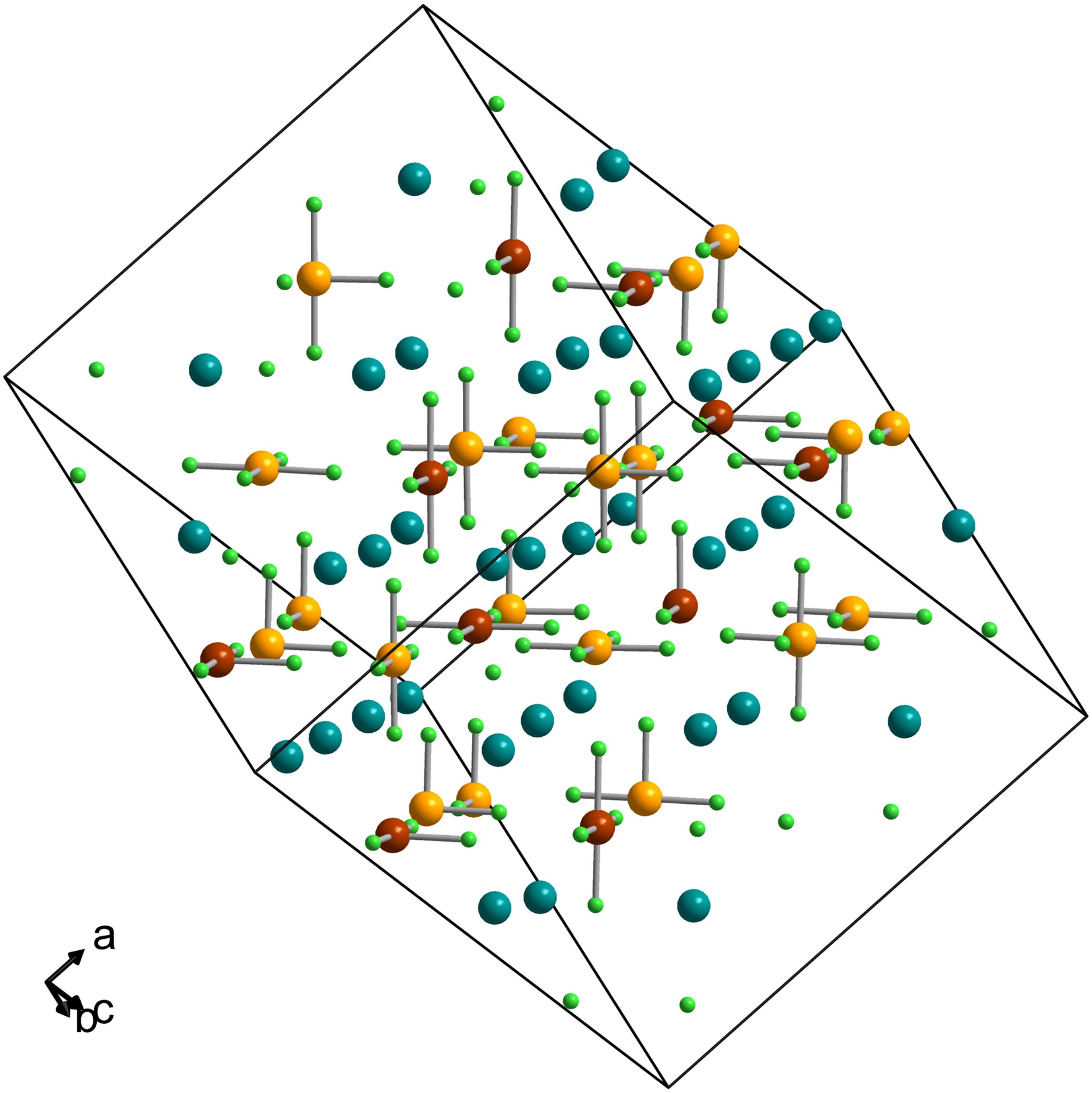

Figure S11. Reduced unit cell of **1** used for structural relaxations, band structure, and density of states calculations. Teal, scarlet, gold, and green spheres represent Cs^{+}, Au^{2+}, Au^{3+}, and Cl^{-} ions, respectively. Gray bonds are shown for the square planar complexes of $Au^{II}Cl_4$ and $Au^{III}Cl_4$. The black box represents the unit-cell edges, and the arrows denote the axes of the reduced unit cell, which differ in direction from the original unit cell from single-crystal X-ray diffraction.

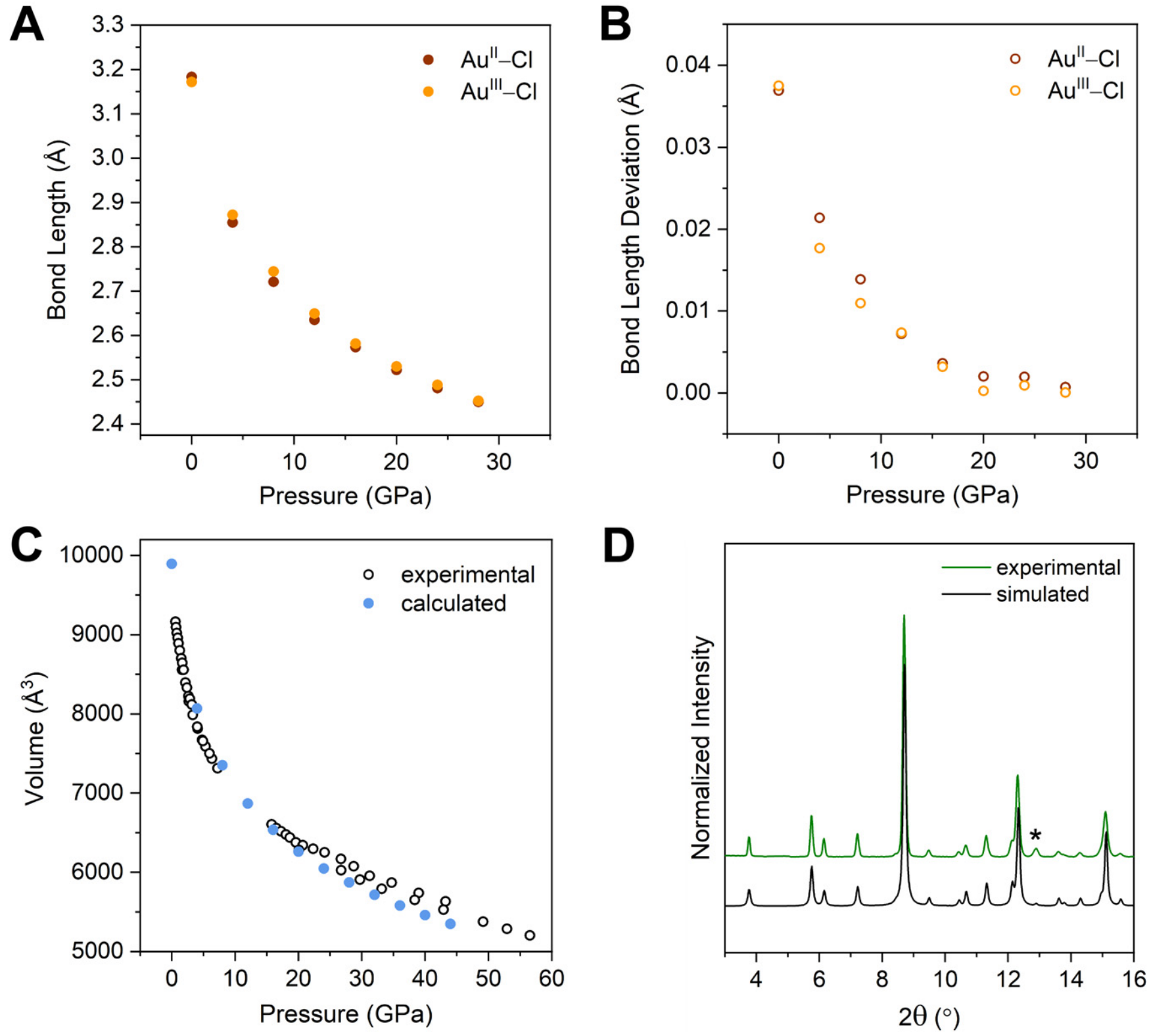

Figure S12. (**A**) Average bond length of the long Au–Cl bonds of $Au^{II}Cl_6$ (scarlet) and $Au^{III}Cl_6$ (gold) polyhedra in DFT calculations of **1** with pressure. (**B**) Deviation of the long Au–Cl bonds of $Au^{II}Cl_6$ (scarlet) and $Au^{III}Cl_6$ (gold) polyhedra compared to the average shown in **A** with pressure, which demonstrates deviation from D_{4h} symmetry. (**C**) Pressure-induced volume evolution of **1** calculated through high-pressure structural relaxation using DFT (blue) and from Pawley refinement of experimental PXRD patterns (black). (**D**) Experimental (green) and simulated (black) diffraction patterns of **1** at 20 GPa. Simulated patterns were obtained through powder pattern calculations using the relaxed crystal structure at 20 GPa. Asterisk denotes a reflection from the tungsten gasket.

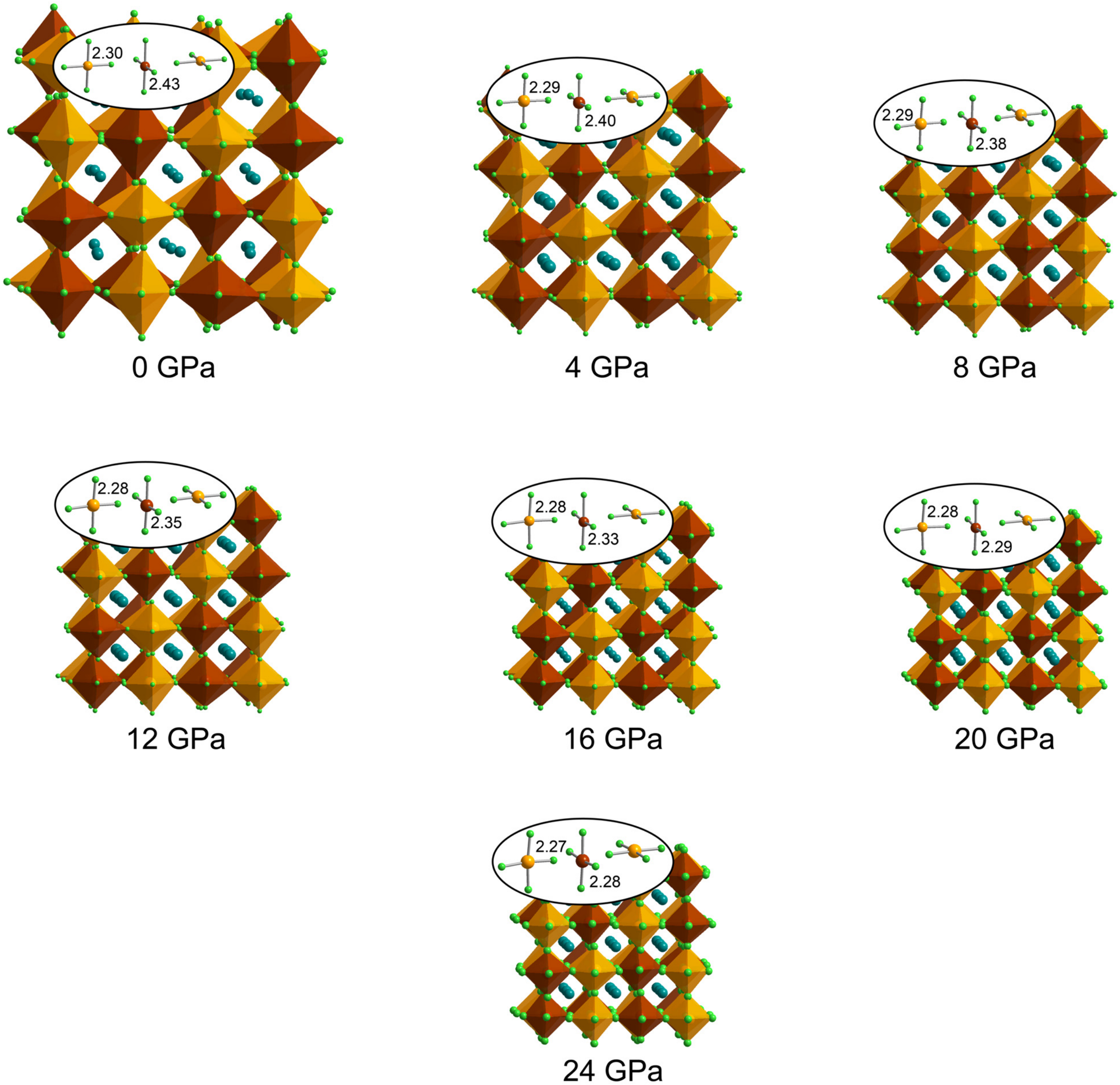

Figure S13. Single-crystal X-ray diffraction structure of **1** relaxed at 0 GPa (top left), 4 GPa (top center), 8 GPa (top right), 12 GPa (middle left), 16 GPa (middle center), 20 GPa (middle right), and 24 GPa (bottom). Teal, scarlet, gold, and green spheres represent Cs^{+}, Au^{2+}, Au^{3+}, and Cl^{-} ions, respectively. Scarlet and gold polyhedra represent $Au^{II}Cl_6$ and $Au^{III}Cl_6$, respectively. Inset: Au–Cl bond lengths (in Å) of $Au^{II}Cl_4$ and $Au^{III}Cl_4$ square-planar complexes present in the structure.

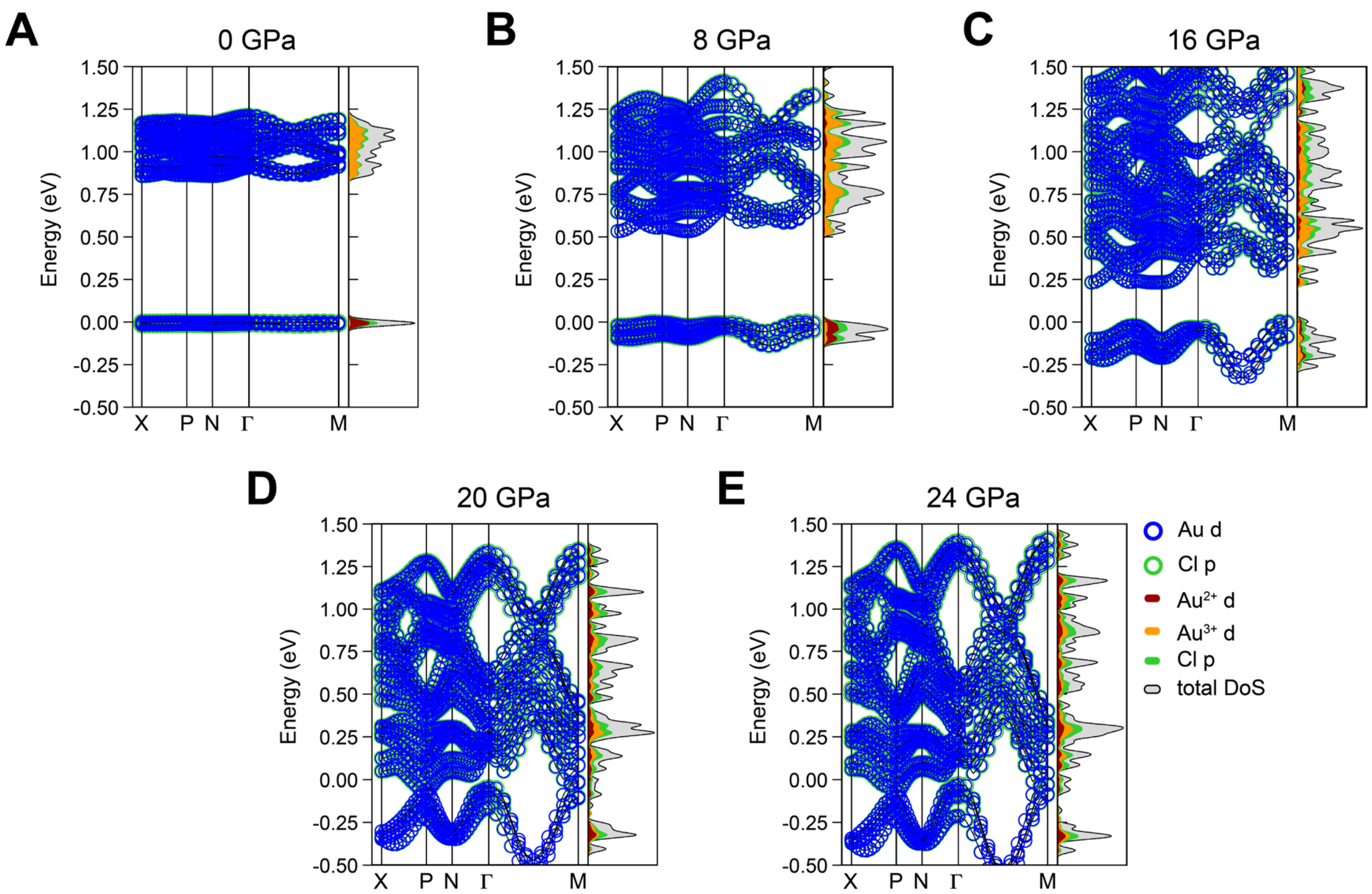

Figure S14. Band structures (left) and corresponding density-of-states (right) using DFT-HSE of the reduced unit cell of the single-crystal X-ray diffraction structure of **1** relaxed at (**A**) 0 GPa, (**B**) 8 GPa, (**C**) 16 GPa, (**D**) 20 GPa, and (**E**) 24 GPa. Blue and green circles denote Au d-orbital and Cl p-orbital contributions, respectively. Gold, scarlet, green, and gray shading in the density-of-states plots represent Au^{3+} d, Au^{2+} d, Cl p, and total contributions, respectively. Contributions from the individual Au valences were calculated for the crystallographic sites where those atoms are located in the single-crystal X-ray diffraction structure solution.

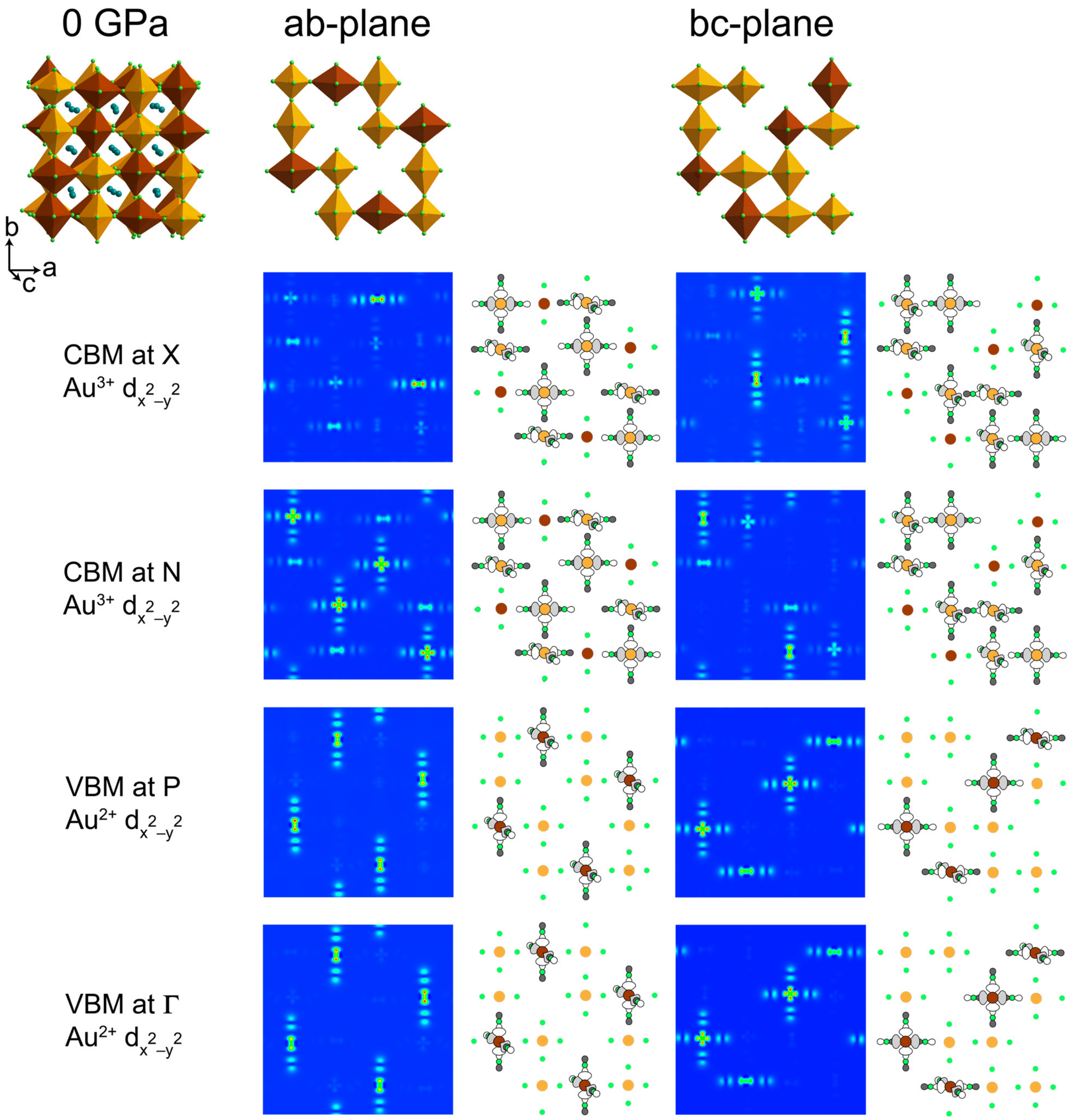

Figure S15. Single-crystal structure of **1** relaxed at 0 GPa (top left) and corresponding electron density maps with tentatively assigned orbital schematics of the VB and CB at labeled k-points along the *ab* and *bc* planes obtained from our DFT-HSE calculations. Gray and white orbital lobes represent positive and negative lobes, respectively. Scarlet, gold, and green spheres represent Au^{2+}, Au^{3+}, and Cl^{-} ions, respectively. Electron density maps are shown on a rainbow scale where red is the highest density and blue is the lowest density. Maps are scaled for the maximum and minimum density of each image individually, and cannot be compared between images.

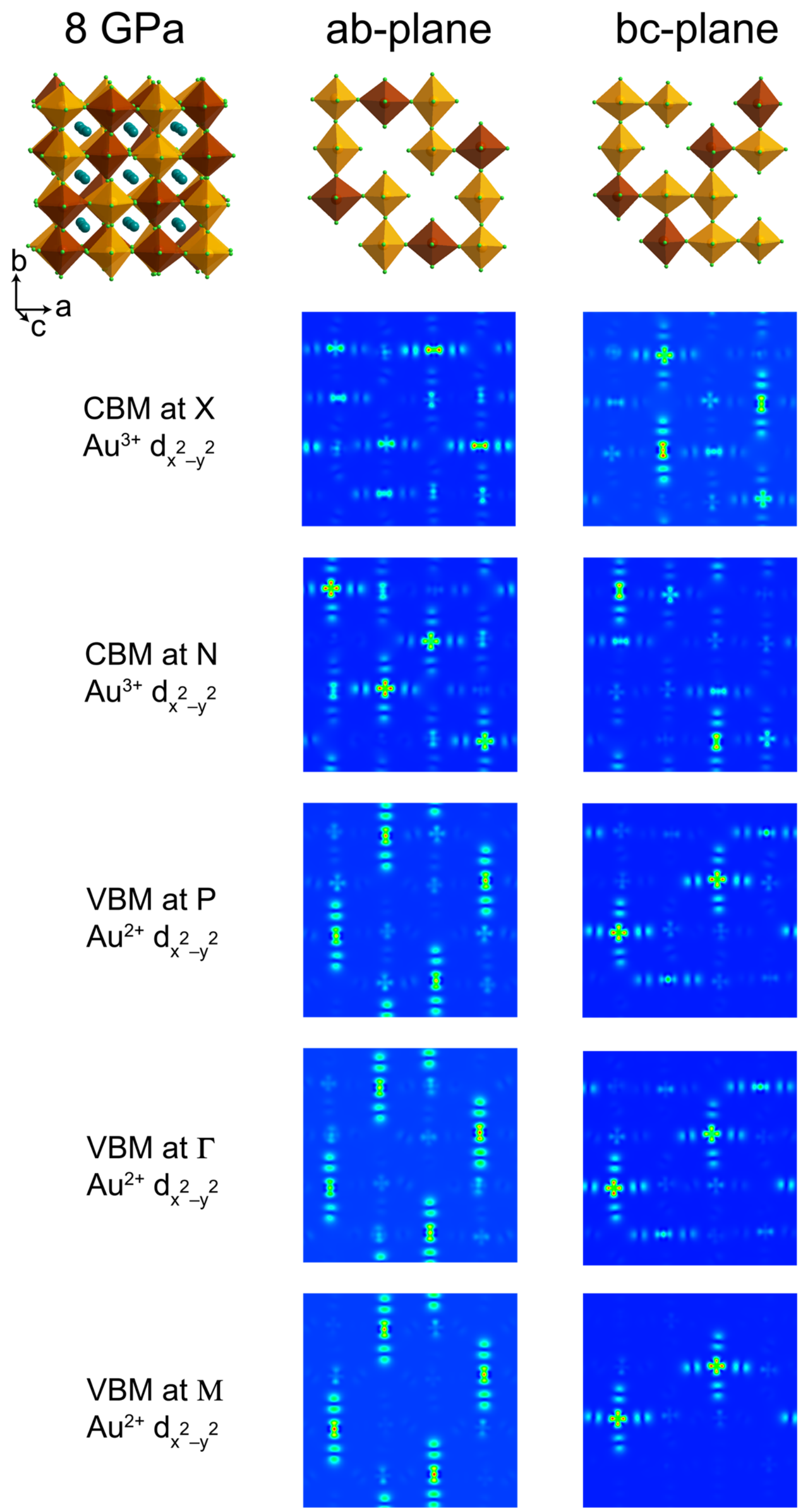

Figure S16. Single-crystal structure of **1** relaxed at 8 GPa (top left) and corresponding electron density maps of the VB and CB at labeled k-points along the *ab* and *bc* planes obtained from our DFT-HSE calculations. Teal, scarlet, gold, and green spheres represent Cs^{+}, Au^{2+}, Au^{3+}, and Cl^{-} ions, respectively. Electron density maps are shown on a rainbow scale where red is the highest density and blue is the lowest density. Maps are scaled for the maximum and minimum density of each image individually, and cannot be compared between images.

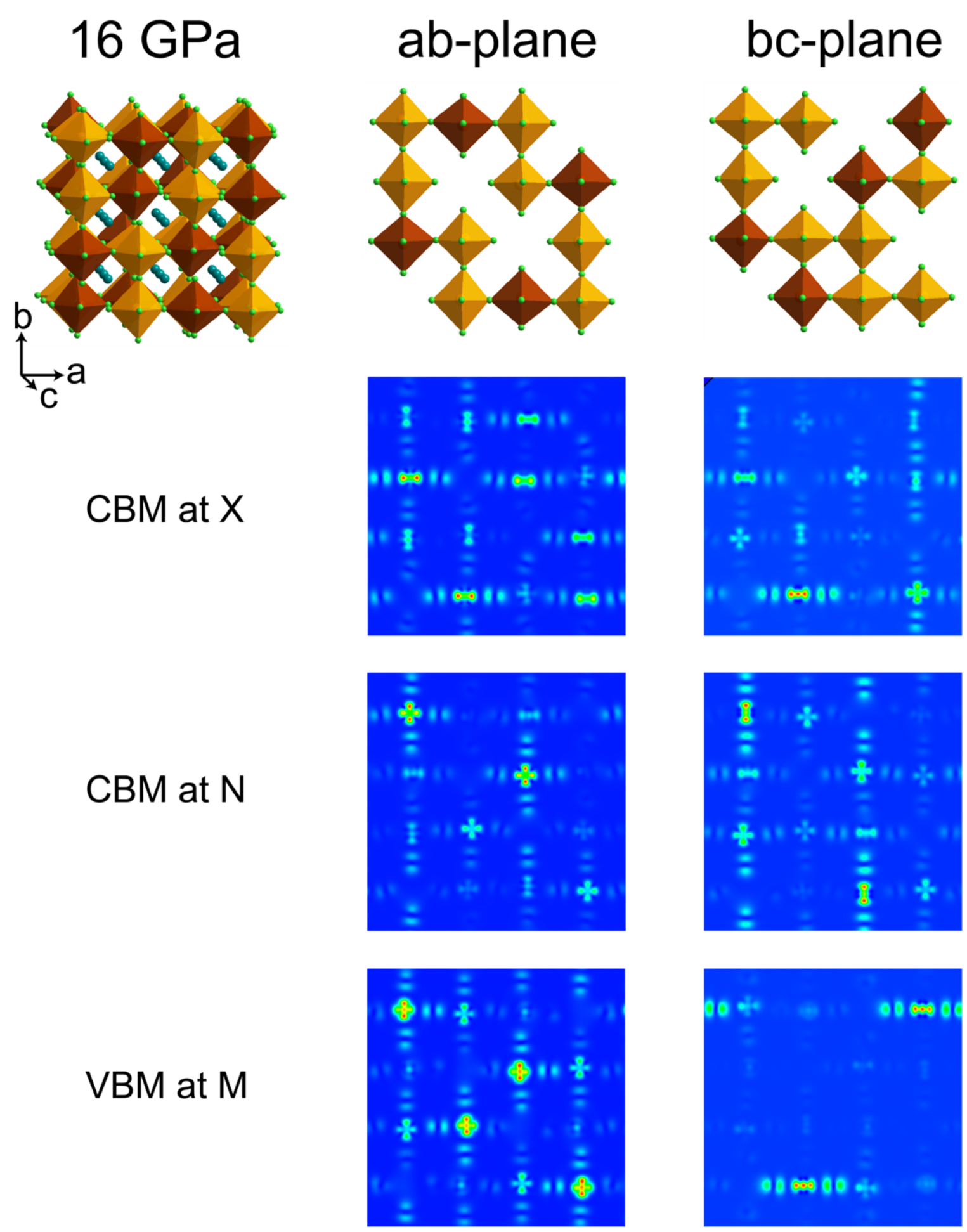

Figure S17. Single-crystal structure of **1** relaxed at 16 GPa (top left) and corresponding electron density maps of the VB and CB at labeled k-points along the *ab* and *bc* planes obtained from our DFT-HSE calculations. Teal, scarlet, gold, and green spheres represent Cs^{+}, Au^{2+}, Au^{3+}, and Cl^{-} ions, respectively. Electron density maps are shown on a rainbow scale where red is the highest density and blue is the lowest density. Maps are scaled for the maximum and minimum density of each image individually, and cannot be compared between images.

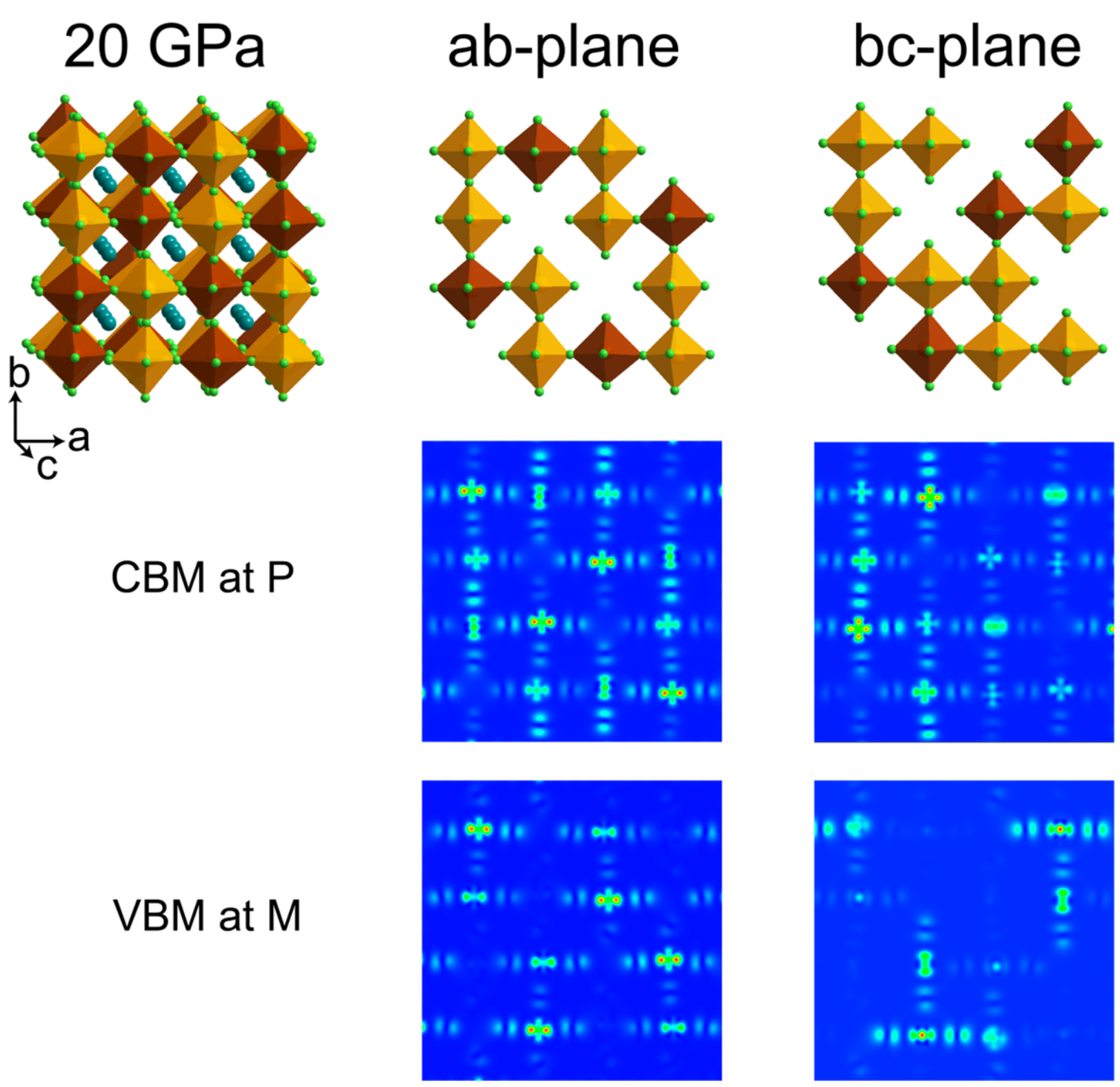

Figure S18. Single-crystal structure of **1** relaxed at 20 GPa (top left) and corresponding electron density maps of the VB and CB at labeled k-points along the *ab* and *bc* planes obtained from our DFT-HSE calculations. Teal, scarlet, gold, and green spheres represent Cs^{+}, Au^{2+}, Au^{3+}, and Cl^{-} ions, respectively. Electron density maps are shown on a rainbow scale where red is the highest density and blue is the lowest density. Maps are scaled for the maximum and minimum density of each image individually, and cannot be compared between images.

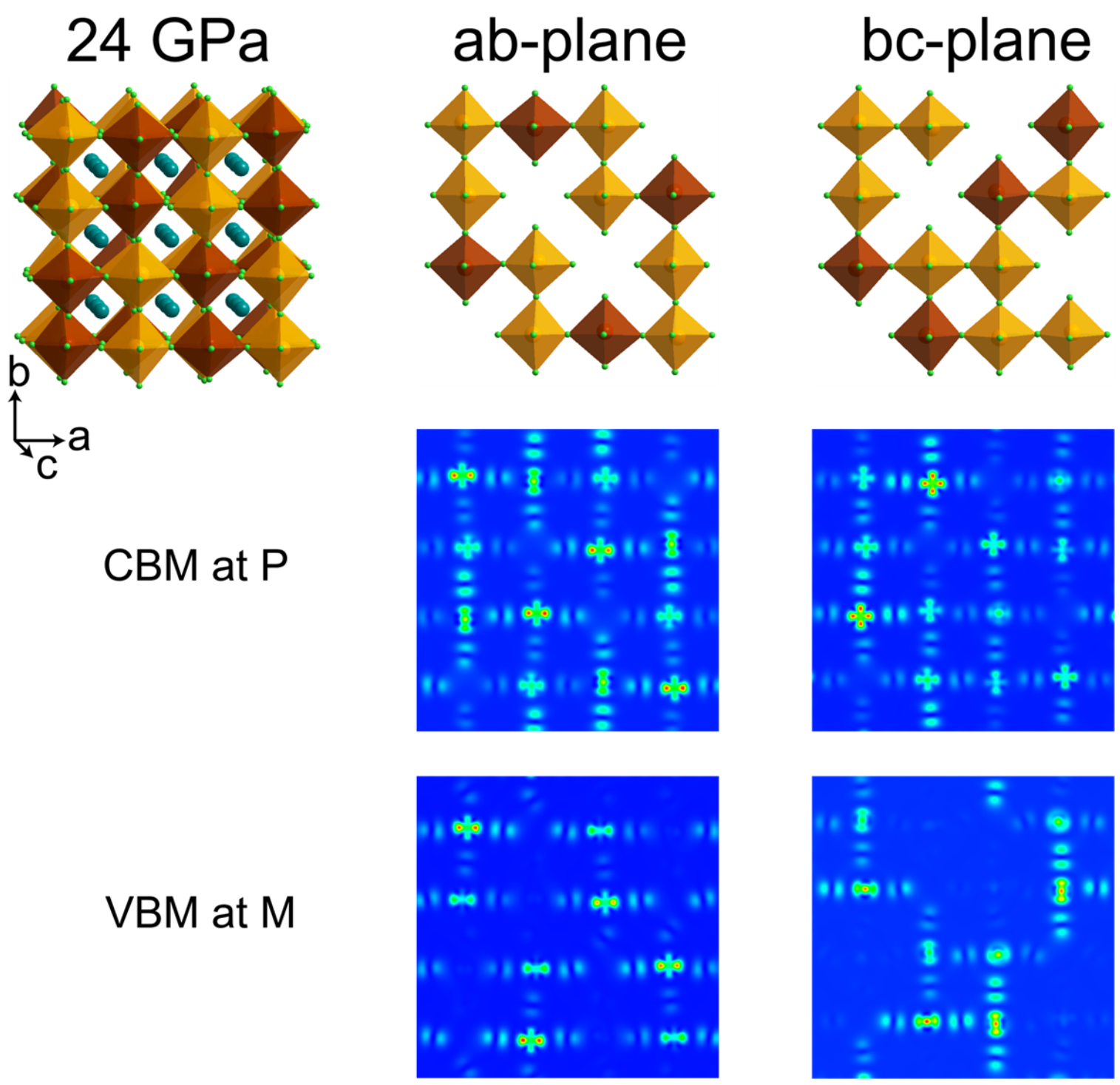

Figure S19. Single-crystal structure of **1** relaxed at 24 GPa (top left) and corresponding electron density maps of the VB and CB at labeled k-points along the *ab* and *bc* planes obtained from our DFT-HSE calculations. Teal, scarlet, gold, and green spheres represent Cs^+, Au^{2+}, Au^{3+}, and Cl^- ions, respectively. Electron density maps are shown on a rainbow scale where red is the highest density and blue is the lowest density. Maps are scaled for the maximum and minimum density of each image individually, and cannot be compared between images.

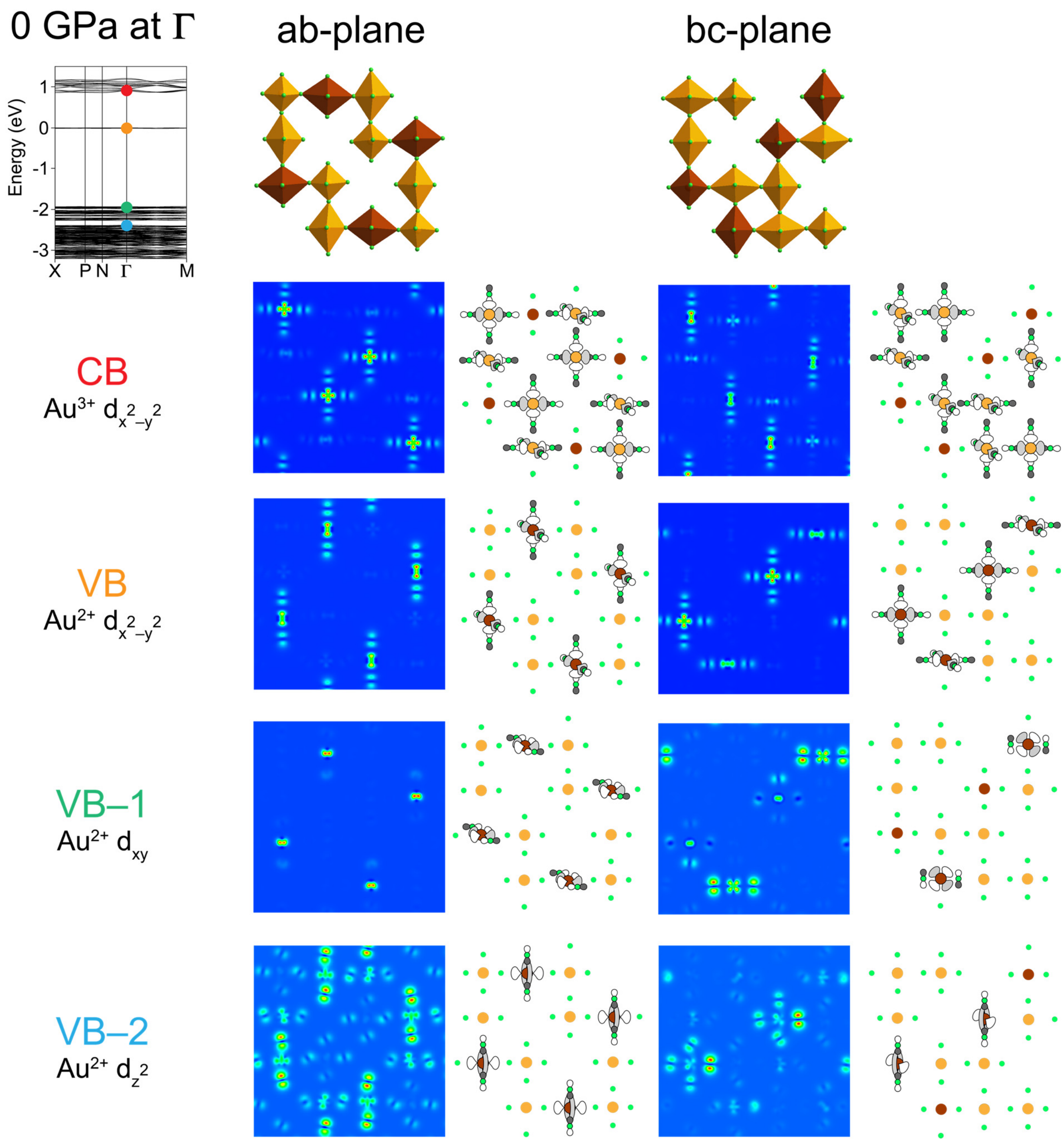

Figure S20. Band structure of **1** relaxed at 0 GPa (top left) and corresponding electron density maps with tentatively assigned orbital schematics of select bands at Γ along the *ab* and *bc* planes obtained from our DFT-HSE calculations. Red, orange, green, and blue dots on the band structure correspond to the CBM, VBM, VBM–1, and VBM–2, respectively. Gray and white orbital lobes represent positive and negative lobes, respectively. Scarlet, gold, and green spheres represent Au^{2+}, Au^{3+}, and Cl^{-} ions, respectively. Electron density maps are shown on a rainbow scale where red is the highest density and blue is the lowest density. Maps are scaled for the maximum and minimum density of each image individually, and cannot be compared between images.

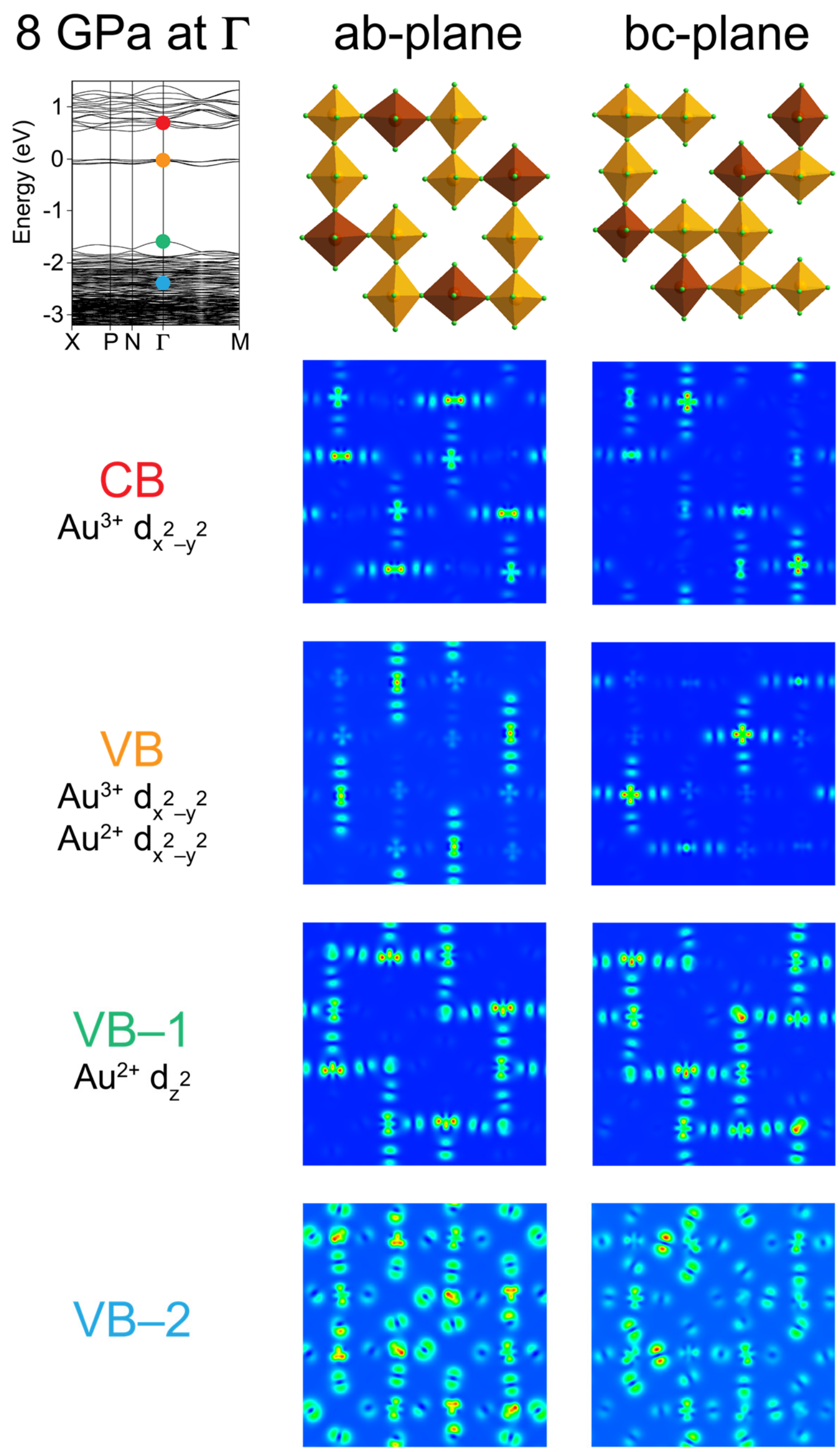

Figure S21. Band structure of **1** relaxed at 8 GPa (top left) and corresponding electron density maps of select bands at Γ along the *ab* and *bc* planes obtained from our DFT-HSE calculations. Red, orange, green, and blue dots on the band structure correspond to the CBM, VBM, VBM–1, and VBM–2, respectively. Scarlet, gold, and green spheres represent Au^{2+}, Au^{3+}, and Cl^{-} ions, respectively. Electron density maps are shown on a rainbow scale where red is the highest density and blue is the lowest density. Maps are scaled for the maximum and minimum density of each image individually, and cannot be compared between images.

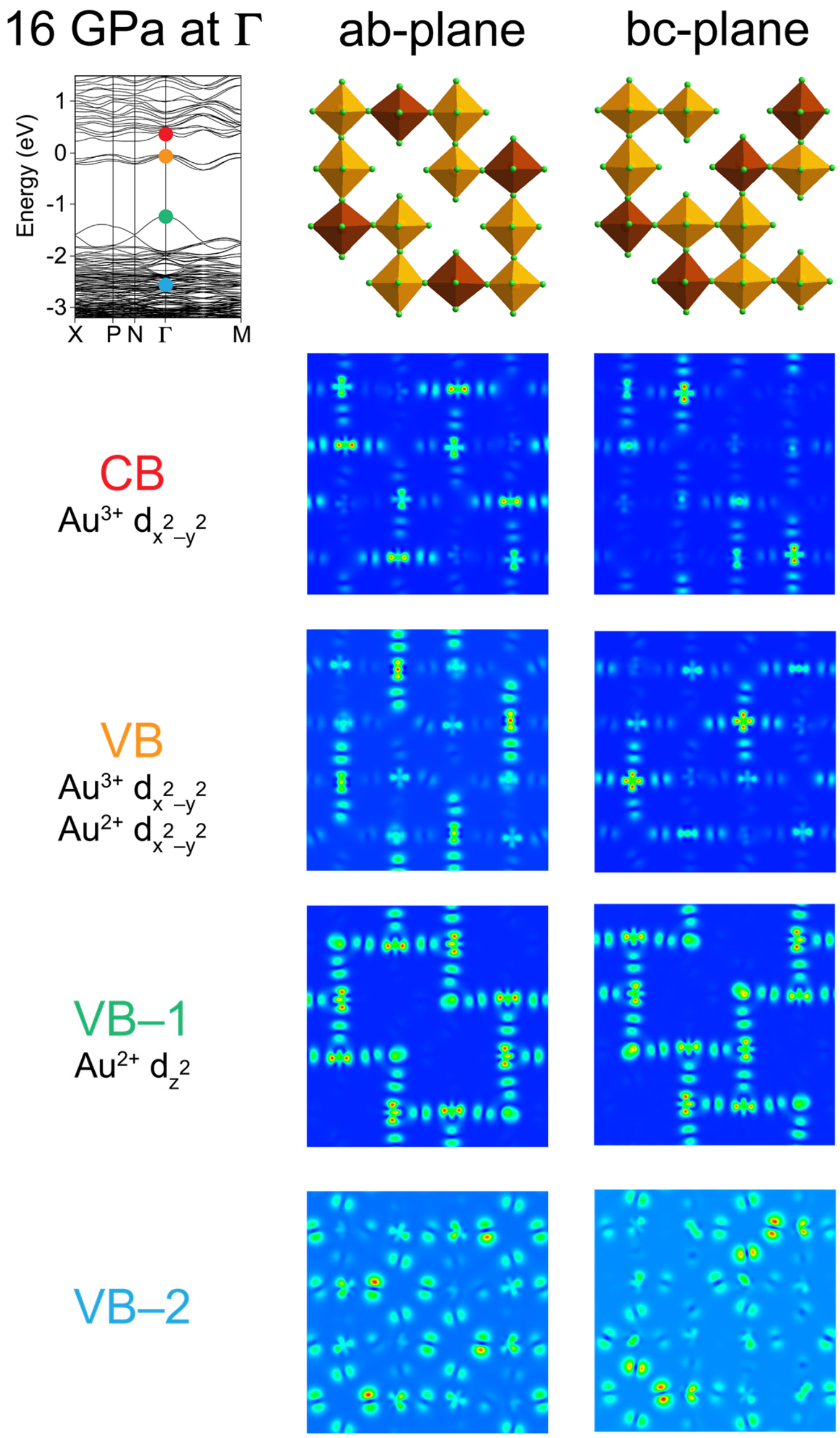

Figure S22. Band structure of **1** relaxed at 16 GPa (top left) and corresponding electron density maps of select bands at Γ along the *ab* and *bc* planes obtained from our DFT-HSE calculations. Red, orange, green, and blue dots on the band structure correspond to the CBM, VBM, VBM–1, and VBM–2, respectively. Scarlet, gold, and green spheres represent Au^{2+}, Au^{3+}, and Cl^{-} ions, respectively. Electron density maps are shown on a rainbow scale where red is the highest density and blue is the lowest density. Maps are scaled for the maximum and minimum density of each image individually, and cannot be compared between images.

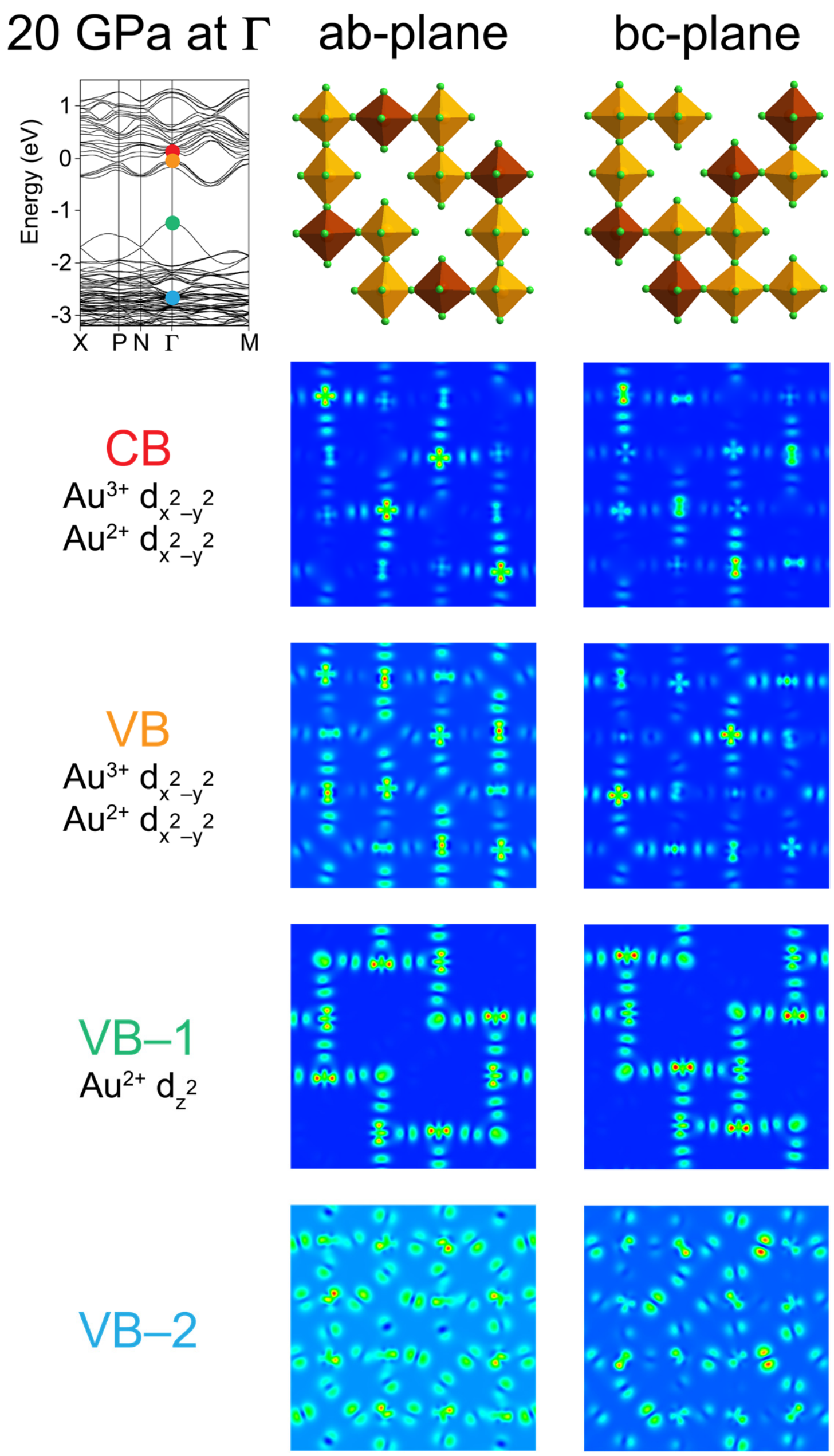

Figure S23. Band structure of **1** relaxed at 20 GPa (top left) and corresponding electron density maps of select bands at Γ along the *ab* and *bc* planes obtained from our DFT-HSE calculations. Red, orange, green, and blue dots on the band structure correspond to the CBM, VBM, VBM–1, and VBM–2, respectively. Scarlet, gold, and green spheres represent Au^{2+}, Au^{3+}, and Cl^{-} ions, respectively. Electron density maps are shown on a rainbow scale where red is the highest density and blue is the lowest density. Maps are scaled for the maximum and minimum density of each image individually, and cannot be compared between images.

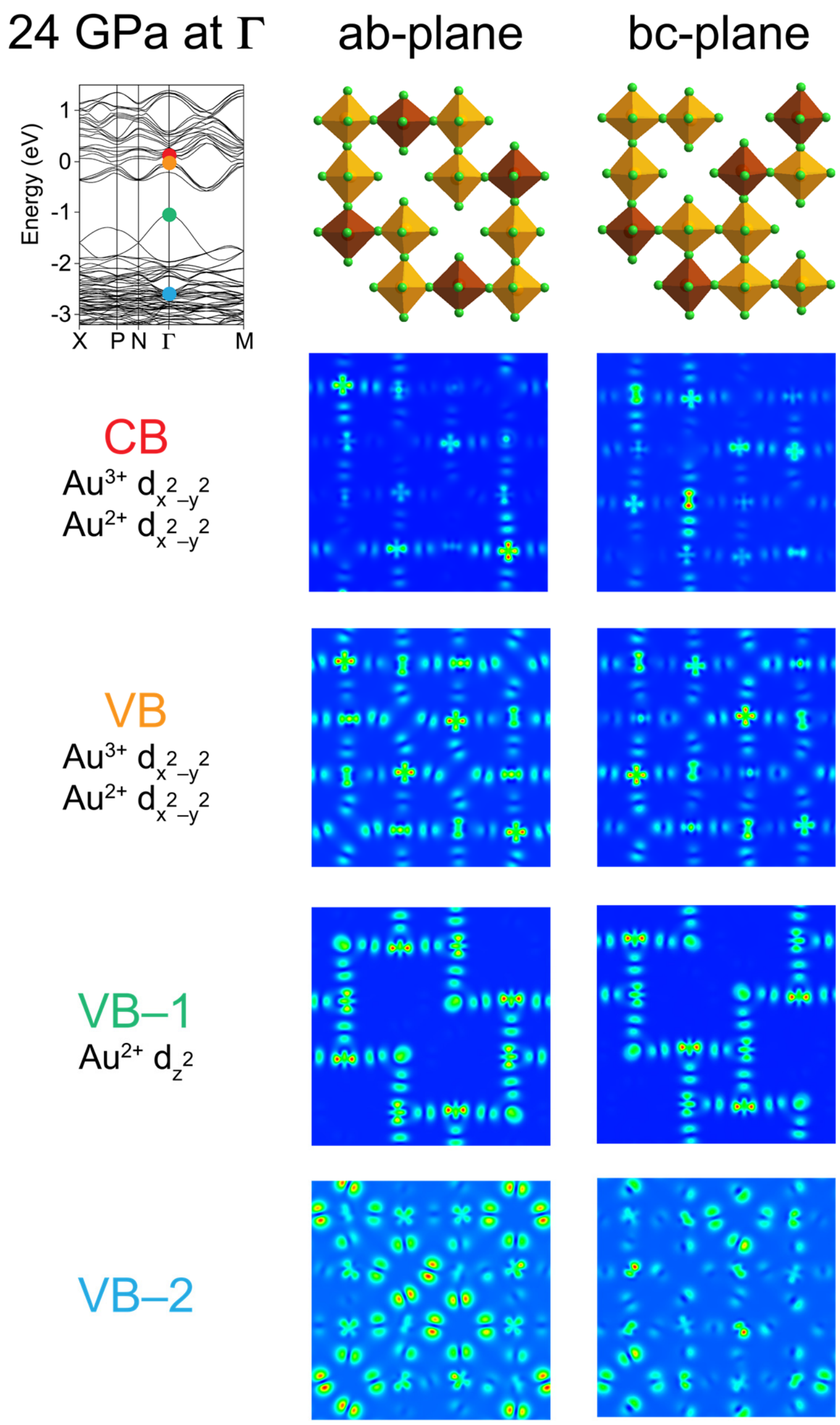

Figure S24. Band structure of **1** relaxed at 24 GPa (top left) and corresponding electron density maps of select bands at Γ along the *ab* and *bc* planes obtained from our DFT-HSE calculations. Red, orange, green, and blue dots on the band structure correspond to the CBM, VBM, VBM–1, and VBM–2, respectively. Scarlet, gold, and green spheres represent Au^{2+}, Au^{3+}, and Cl^{-} ions, respectively. Electron density maps are shown on a rainbow scale where red is the highest density and blue is the lowest density. Maps are scaled for the maximum and minimum density of each image individually, and cannot be compared between images.

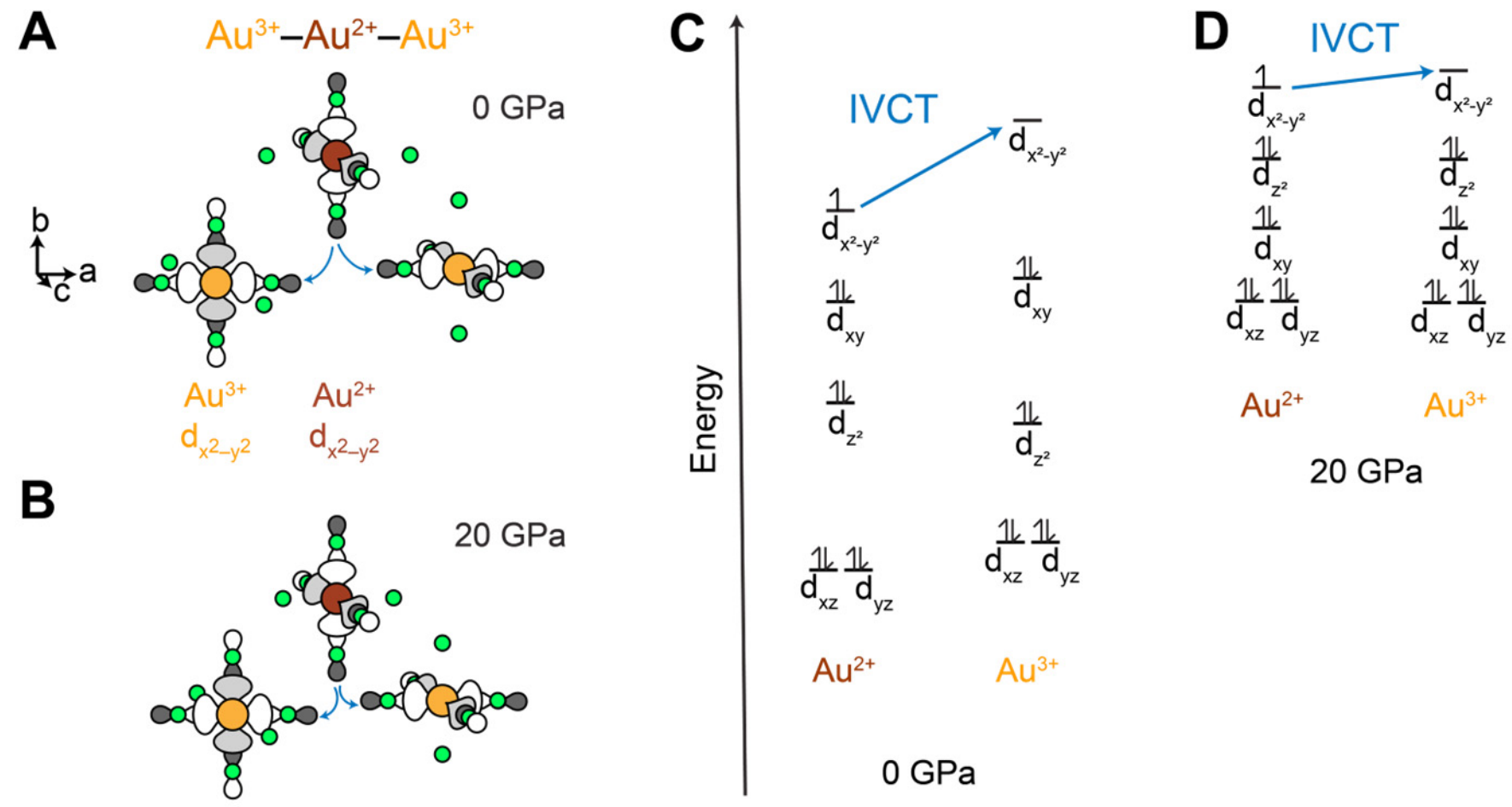

Figure S25. Schematics of the Au $d_{x^2-y^2}$ and Cl p-orbital arrangement of the $Au^{II}Cl_4$ (LUMO) and $Au^{III}Cl_4$ (HOMO) in three square planar subunits arranged around a vacancy in **1** at (**A**) 0 GPa and (**B**) 20 GPa, demonstrating a possible outer-sphere IVCT pathway. Gray and white orbital lobes represent positive and negative lobes, respectively. Scarlet, gold, and green spheres represent Au^{2+}, Au^{3+}, and Cl^{-} ions, respectively. The vacancy compresses and the long Au—Cl bonds contract with pressure. Relative d-orbital energies of **1** at (**C**) 0 GPa and (**D**) 20 GPa, determined using the electron density maps in Figures S20-24. Note that the square-planar d-orbital splitting pattern at 0 GPa changes to the square-pyramidal d-orbital splitting pattern at 20 GPa, when the two long Au—Cl bonds are compressed.

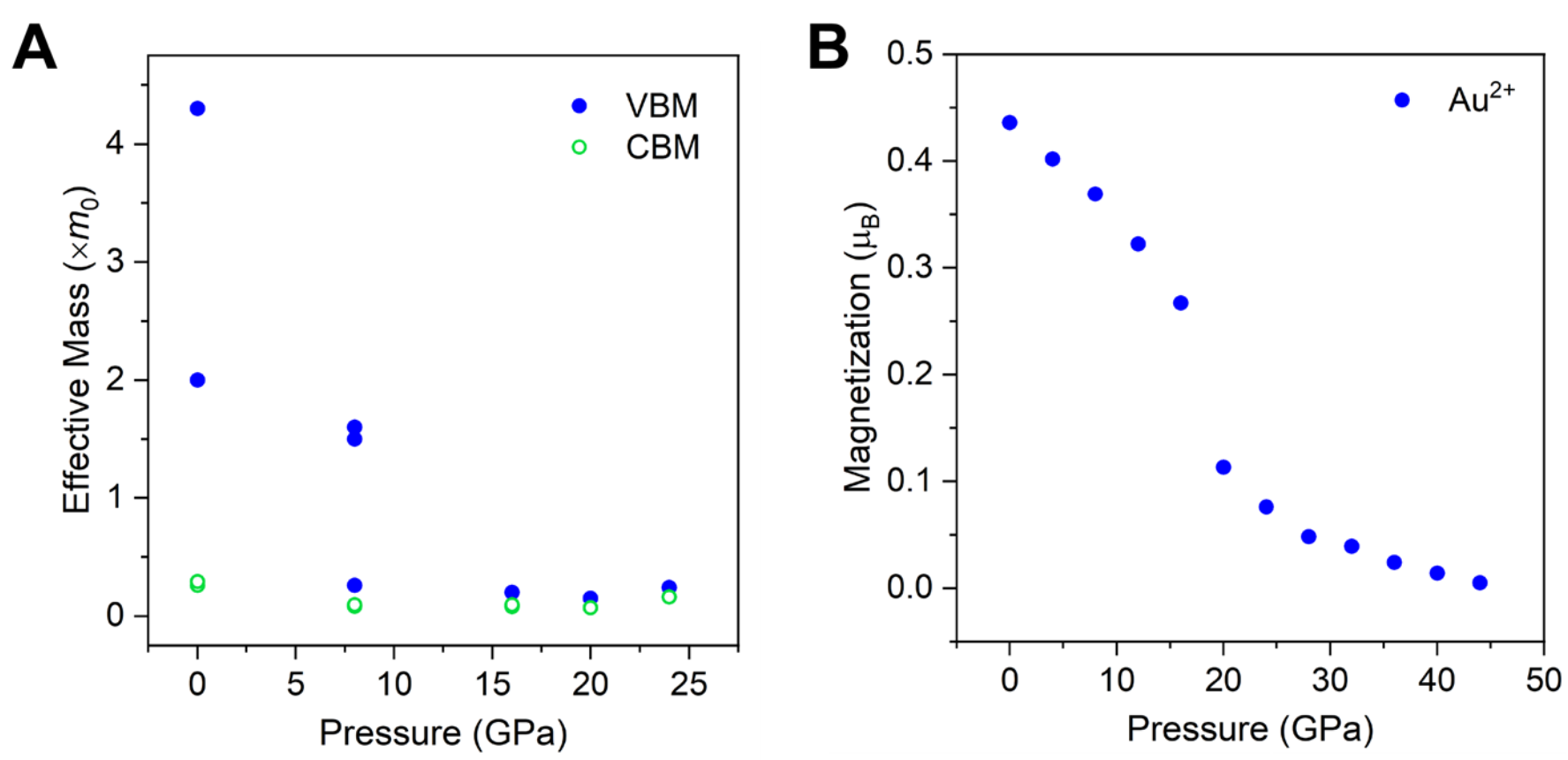

Figure S26. (**A**) Carrier effective masses calculated from the band structures of **1** at the VBM (blue) and CBM (green) at each pressure obtained from our DFT-HSE calculations. Multiple points at certain pressures are from degenerate VBM or CBM *k* points at that pressure. These values are multiples of the electron mass at rest (m_0). (**B**) Calculated magnetization at the Au^{2+} site of **1** relaxed at various pressures. The decrease with increasing pressure corresponds to increased dispersion and reduced spin-splitting of the Au^{2+}-based band.

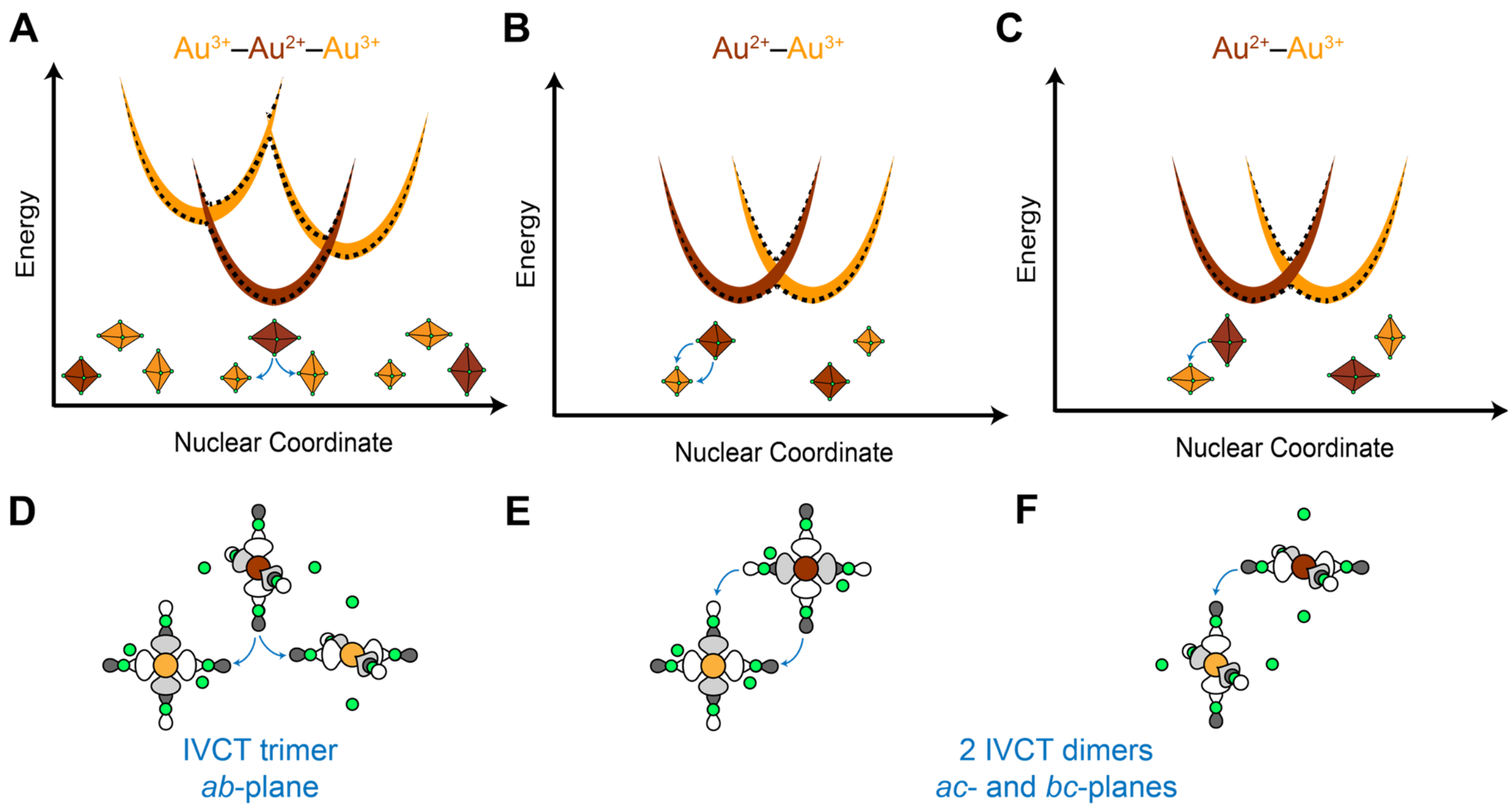

Figure S27. Potential energy curves for IVCT in the smallest repeating molecular across-vacancy trimer and dimer units of **1** along the (**A**) *ab* plane and (**B-C**) *ac* and *bc* planes at ambient pressure. Scarlet and gold wells represent the diabatic potential energy curves of the molecular units of **1** before and after IVCT, respectively. Adiabatic surfaces from mixing of the diabatic states are shown as black dashed curves. The molecular unit schematics were drawn to represent the coordination in structural relaxations at 0 GPa. (**D-F**) Orbital schematics of the Au d and Cl p orbitals of the smallest repeating molecular units depicted in **A-C**, respectively. Gray and white orbital lobes represent positive and negative lobes, respectively. Scarlet, gold, and green spheres represent Au^{2+}, Au^{3+}, and Cl^{-} ions, respectively. Blue arrows represent IVCT.

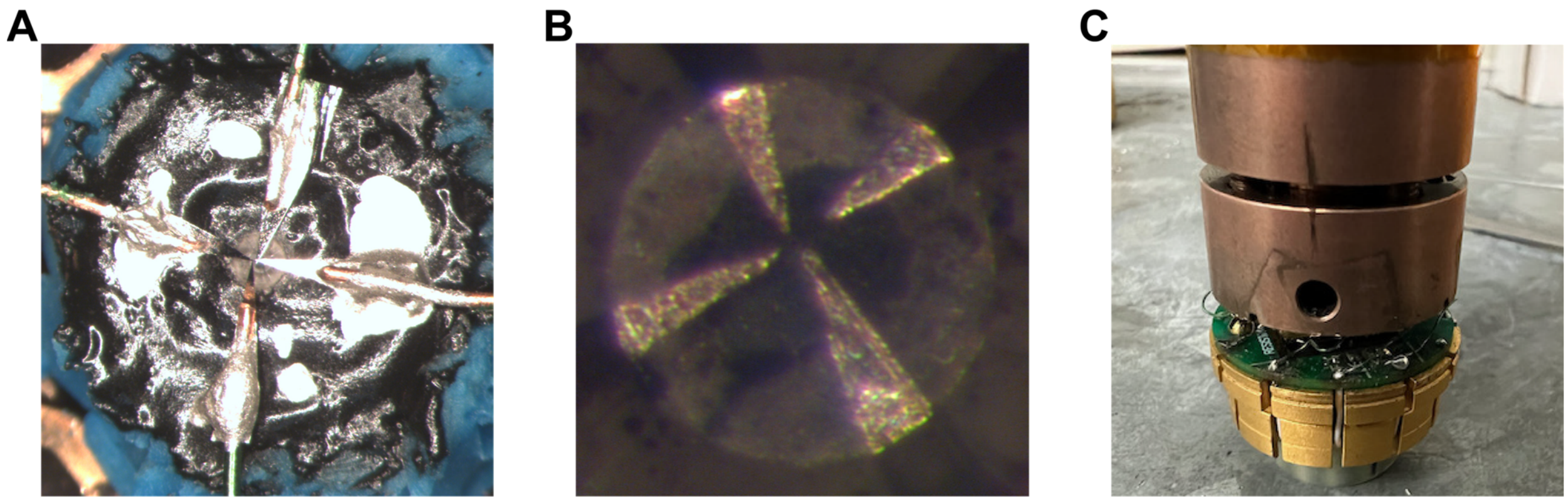

Figure S28. Images of Cu-Be alloy DAC preparation for high-pressure magnetoresistance and Hall effect measurements. (**A**) Image of a rhenium gasket covered in insulating epoxy, on top of which Cu wires are attached to Pt foil leads with Ag paste. (**B**) Image of the Pt leads aligned on the powder-packed sample chamber (dark green) carved out of the cubic-boron nitride epoxy. (**C**) Image of the Cu-Be DAC body placed on a PPMS puck, insulated from it by layers of glass coated in Kapton tape in between. The Cu wires attached to the Pt leads extend from the bottom of the DAC and are soldered onto the PPMS puck attachments. Cu wires have an insulating coating that is removed only on the very ends of the wire, which attach to the Pt foil and the PPMS puck.

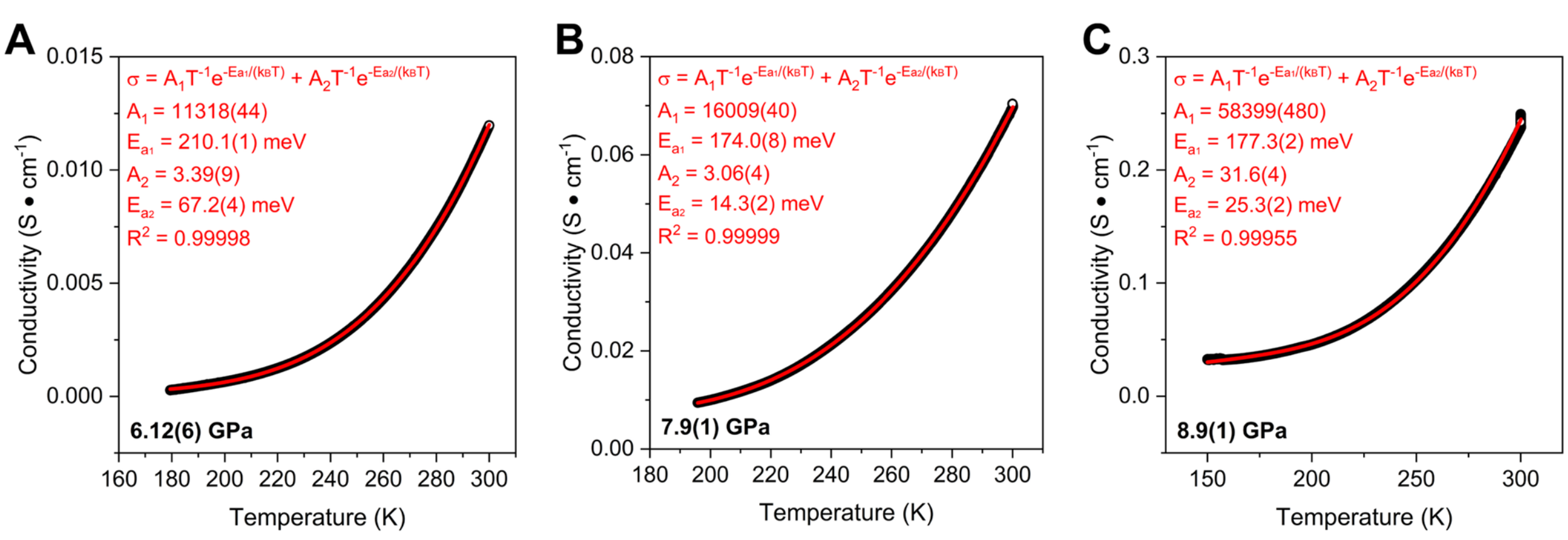

Figure S29. Plots of variable temperature conductivity measurements of **1** at (**A**) 6.12(6) GPa, (**B**) 7.9(1) GPa, and (**C**) 8.9(1) GPa. Samples were cooled to the lowest temperatures and measurements were collected upon heating back to 300 K. Each measurement demonstrated hopping behavior for all temperatures measured. Conductivity data (σ) were fit to two hopping models (for electrons and holes, red curves) to obtain activation energies of conductivity, E_a, and preexponential factors, A, with respect to Boltzmann's constant (k_B) and temperature (T).

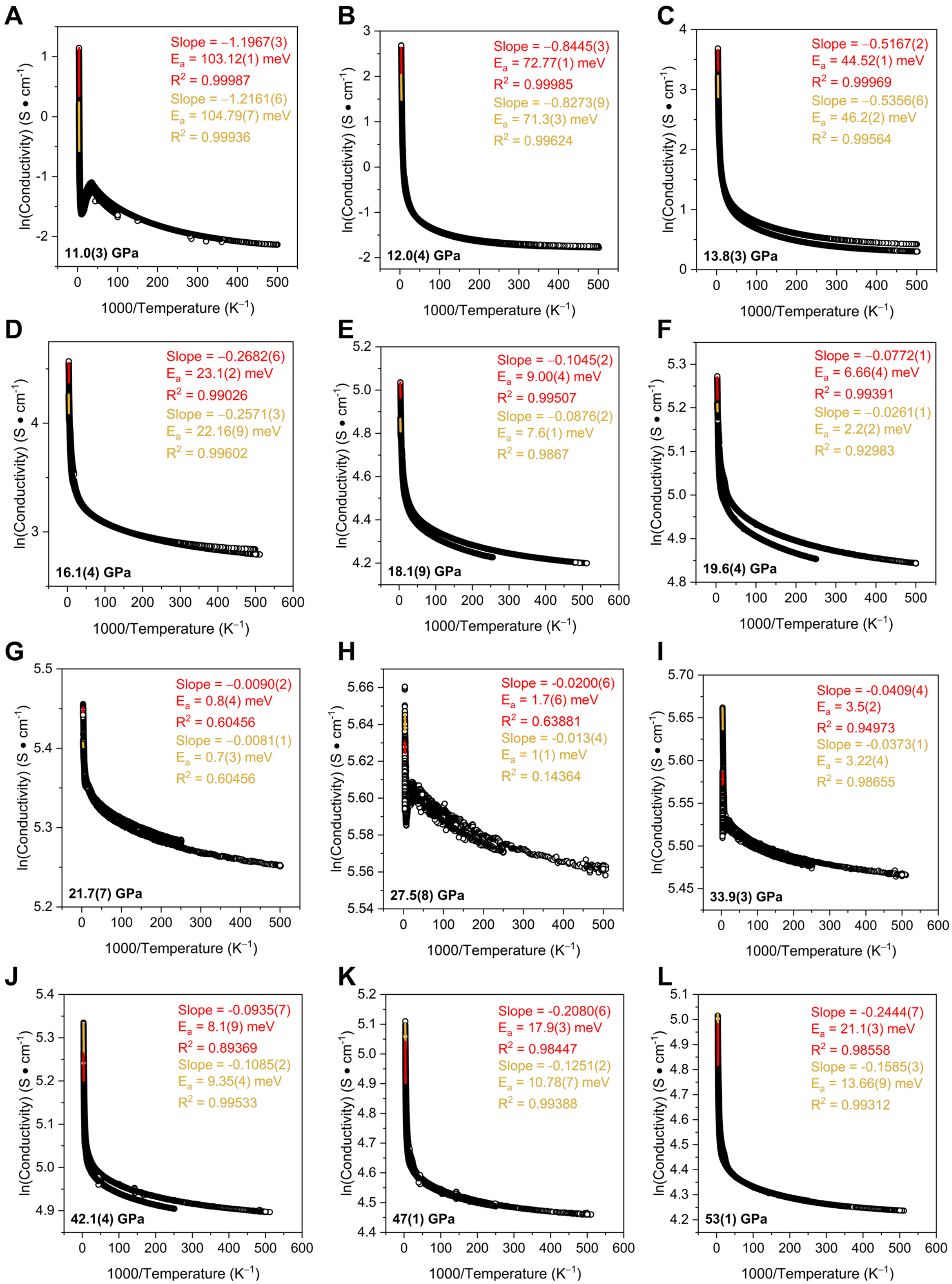

Figure S30. (**A–L**) Arrhenius plots of variable temperature conductivity of **1** at elevated pressures. Data were linearly fit from 300 K to 250 K to obtain activation energies of conductivity, E_a, upon cooling (tan line) and heating (red line).

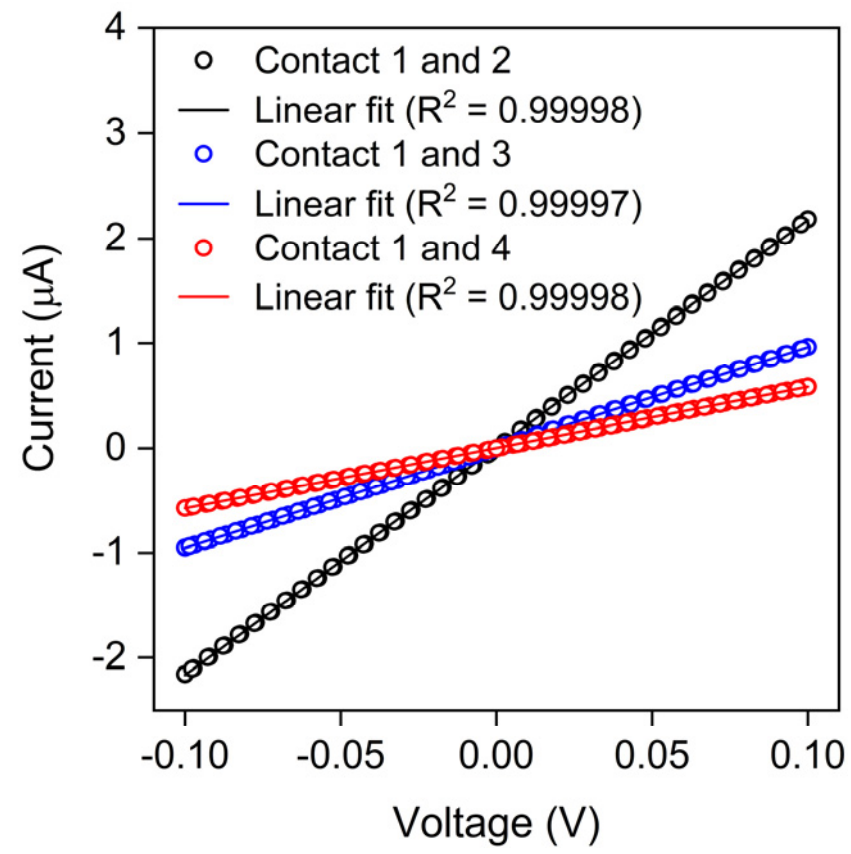

Figure S31. Voltage-current curves between contacts 1 and 2 (black), 1 and 3 (blue), and 1 and 4 (red) of **1** at 6.12(6) GPa, measured simultaneously with data in Figure S29A. Linear fits of each trend indicate strong linear correlation from high R^2 values, demonstrating Ohmic behavior of **1**.

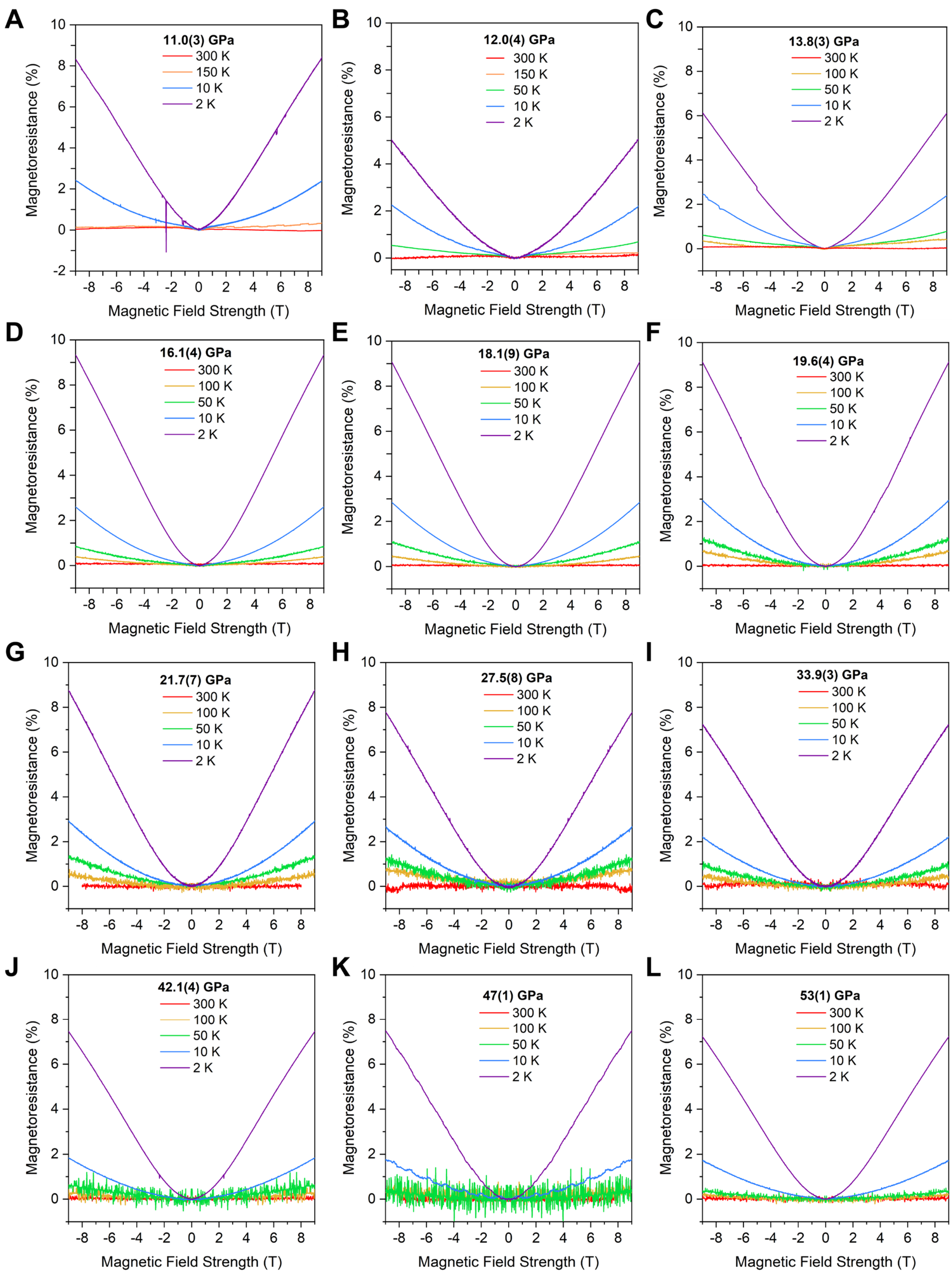

Figure S32 (**A-L**) Magnetoresistance (percentage increase) of **1** with pressure. Red, orange, yellow, green, blue, and purple lines correspond to measurements collected at 300 K, 150 K, 100 K, 50 K, 10 K, and 2 K, respectively. All data are symmetrized with respect to the magnetic field.

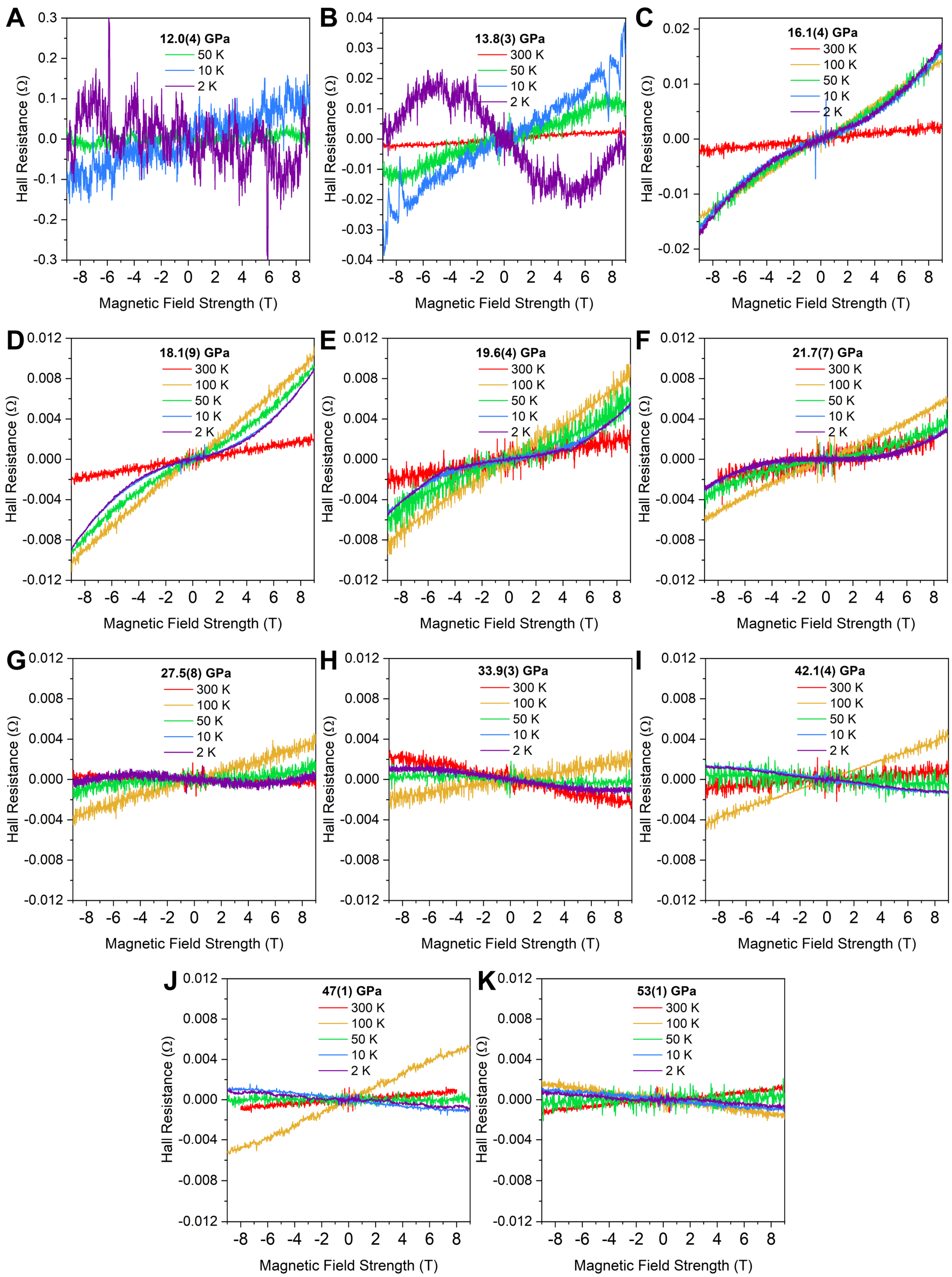

Figure S33. (**A-K**) Hall resistance of **1** with pressure. Red, yellow, green, blue, and purple lines correspond to measurements collected at 300 K, 100 K, 50 K, 10 K, and 2 K, respectively. Positive and negative slopes correspond to hole and electron dominance, respectively, in a two-carrier model. All data are anti-symmetrized with respect to the magnetic field.

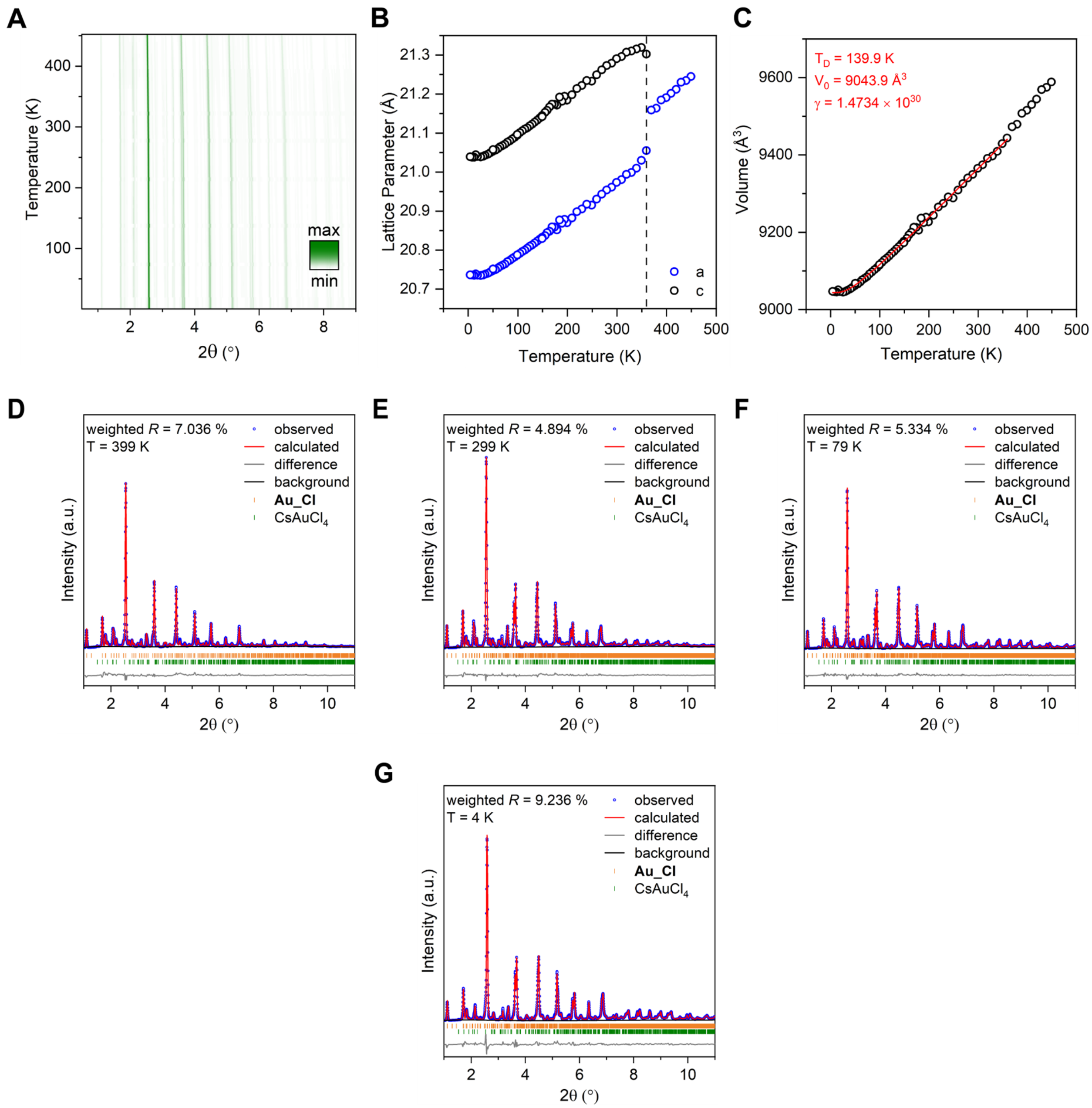

Figure S34. (**A**) Variable-temperature (VT) PXRD patterns of **1** from 4 K to 450 K (step size: 5-10 K; X-ray wavelength: 0.1665 Å). The PXRD intensities are normalized between 0 and 1 for each pattern. (**B**) Lattice parameters and (**C**) unit-cell volumes of **1** with respect to temperature for the diffraction patterns in **A**, determined through Rietveld refinement. The dotted line represents a high-temperature phase transition to cubic structure, consistent with the initial report of **1**. The red line corresponds to a Debye-Grüneisen fit of the volume-temperature trend of the tetragonal phase of **1** from 4 K to 359 K. T_D, V_0, and γ correspond to the Debye temperature (in K), the unit-cell volume at 0 K, and the Grüneisen parameter, respectively. Rietveld refinement of **1** with a minor $CsAuCl_4$ impurity at (**D**) 399 K, (**E**) 299 K, (**F**) 79 K, and (**G**) 4.1 K. Experimental data (blue), calculated diffraction patterns from the refined structures (red), the difference between observed and calculated diffraction patterns (gray), the background (blue) and the hkl reflections of the refined structures (orange and green for **1** (**Au_Cl**) and $CsAuCl_4$, respectively) are shown.

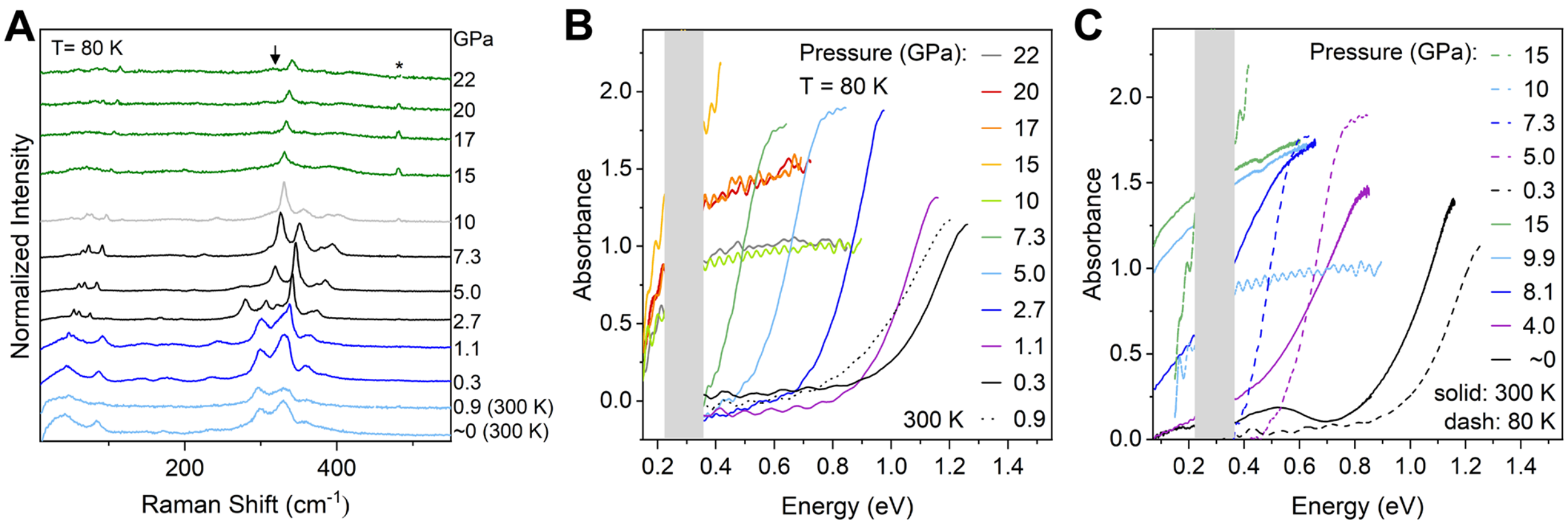

Figure S35. (**A**) Variable-pressure Raman spectra of **1** at 80 K. Blue, black, gray, and green traces correspond to the α ($I4_1cd$), β ($Iba2$), β + γ, and γ ($Ia\overline{3}d$) phases of **1** determined from diffraction measurements at room temperature. (**B**) Variable-pressure absorption measurements of **1** at 80 K. (**C**) Comparison between variable-pressure absorption measurements at 300 K and 80 K to highlight bandgap widening at lower temperatures. The shaded regions obscure strong diamond absorption.

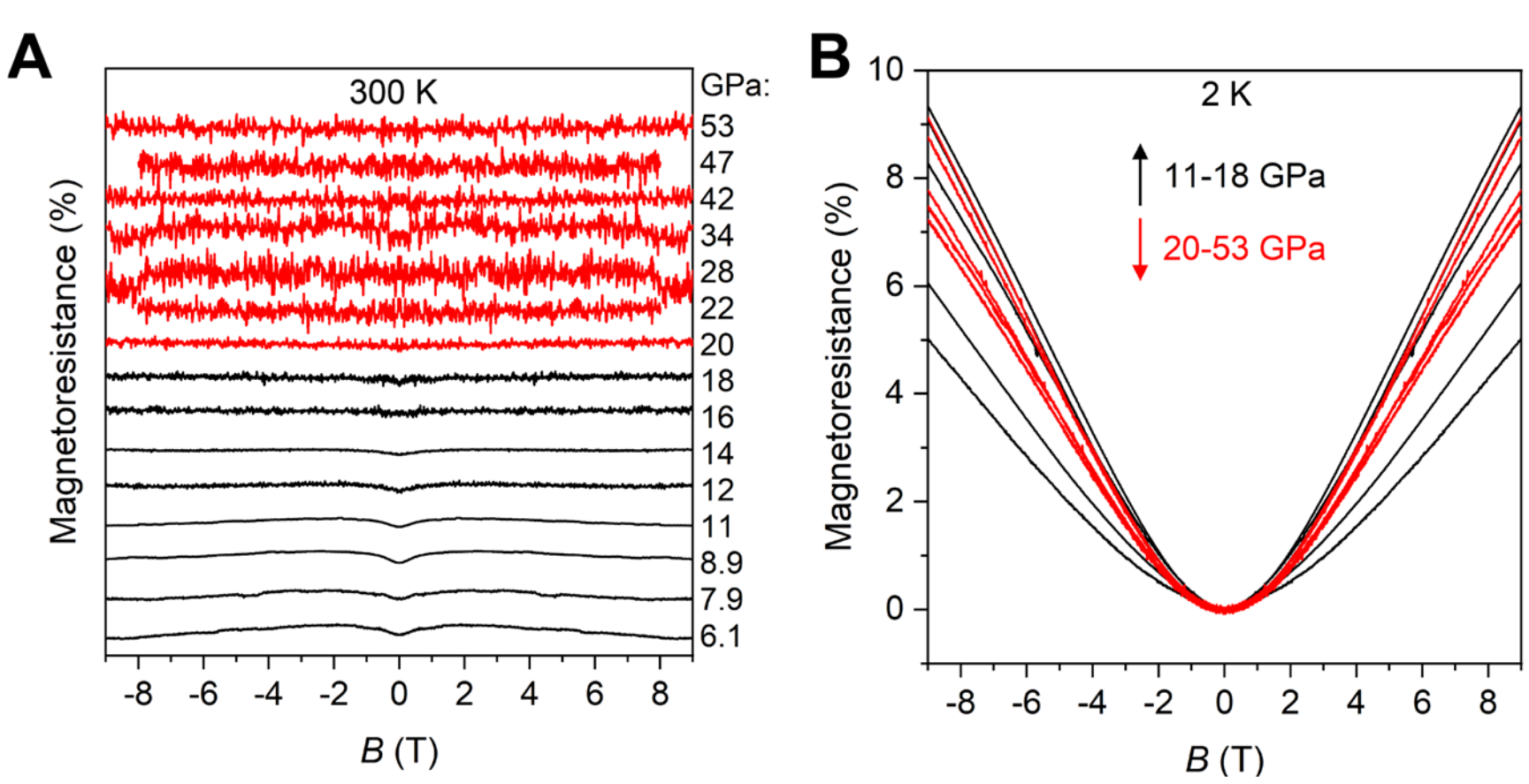

Figure S36. (**A**) Magnetoresistance of **1** at different pressures with an applied magnetic field (B) at 300 K, stacked in a waterfall plot with 0.35% y offset for each pressure to highlight a low-field (~1 T) feature in the black traces. (**B**) Magnetoresistance (% increase) of **1** at different pressures with applied magnetic field (B) at 2 K, showing an increase from 11-18 GPa and a subsequent decrease from 20-53 GPa.

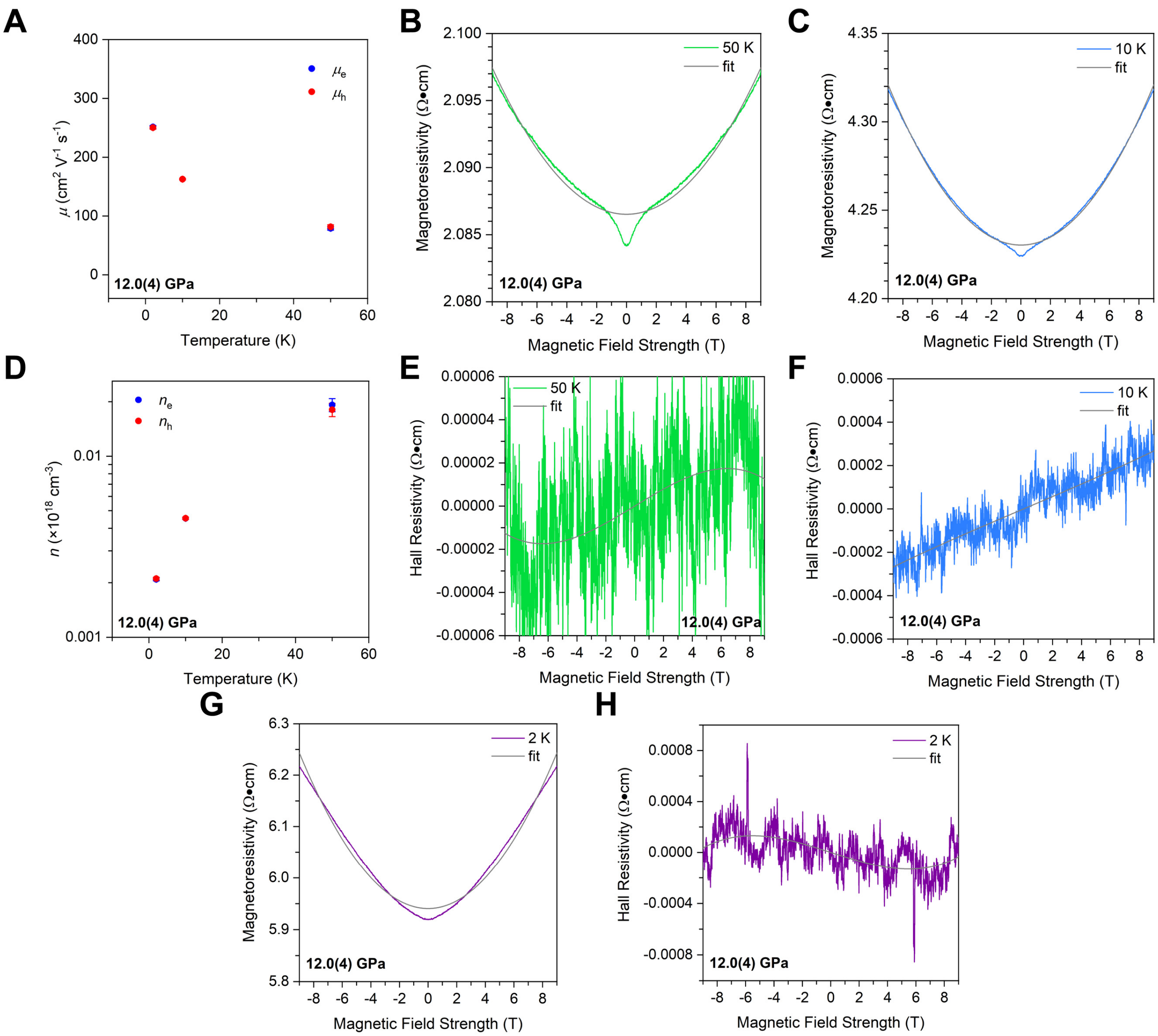

Figure S37. Temperature dependence of (**A**) carrier mobilities (μ) and (**D**) carrier densities (*n*) in **1** at 12.0(4) GPa determined through global fitting of magnetoresistivity and Hall resistivity, calculated from magnetoresistance and Hall effect measurements. Subscripts of e and h correspond to properties of electrons and holes, respectively. (**B-C, G**) Magnetoresistivity of **1** at 12.0(4) GPa collected at 50 K, 10 K, and 2 K, respectively. (**E-F, H**) Hall resistivity of **1** at 12.0(4) GPa collected at 50 K, 10 K, and 2 K, respectively. Gray lines correspond to global fitting of magnetoresistivity and Hall resistivity to a two-carrier model.

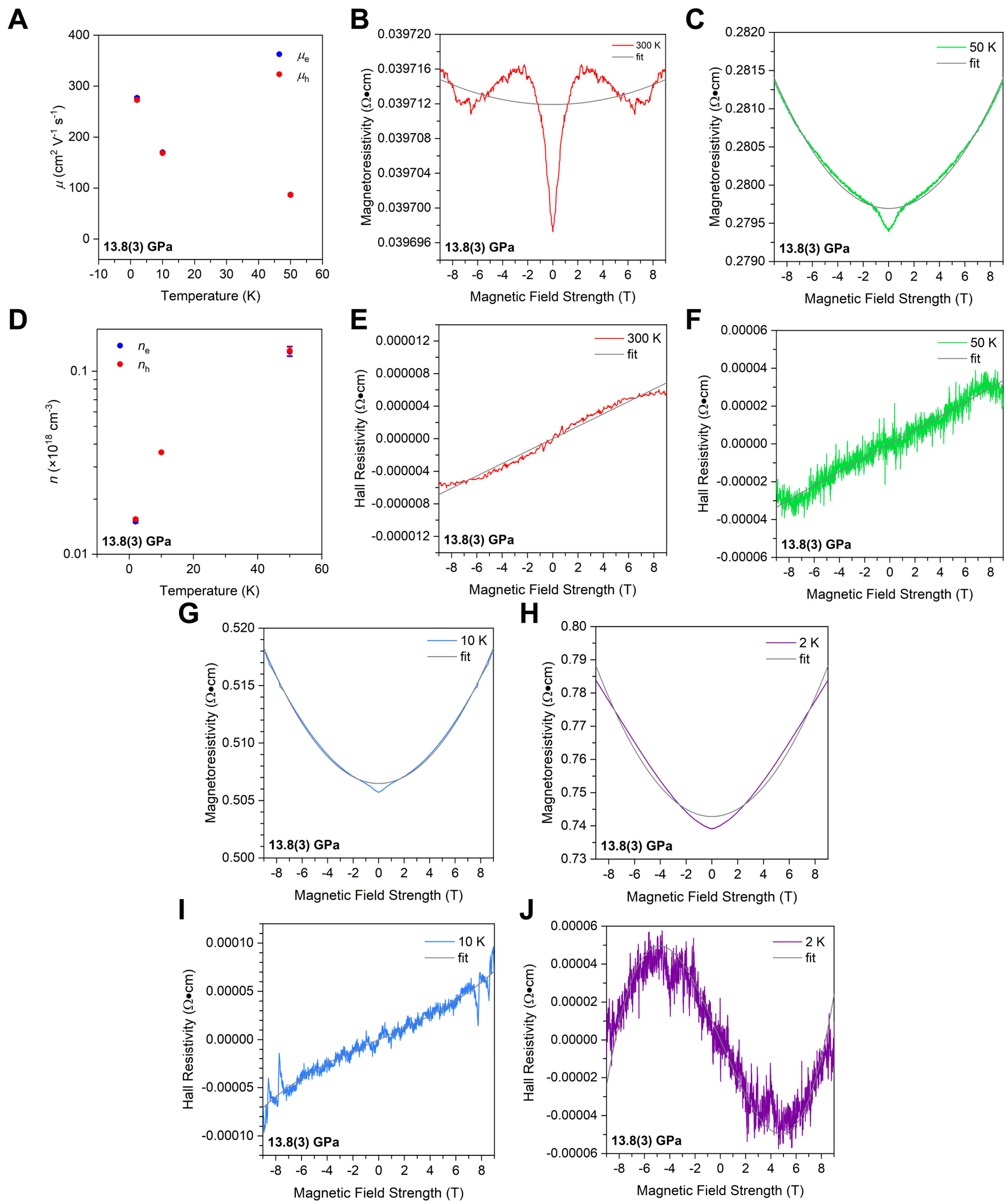

Figure S38. Temperature dependence of (**A**) carrier mobilities (μ) and (**D**) carrier densities (*n*) in **1** at 13.8(3) GPa determined through global fitting of magnetoresistivity and Hall resistivity, calculated from magnetoresistance and Hall effect measurements. Subscripts of e and h correspond to properties of electrons and holes, respectively. (**B-C, G-H**) Magnetoresistivity of **1** at 13.8(3) GPa collected at 300 K, 50 K, 10 K, and 2 K, respectively. (**E-F, I-J**) Hall resistivity of **1** at 13.8(3) GPa collected at 300 K, 50 K, 10 K, and 2 K, respectively. Gray lines correspond to global fitting of magnetoresistivity and Hall resistivity to a two-carrier model.

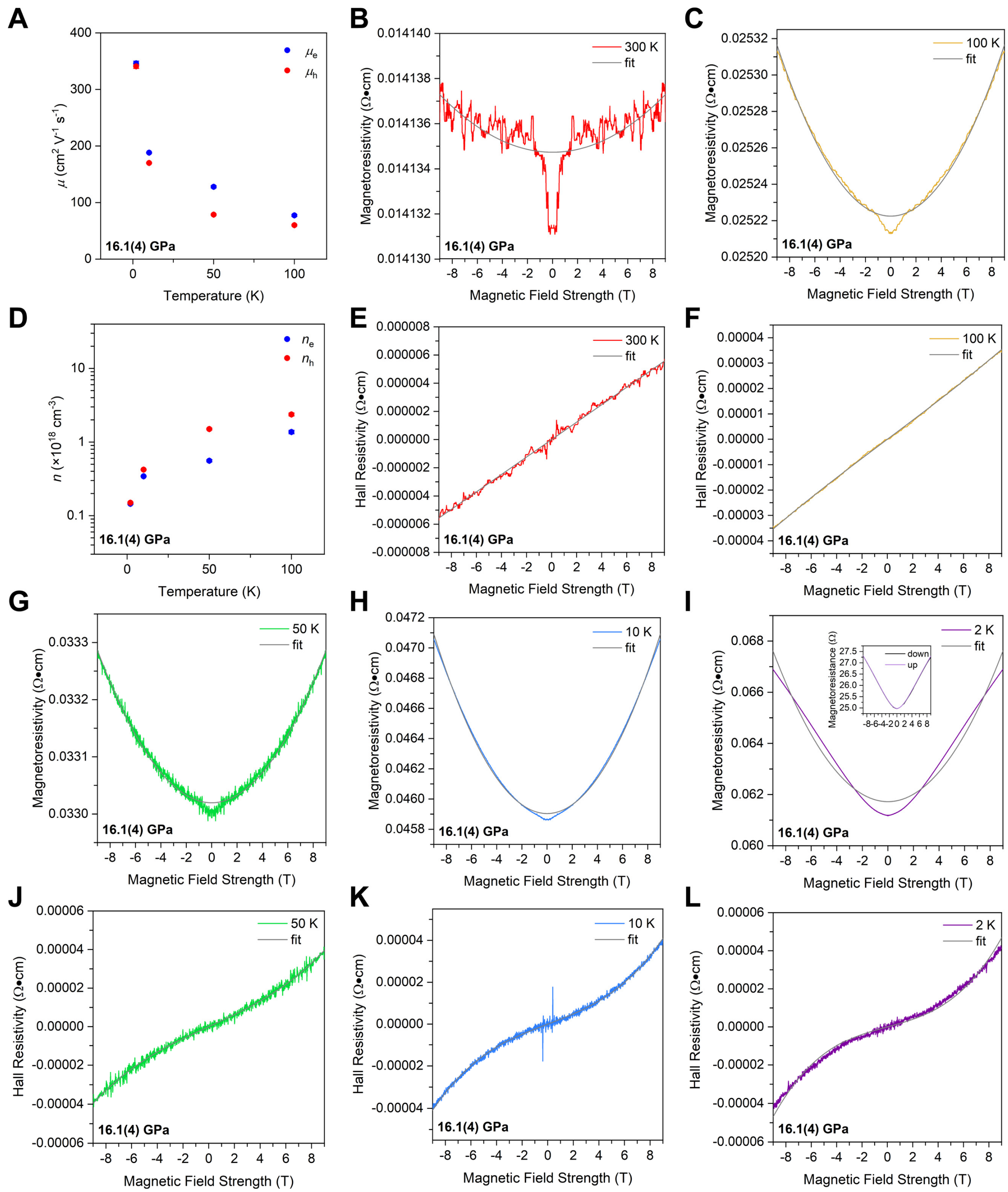

Figure S39. Temperature dependence of (**A**) carrier mobilities (μ) and (**D**) carrier densities (*n*) in **1** at 16.1(4) GPa determined through global fitting of magnetoresistivity and Hall resistivity, calculated from magnetoresistance and Hall effect measurements. Subscripts of e and h correspond to properties of electrons and holes, respectively. (**B-C, G-I**) Magnetoresistivity of **1** at 16.1(4) GPa collected at 300 K, 100 K, 50 K, 10 K, and 2 K, respectively. Inset demonstrates no hysteresis with decreasing (black) and increasing (lilac) magnetic field. (**E-F, J-L**) Hall resistivity of **1** at 16.1(4) GPa collected at 300 K, 100 K, 50 K, 10 K, and 2 K, respectively. Gray lines correspond to global fitting of magnetoresistivity and Hall resistivity to a two-carrier model.

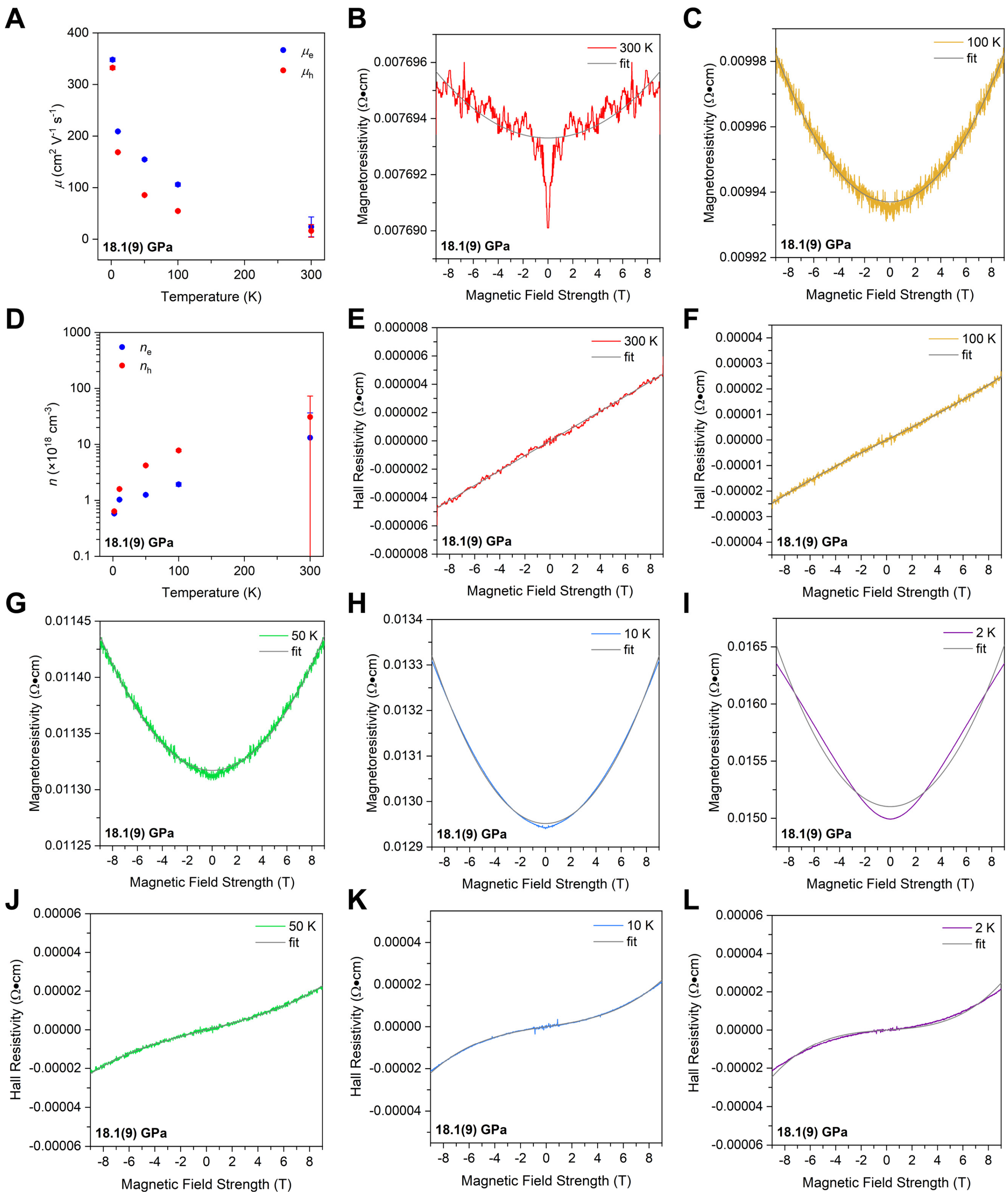

Figure S40. Temperature dependence of (**A**) carrier mobilities (μ) and (**D**) carrier densities (*n*) in **1** at 18.1(9) GPa determined through global fitting of magnetoresistivity and Hall resistivity, calculated from magnetoresistance and Hall effect measurements. Subscripts of e and h correspond to properties of electrons and holes, respectively. (**B-C, G-I**) Magnetoresistivity of **1** at 18.1(9) GPa collected at 300 K, 100 K, 50 K, 10 K, and 2 K, respectively. (**E-F, J-L**) Hall resistivity of **1** at 18.1(9) GPa collected at 300 K, 100 K, 50 K, 10 K, and 2 K, respectively. Gray lines correspond to global fitting of magnetoresistivity and Hall resistivity to a two-carrier model.

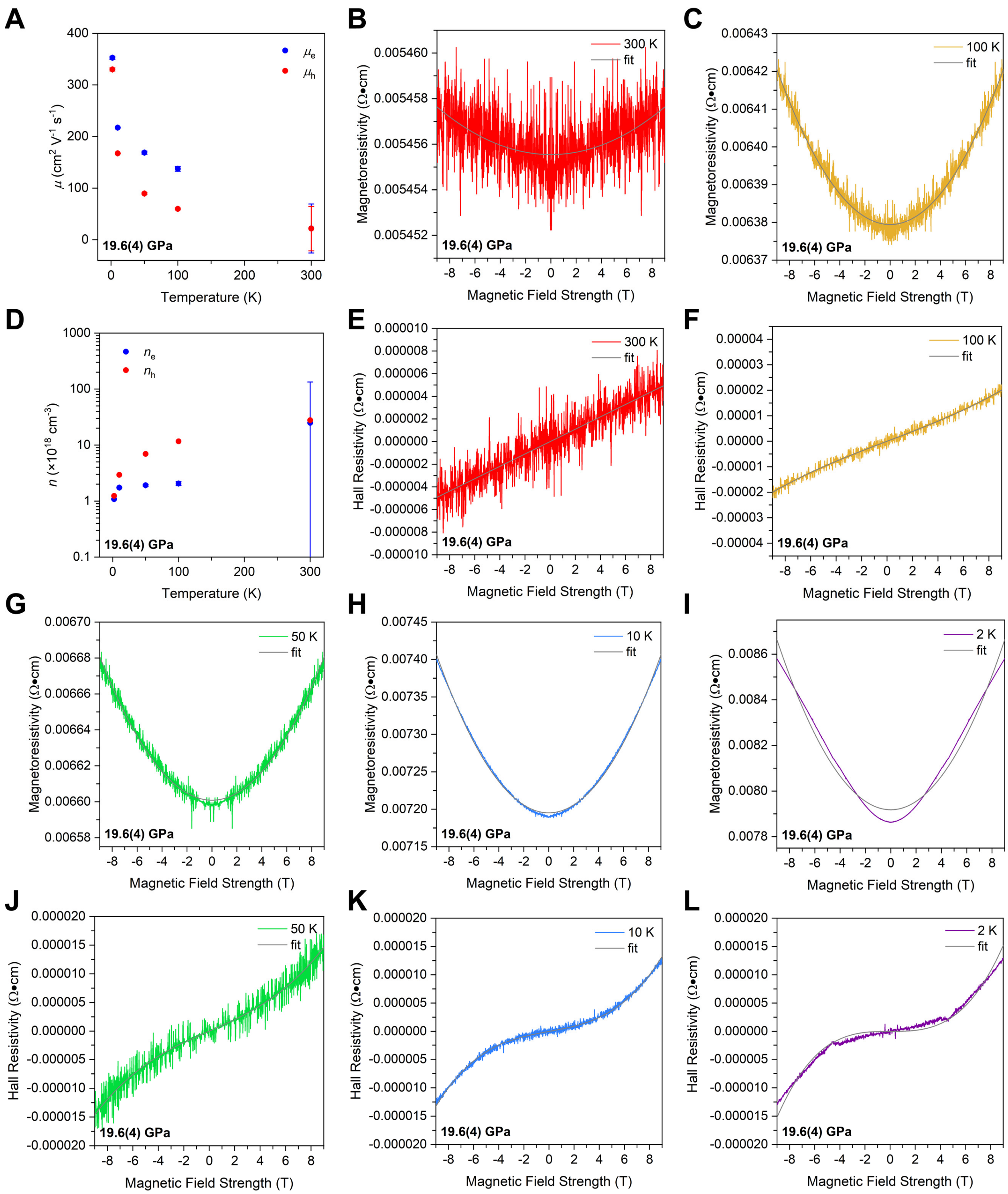

Figure S41. Temperature dependence of (**A**) carrier mobilities (μ) and (**D**) carrier densities (*n*) in **1** at 19.6(4) GPa determined through global fitting of magnetoresistivity and Hall resistivity, calculated from magnetoresistance and Hall effect measurements. Subscripts of e and h correspond to properties of electrons and holes, respectively. (**B-C, G-I**) Magnetoresistivity of **1** at 19.6(4) GPa collected at 300 K, 100 K, 50 K, 10 K, and 2 K, respectively. (**E-F, J-L**) Hall resistivity of **1** at 19.6(4) GPa collected at 300 K, 100 K, 50 K, 10 K, and 2 K, respectively. Gray lines correspond to global fitting of magnetoresistivity and Hall resistivity to a two-carrier model.

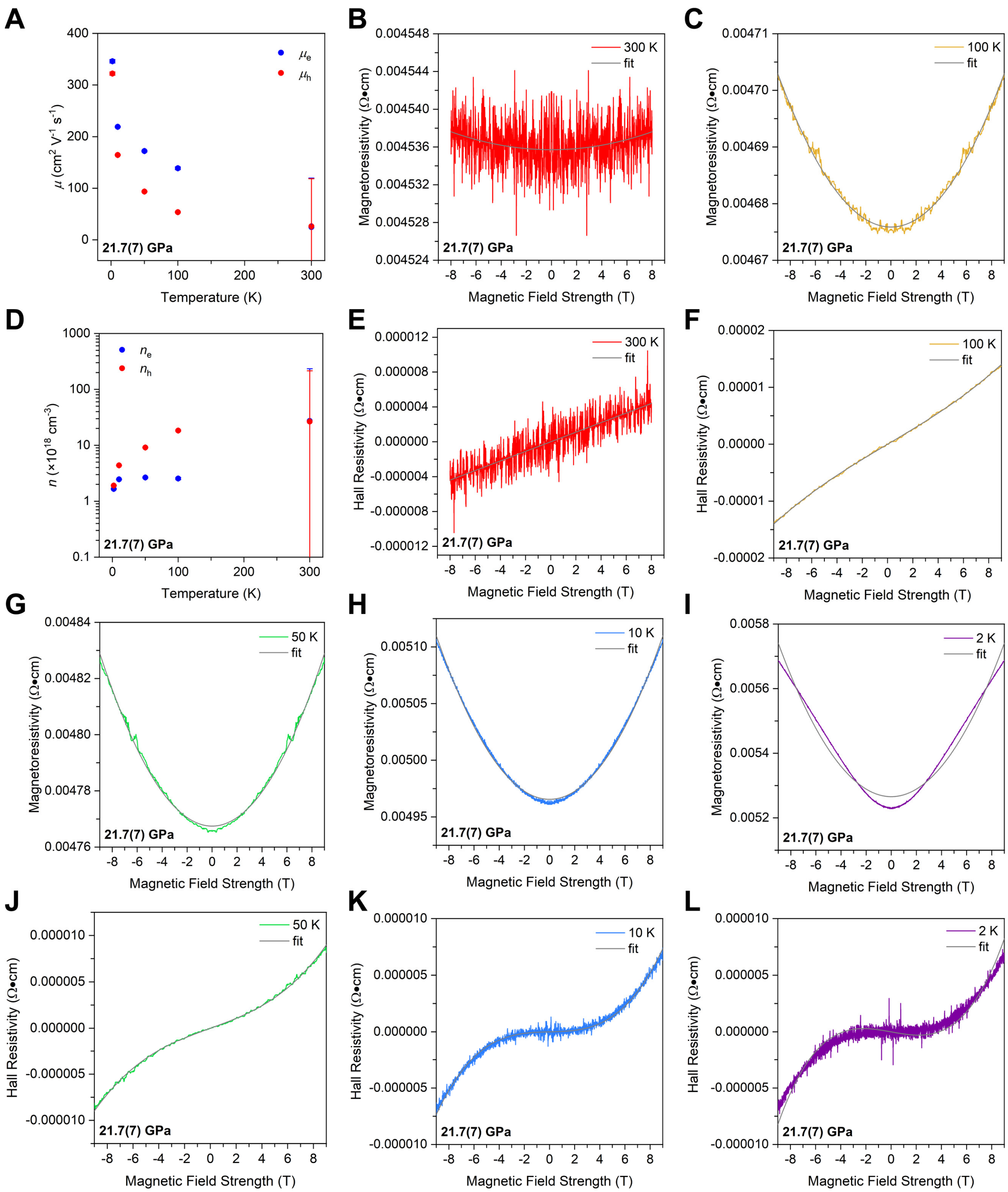

Figure S42. Temperature dependence of (**A**) carrier mobilities (μ) and (**D**) carrier densities (*n*) in **1** at 21.7(7) GPa determined through global fitting of magnetoresistivity and Hall resistivity, calculated from magnetoresistance and Hall effect measurements. Subscripts of e and h correspond to properties of electrons and holes, respectively. (**B-C, G-I**) Magnetoresistivity of **1** at 21.7(7) GPa collected at 300 K, 100 K, 50 K, 10 K, and 2 K, respectively. (**E-F, J-L**) Hall resistivity of **1** at 21.7(7) GPa collected at 300 K, 100 K, 50 K, 10 K, and 2 K, respectively. Gray lines correspond to global fitting of magnetoresistivity and Hall resistivity to a two-carrier model.

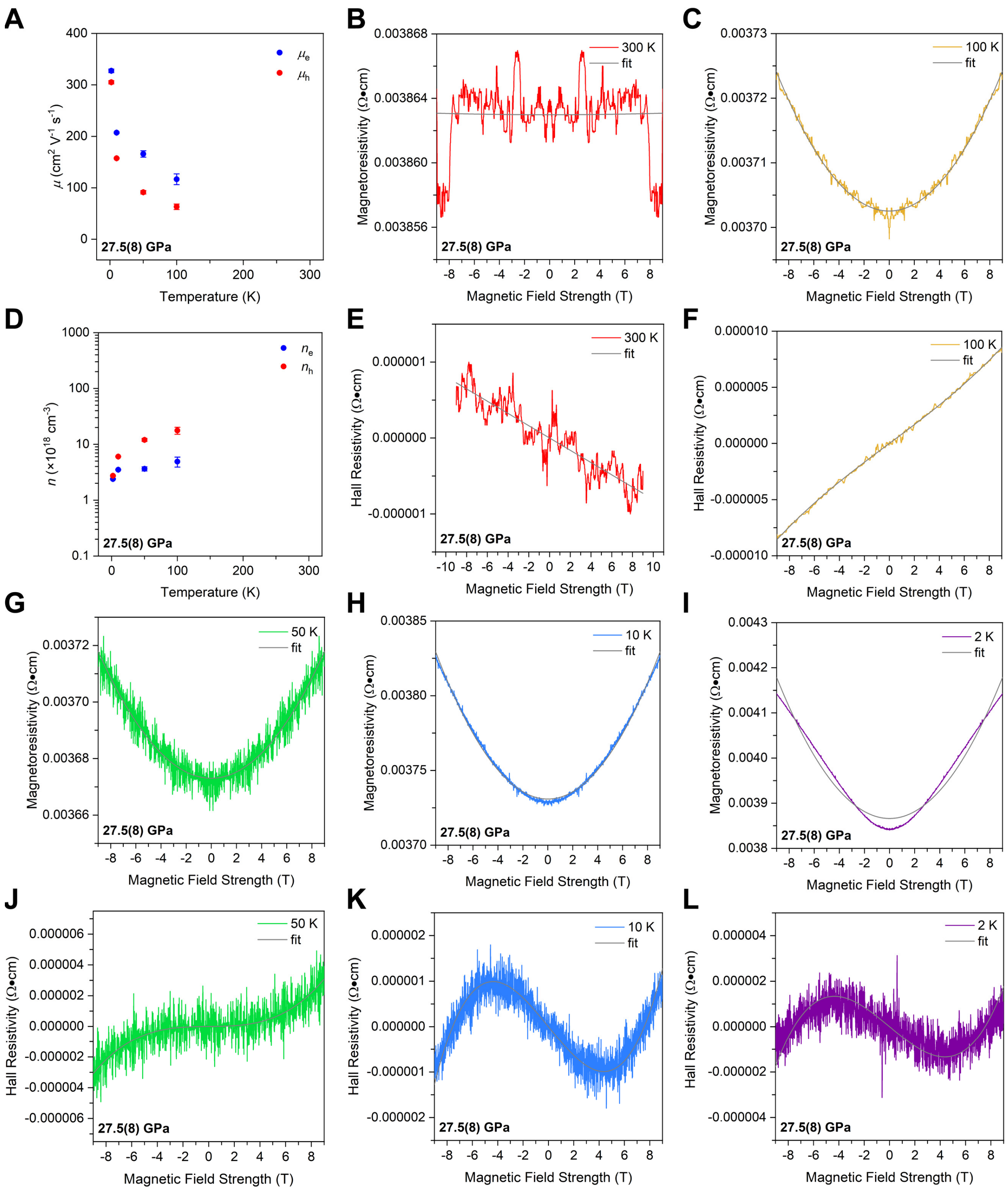

Figure S43. Temperature dependence of (**A**) carrier mobilities (μ) and (**D**) carrier densities (*n*) in **1** at 27.5(8) GPa determined through global fitting of magnetoresistivity and Hall resistivity, calculated from magnetoresistance and Hall effect measurements. Subscripts of e and h correspond to properties of electrons and holes, respectively. (**B-C, G-I**) Magnetoresistivity of **1** at 27.5(8) GPa collected at 300 K, 100 K, 50 K, 10 K, and 2 K, respectively. (**E-F, J-L**) Hall resistivity of **1** at 27.5(8) GPa collected at 300 K, 100 K, 50 K, 10 K, and 2 K, respectively. Gray lines correspond to global fitting of magnetoresistivity and Hall resistivity to a two-carrier model.

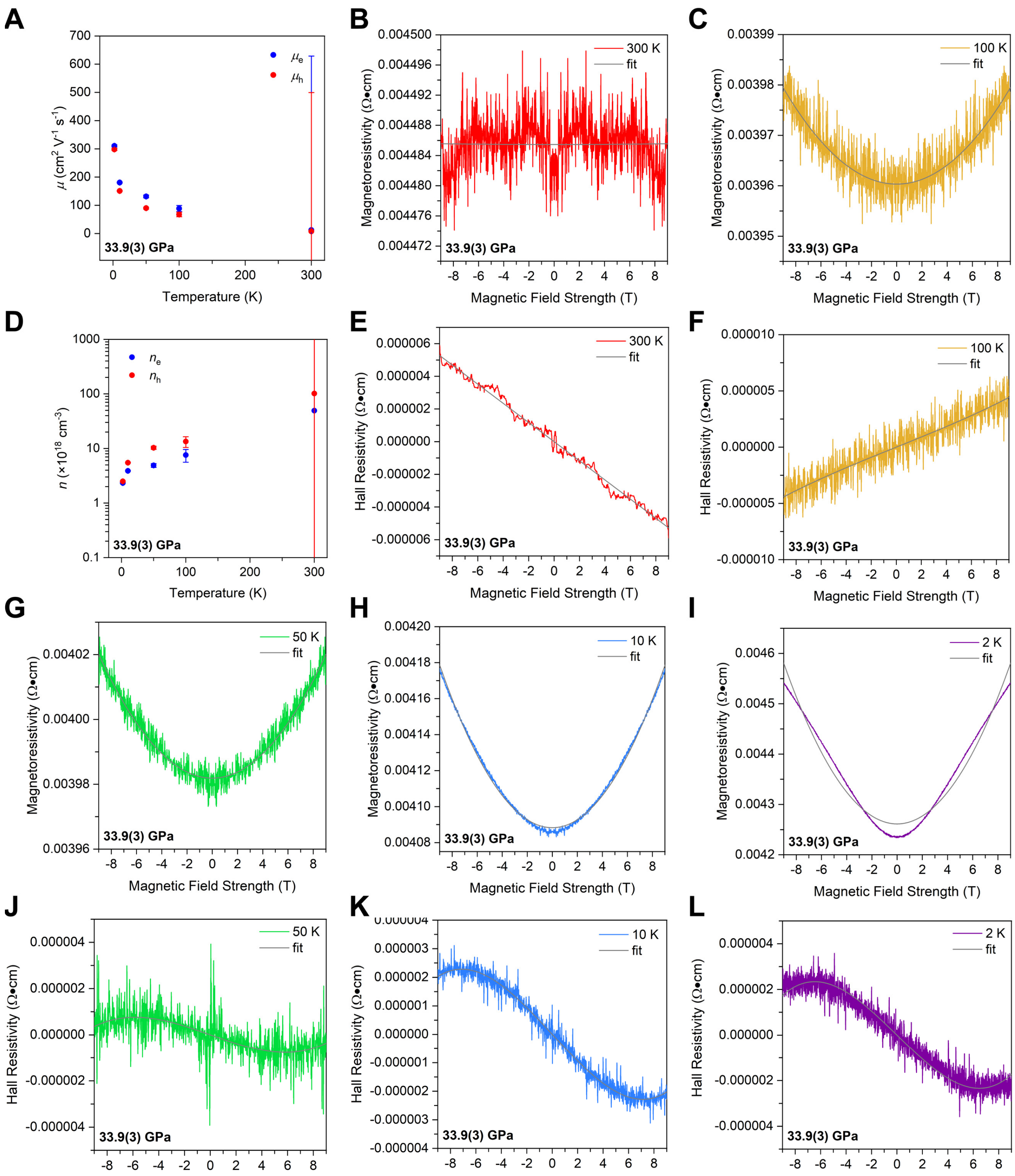

Figure S44. Temperature dependence of (**A**) carrier mobilities (μ) and (**D**) carrier densities (n) in **1** at 33.9(3) GPa determined through global fitting of magnetoresistivity and Hall resistivity, calculated from magnetoresistance and Hall effect measurements. Subscripts of e and h correspond to properties of electrons and holes, respectively. (**B-C, G-I**) Magnetoresistivity of **1** at 33.9(3) GPa collected at 300 K, 100 K, 50 K, 10 K, and 2 K, respectively. (**E-F, J-L**) Hall resistivity of **1** at 33.9(3) GPa collected at 300 K, 100 K, 50 K, 10 K, and 2 K, respectively. Gray lines correspond to global fitting of magnetoresistivity and Hall resistivity to a two-carrier model.

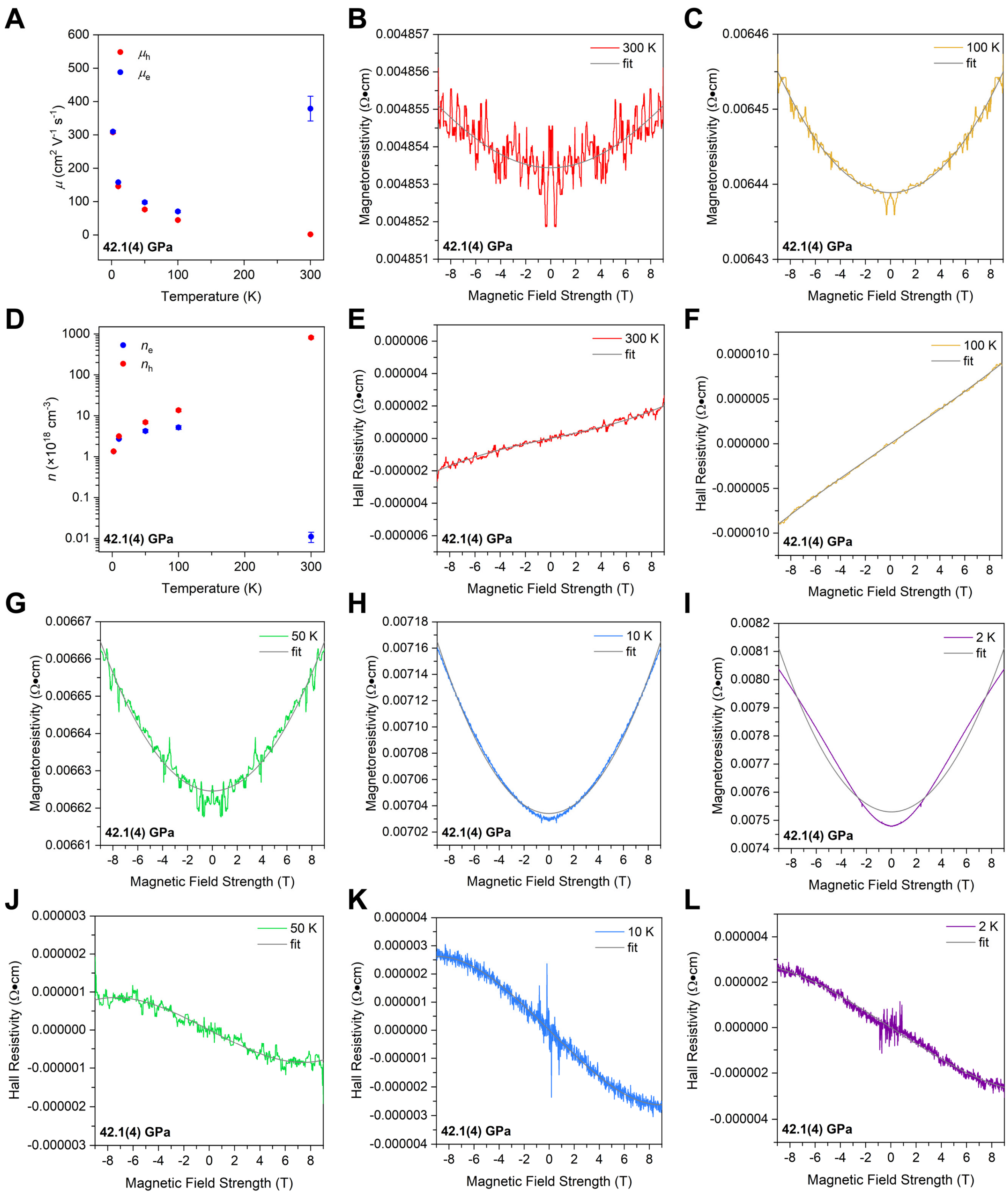

Figure S45. Temperature dependence of (**A**) carrier mobilities (μ) and (**D**) carrier densities (*n*) in **1** at 42.1(4) GPa determined through global fitting of magnetoresistivity and Hall resistivity, calculated from magnetoresistance and Hall effect measurements. Subscripts of e and h correspond to properties of electrons and holes, respectively. (**B-C, G-I**) Magnetoresistivity of **1** at 42.1(4) GPa collected at 300 K, 100 K, 50 K, 10 K, and 2 K, respectively. (**E-F, J-L**) Hall resistivity of **1** at 42.1(4) GPa collected at 300 K, 100 K, 50 K, 10 K, and 2 K, respectively. Gray lines correspond to global fitting of magnetoresistivity and Hall resistivity to a two-carrier model.

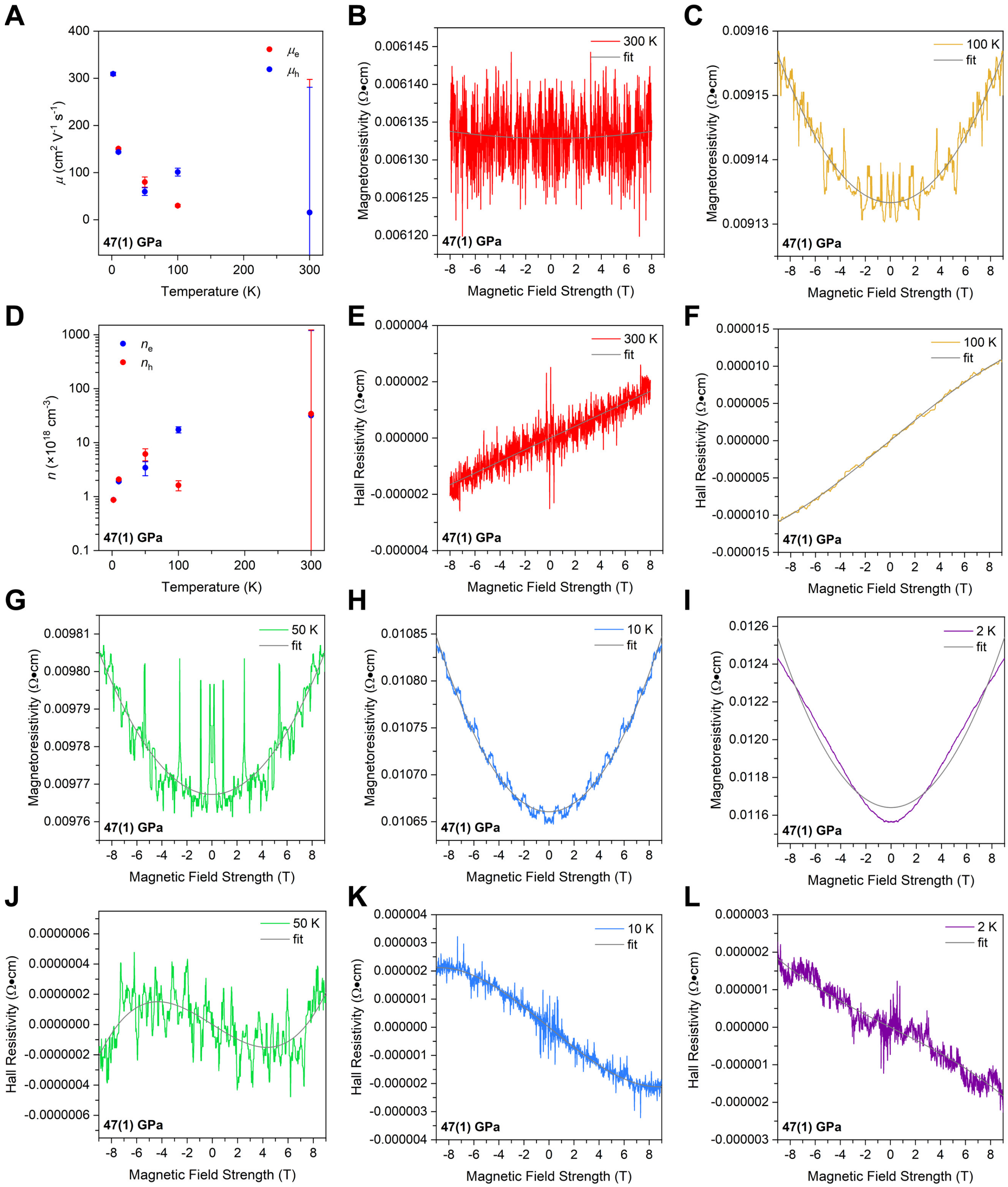

Figure S46. Temperature dependence of (**A**) carrier mobilities (μ) and (**D**) carrier densities (*n*) in **1** at 47(1) GPa determined through global fitting of magnetoresistivity and Hall resistivity, calculated from magnetoresistance and Hall effect measurements. Subscripts of e and h correspond to properties of electrons and holes, respectively. (**B-C, G-I**) Magnetoresistivity of **1** at 47(1) GPa collected at 300 K, 100 K, 50 K, 10 K, and 2 K, respectively. (**E-F, J-L**) Hall resistivity of **1** at 47(1) GPa collected at 300 K, 100 K, 50 K, 10 K, and 2 K, respectively. Gray lines correspond to global fitting of magnetoresistivity and Hall resistivity to a two-carrier model.

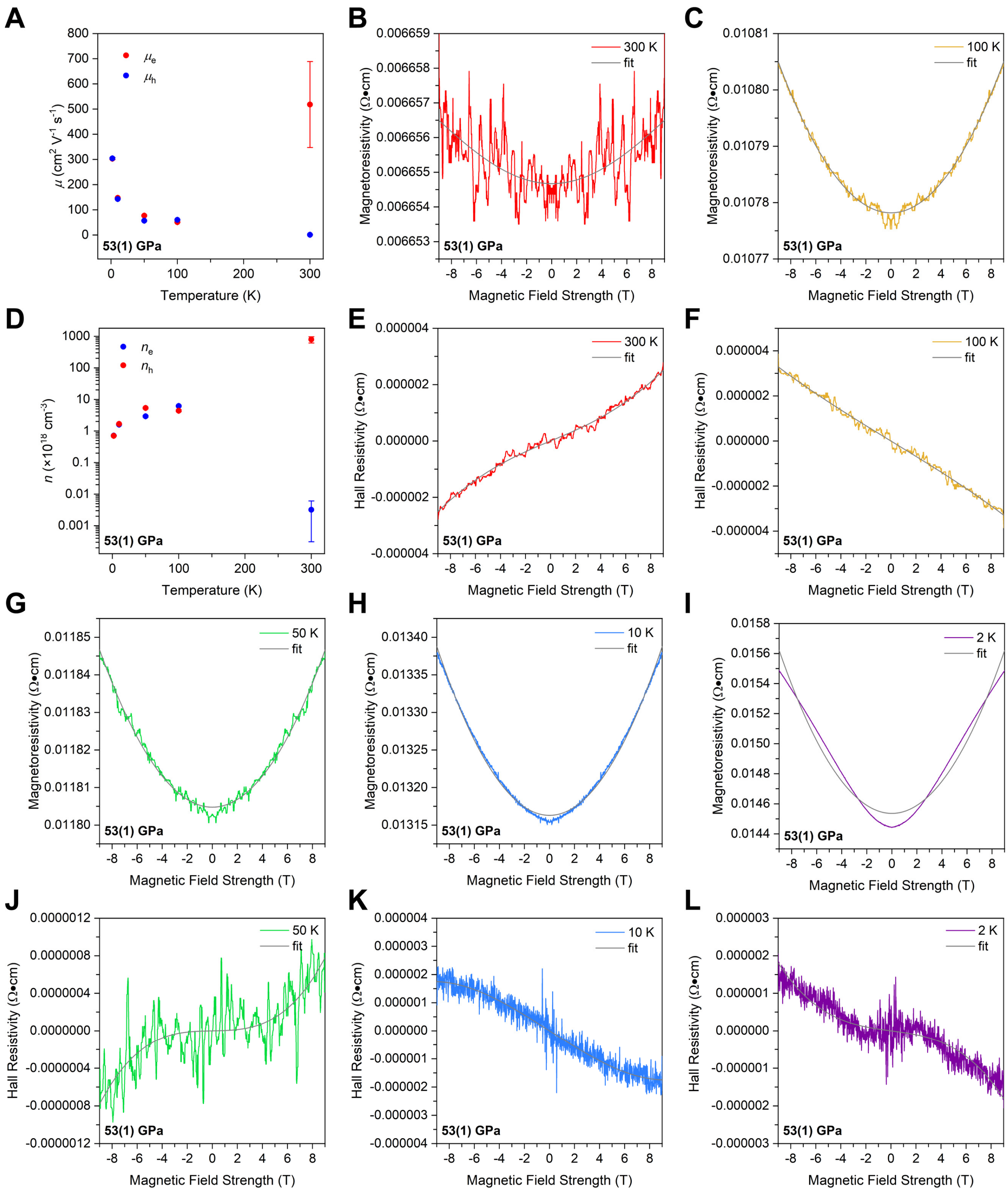

Figure S47. Temperature dependence of (**A**) carrier mobilities (μ) and (**D**) carrier densities (*n*) in **1** at 53(1) GPa determined through global fitting of magnetoresistivity and Hall resistivity, calculated from magnetoresistance and Hall effect measurements. Subscripts of e and h correspond to properties of electrons and holes, respectively. (**B-C, G-I**) Magnetoresistivity of **1** at 53(1) GPa collected at 300 K, 100 K, 50 K, 10 K, and 2 K, respectively. (**E-F, J-L**) Hall resistivity of **1** at 53(1) GPa collected at 300 K, 100 K, 50 K, 10 K, and 2 K, respectively. Gray lines correspond to global fitting of magnetoresistivity and Hall resistivity to a two-carrier model.

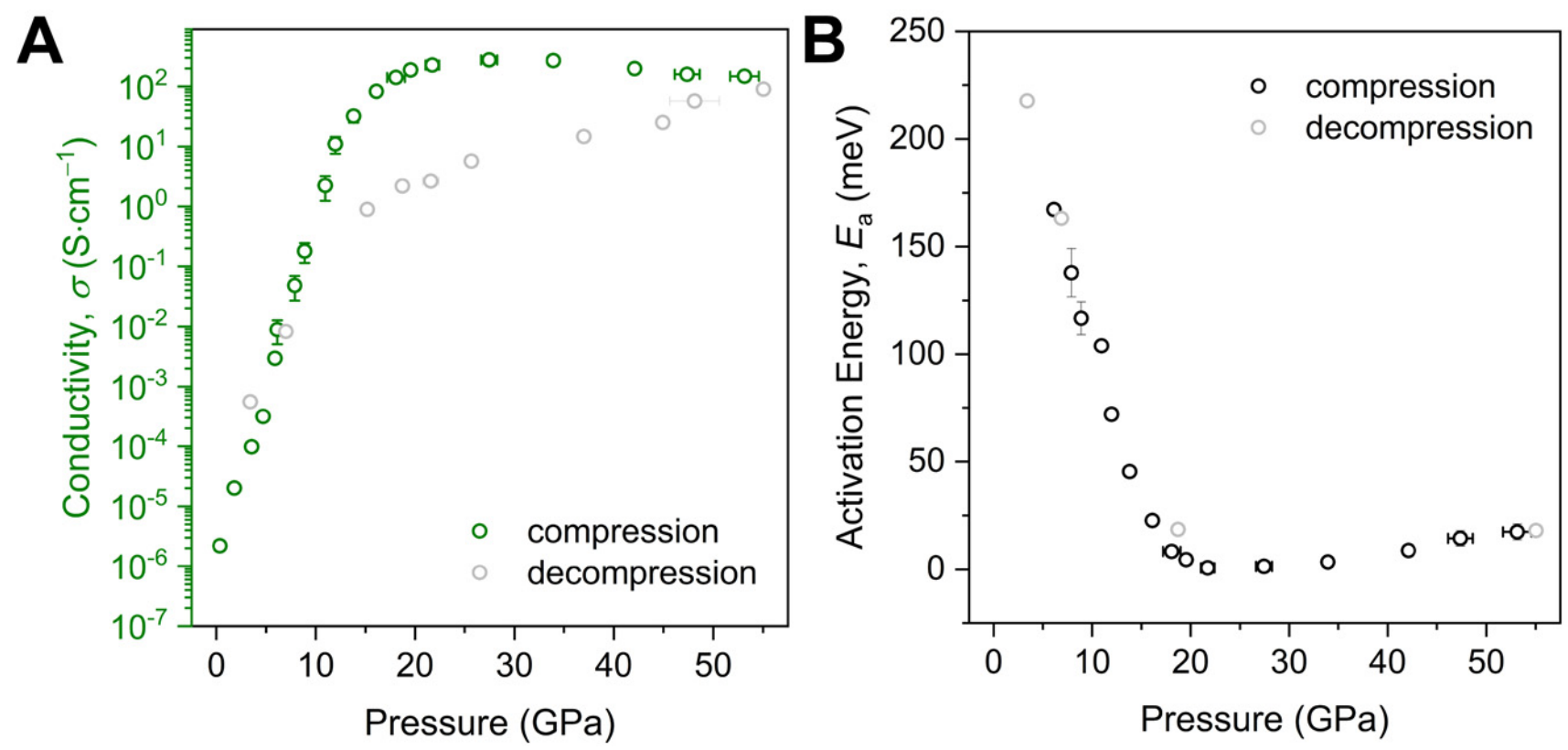

Figure S48. (**A**) Conductivity and (**B**) activation energies of conductivity upon compression (green and black, respectively) and decompression (gray) of **1**.

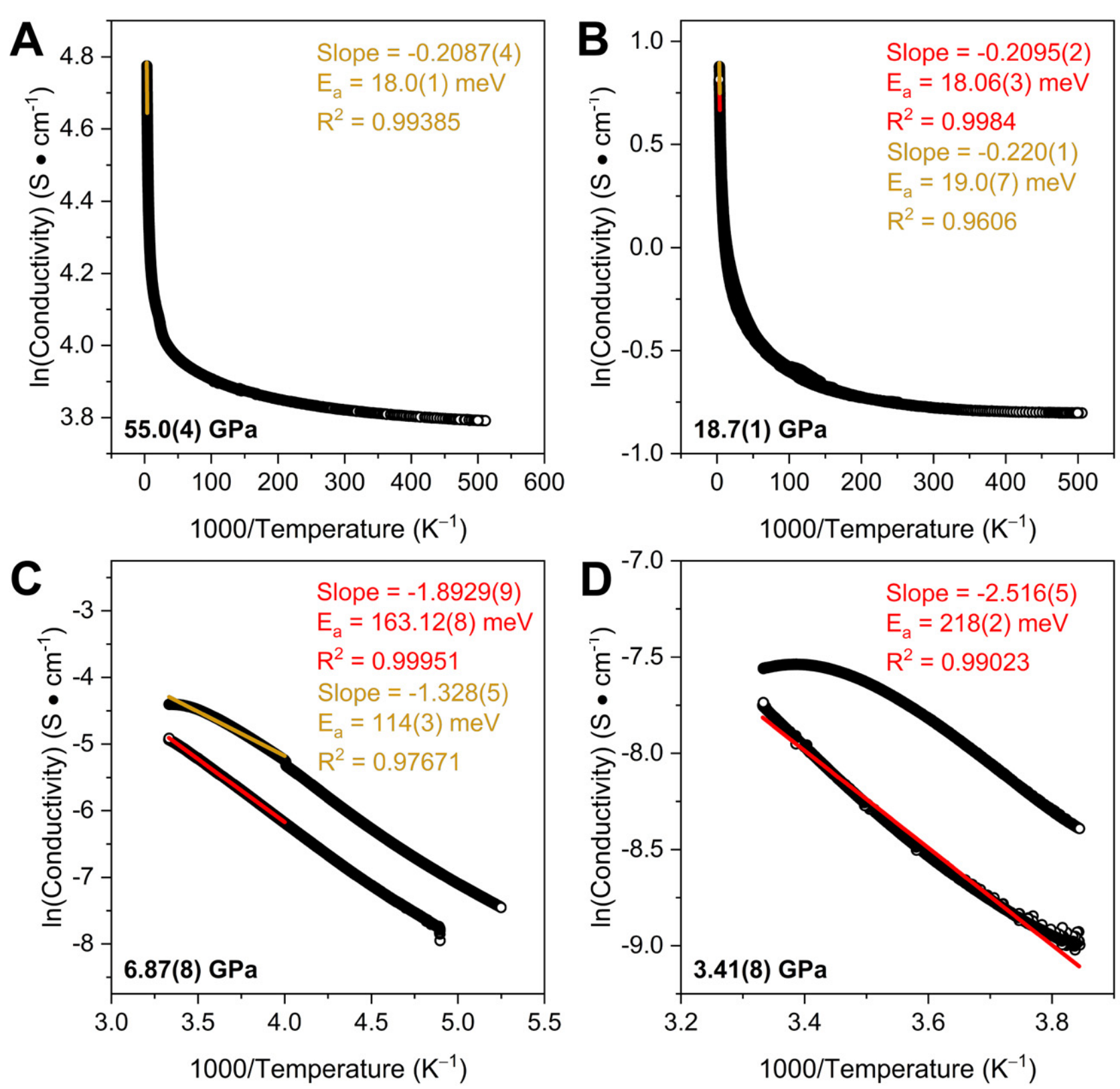

Figure S49. Variable-temperature conductivity of **1** at (**A**) 55.0(4) GPa and upon decompression to (**B**) 18.7(1) GPa, (**C**) 6.87(8) GPa, and (**D**) 3.41(8) GPa with corresponding Arrhenius fits upon cooling (tan) and heating (red).

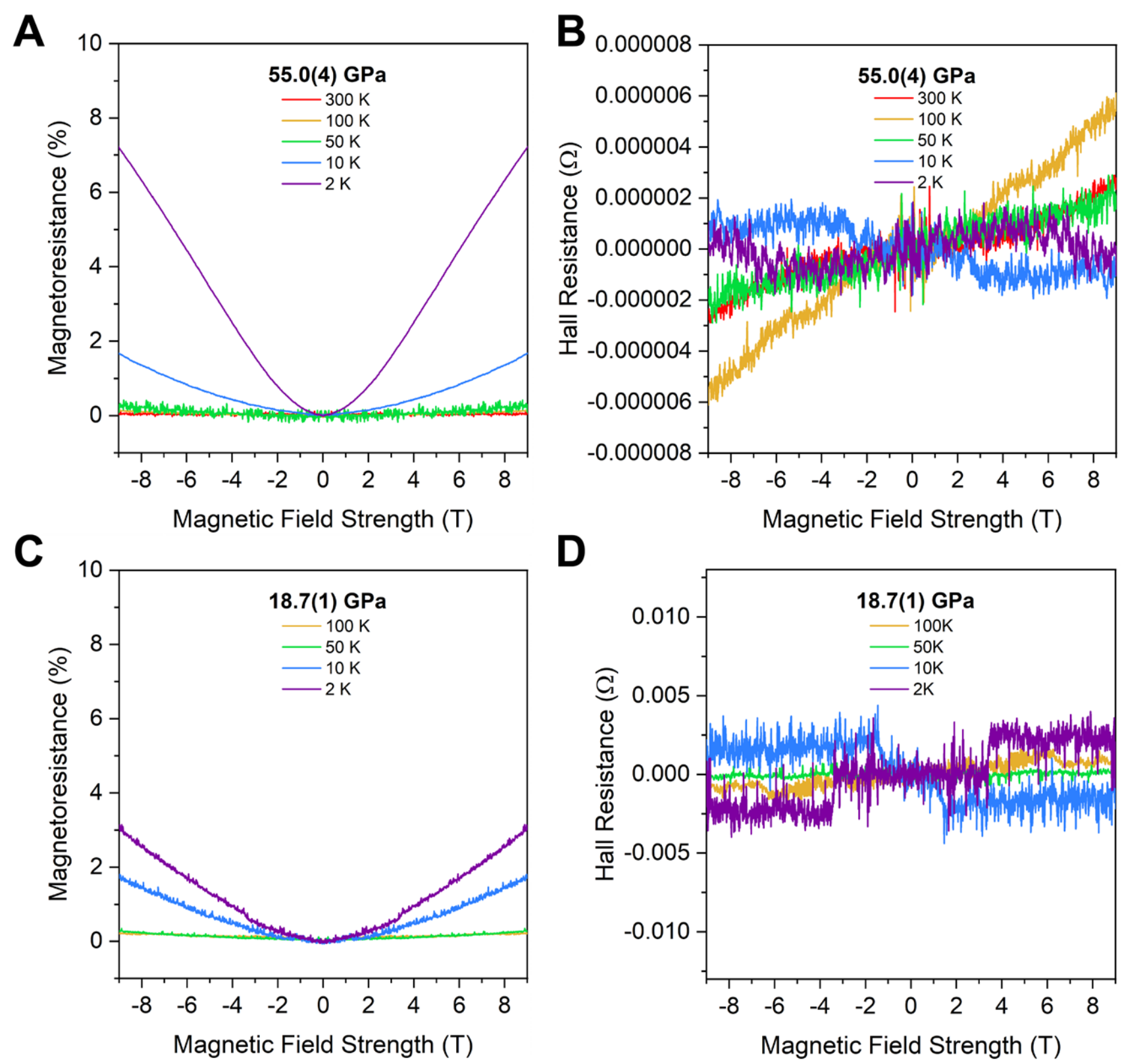

Figure S50. Magnetoresistance (percentage increase) and Hall resistance of **1** (**A-B**) at 55.0(4) GPa and (**C-D**) upon decompression to 18.7(1) GPa. Red, yellow, green, blue, and purple lines correspond to measurements collected at 300 K, 100 K, 50 K, 10 K, and 2 K, respectively.

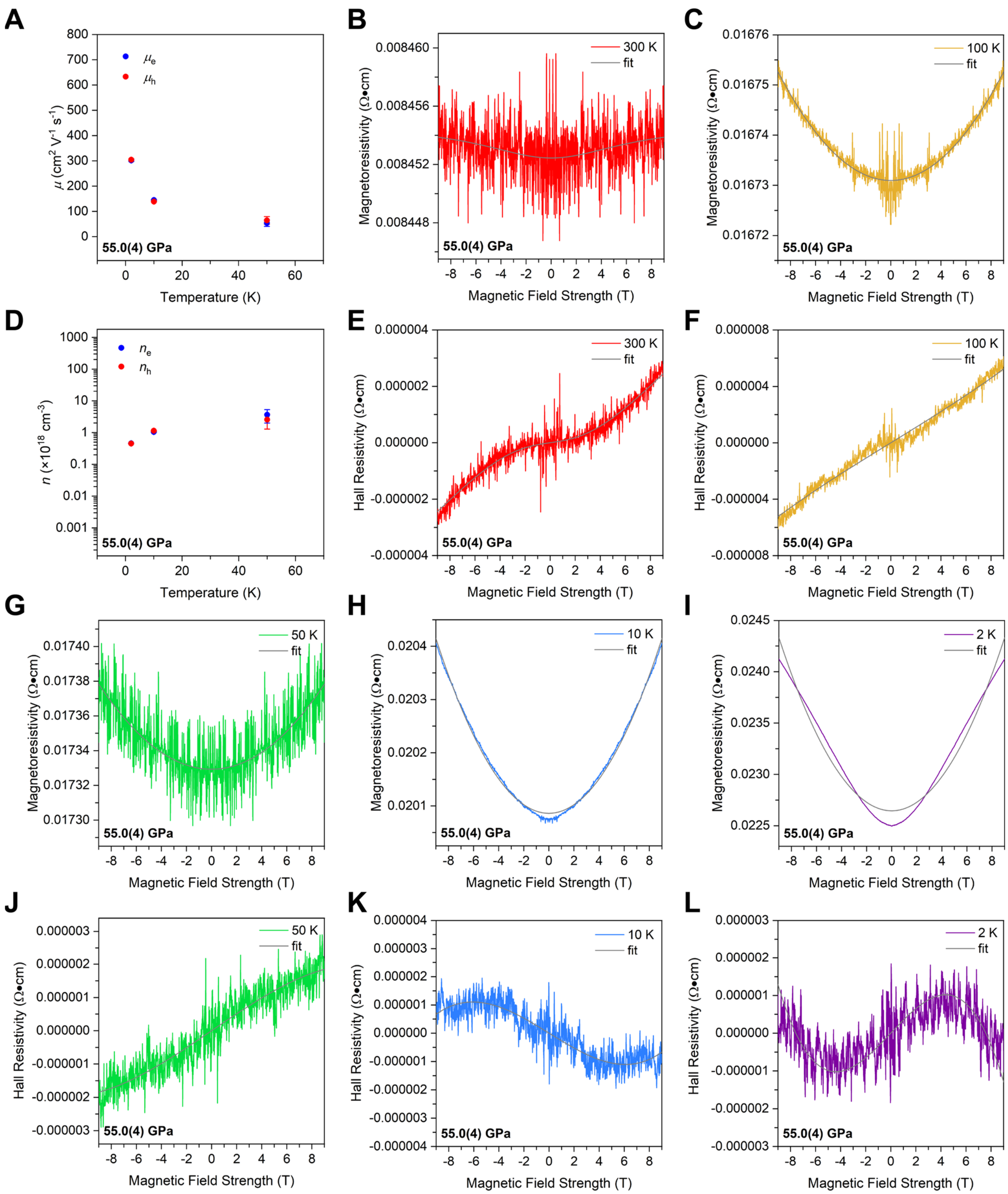

Figure S51. Temperature dependence of (**A**) carrier mobilities (μ) and (**D**) carrier densities (*n*) measured immediately before decompression of **1** at 55.0(4) GPa determined through global fitting of magnetoresistivity and Hall resistivity, calculated from magnetoresistance and Hall effect measurements. Subscripts of e and h correspond to properties of electrons and holes, respectively. (**B-C, G-I**) Magnetoresistivity of **1** at 55.0(4) GPa collected at 300 K, 100 K, 50 K, 10 K, and 2 K, respectively. (**E-F, J-L**) Hall resistivity of **1** at 55.0(4) GPa collected at 300 K, 100 K, 50 K, 10 K, and 2 K, respectively. Gray lines correspond to global fitting of magnetoresistivity and Hall resistivity to a two-carrier model.

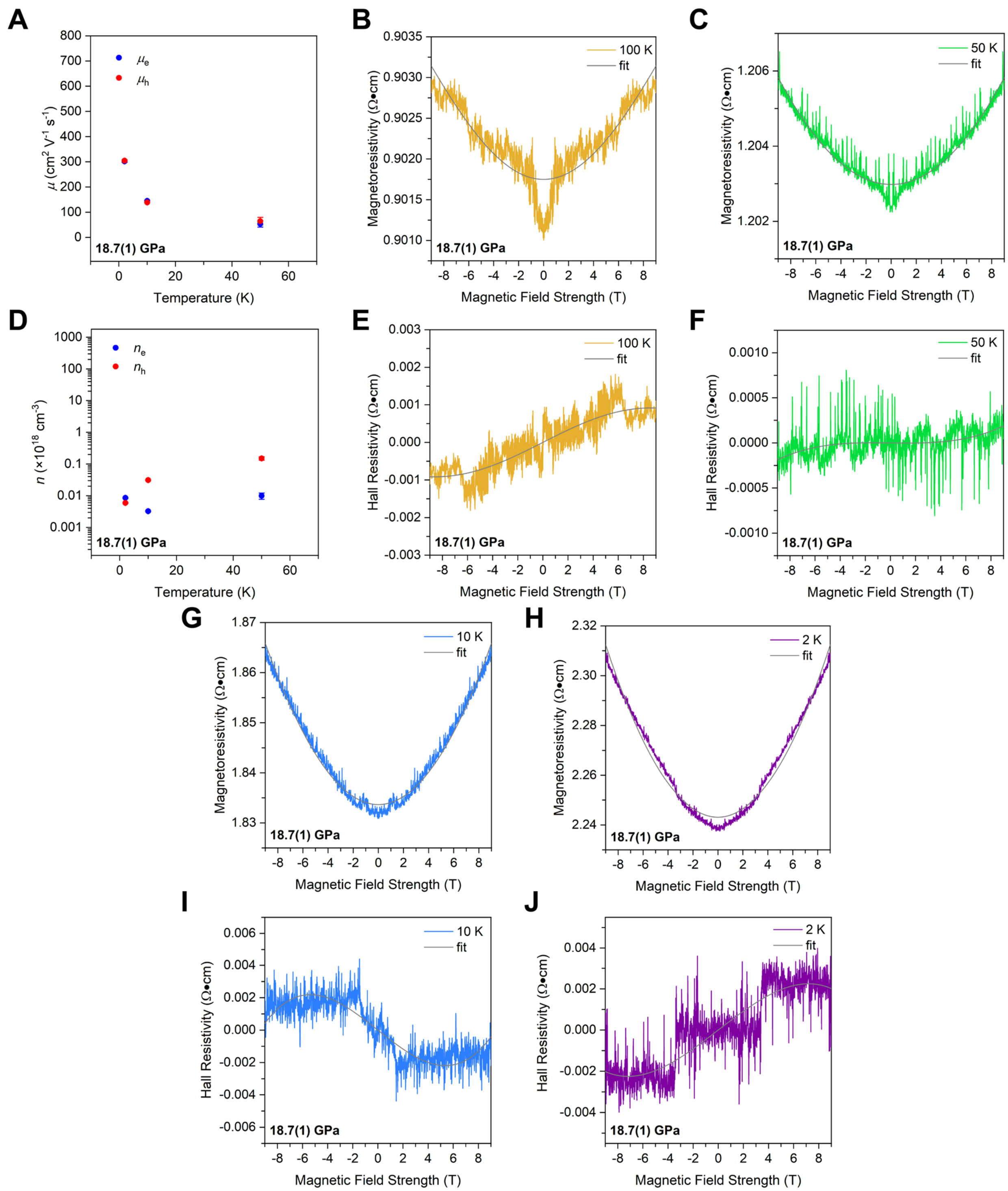

Figure S52. Temperature dependence of (**A**) carrier mobilities (μ) and (**D**) carrier densities (*n*) upon decompression of **1** to 18.7(1) GPa determined through global fitting of magnetoresistivity and Hall resistivity, calculated from magnetoresistance and Hall effect measurements. Subscripts of e and h correspond to properties of electrons and holes, respectively. (**B-C, G-H**) Magnetoresistivity of **1** upon decompression to 18.7(1) GPa collected at 100 K, 50 K, 10 K, and 2 K, respectively. (**E-F, I-J**) Hall resistivity of **1** upon decompression to 18.7(1) GPa collected at 100 K, 50 K, 10 K, and 2 K, respectively. Gray lines correspond to global fitting of magnetoresistivity and Hall resistivity to a two-carrier model.

Supplementary Tables

Table S1. Lattice parameters and unit-cell volumes from Pawley refinement of diffraction patterns for the α phase of **1**.

**Pressure (GPa)**	***a*** **(Å)**	***c*** **(Å)**	**Volume (Å)**
0.565(8)	20.816(3)	21.14(1)	9162(5)
0.660(0)	20.770(2)	21.082(6)	9094(2)
0.800(0)	20.70(1)	21.042(8)	9020(9)
0.930(0)	20.659(4)	20.994(6)	8960(1)
1.055(8)	20.607(6)	20.937(7)	8891(1)
1.275(8)	20.54(1)	20.85(1)	8780(16)
1.495(8)	20.46(1)	20.79(2)	8700(15)
1.61(1)	20.3556(3)	20.6490(1)	8556.0(1)
1.635(8)	20.414(8)	20.74(1)	8644(7)
1.85(1)	20.344(7)	20.67(1)	8556(11)

Table S2. Lattice parameters and unit-cell volumes from Pawley refinement of diffraction patterns for the β phase of **1**.

**Pressure (GPa)**	***a*** **(Å)**	***b*** **(Å)**	***c*** **(Å)**	**Volume (Å)**
2.15(1)	20.30(2)	20.12(3)	20.551(2)	8396(20)
2.40(1)	20.252(5)	20.080(8)	20.489(7)	8332(5)
2.62(3)	20.1744(5)	19.9591(4)	20.4228(8)	8223.48(5)
2.67(0)	20.120(7)	19.899(4)	20.373(4)	8156(6)
3.17(2)	20.09(1)	19.884(6)	20.326(4)	8119(9)
3.30(2)	20.000(2)	19.693(7)	20.281(3)	7988(2)
4.03(2)	19.85(1)	19.67(1)	20.07(2)	7837(14)
4.09(0)	19.868(2)	19.54(1)	20.126(9)	7814(9)
4.80(1)	19.74(1)	19.441(6)	19.99(2)	7673(14)
4.91(2)	19.697(6)	19.515(7)	19.920(9)	7657(5)
5.33(1)	19.68(3)	19.335(7)	19.94(2)	7589(16)
5.94(5)	19.58(1)	19.35(1)	19.80(2)	7500(11)
6.33(6)	19.52(2)	19.260(6)	19.78(1)	7435(14)
7.23(6)	19.41(1)	19.143(3)	19.677(1)	7313(4)

Table S3. Lattice parameters and unit-cell volumes from Pawley refinement of diffraction patterns for the γ phase of **1**.

**Pressure (GPa)**	***a*** **(Å)**	**Volume (Å)**
15.77(5)	18.7616(1)	6604.1(1)
16.51(6)	18.7162(3)	6556.24(3)
17.25(5)	18.67878(0)	6516.9697(3)
18.02(6)	18.63929(0)	6475.7177(3)
18.63(6)	18.6033(5)	6438.25(6)
19.57(9)	18.54446(7)	6377.392(6)
20.1(4)	18.452(5)	6282(5)
20.67(8)	18.51044(0)	6342.350(3)
22.3(1)	18.46496(3)	6295.711(4)
24.1(1)	18.42254(0)	6252.428(2)
26.71(6)	18.3395(1)	6168.2(1)
26.73(8)	18.1978(2)	6026.4(2)
28.72(6)	18.2467(3)	6075.1(3)
29.71(5)	18.0777(2)	5907.9(2)
31.3(6)	18.1254(2)	5954.7(2)
33.1(1)	17.9582(1)	5791.5(1)
34.7(1)	18.0397(5)	5870.7(5)
38.37(9)	17.8120(2)	5651.1(2)
39.0(3)	17.90457(0)	5739.733(3)
42.9(2)	17.6811(2)	5527.5(2)
43.2(2)	17.79123(3)	5631.416(1)
49.1(1)	17.5194(4)	5377.2(4)
52.9(2)	17.4210(4)	5287.1(4)
56.5(3)	17.3274(2)	5202.4(2)

Table S4. Absolute values of tabulated effective masses obtained from our DFT-HSE calculations of the band structures of **1** at 0 GPa, 8 GPa, 16 GPa, 20 GPa, and 24 GPa at the *k* points corresponding to the valence band maximum (VBM), calculated from the second derivatives of the band curvature with respect to 0.025 1/Bohr *k*-grid step-size sampling around each point. These values are multiples of the electron mass at rest (m_0). Some pressures have degenerate *k*-point energies for the VBM.

Pressure (GPa)	X	P	N	Γ	M
0		4.3*		2.0	
8		1.5		0.26	1.6
16					0.20
20					0.15
24					0.24

* = coarse *k*-point sampling from flat bands led to an outlier for one eigenvector, so this is the absolute average of the two remaining eigenvectors.

Table S5. Absolute values of tabulated effective masses obtained from our DFT-HSE calculations of the band structures of **1** at 0 GPa, 8 GPa, 16 GPa, 20 GPa, and 24 GPa at the *k* points corresponding to the conduction band minimum (CBM), calculated from the second derivatives of the band curvature with respect to 0.025 1/Bohr *k*-grid step-size sampling around each point. These values are multiples of the electron mass at rest (m_0). Some pressures have degenerate *k*-point energies for the CBM.

Pressure (GPa)	X	P	N	Γ	M
0	0.26		0.29		
8	0.082		0.094		
16	0.079		0.095		
20		0.07			
24		0.16			

Table S6. Eigenvectors used to tabulate the absolute values of the effective masses, obtained from our DFT-HSE calculations and shown in Table S4, of the band structures of **1** at 0 GPa, 8 GPa, 16 GPa, 20 GPa, and 24 GPa at the *k* points corresponding to the VBM. These values were determined through diagonalization of the 3 × 3 matrix of the second derivative of the band curvature. They are multiples of the electron mass at rest (m_0).

Pressure (GPa)	X	P	N	Γ	M
0		−3.045		2.749	
		−5.629		1.684	
		−32.171		1.639	
8		−0.376		0.313	−0.376
		2.524		0.243	2.441
		1.661		0.227	1.888
16					−0.117
					0.242
					0.241
20					−0.035
					0.217
					0.198
24					−0.029
					0.463
					0.236

Table S7. Eigenvectors used to tabulate the absolute values of the effective masses, obtained from our DFT-HSE calculations and shown in Table S5, of the band structures of **1** at 0 GPa, 8 GPa, 16 GPa, 20 GPa, and 24 GPa at the *k* points corresponding to the CBM. These values were determined through diagonalization of the 3 × 3 matrix of the second derivative of the band curvature. They are multiples of the electron mass at rest (m_0).

Pressure (GPa)	X	P	N	Γ	M
0	−0.181		−0.187		
	−0.186		−0.193		
	−0.406		−0.476		
8	−0.065		−0.061		
	−0.069		−0.065		
	−0.111		−0.157		
16	−0.064		−0.053		
	−0.07		−0.062		
	−0.102		−0.169		
20		−0.042			
		−0.059			
		−0.109			
24		−0.048			
		−0.065			
		−0.367			

Table S8. Absolute values of tabulated effective masses obtained from our DFT-HSE calculations at the VBM and CBM of the band structures of **1** at 8 GPa, 16 GPa, and 20 GPa, calculated from the second derivatives of the band curvature with respect to different k-grid sampling step sizes around each point: 0.015, 0.025, and 0.035 (in units of 1/Bohr). These values are multiples of the electron mass at rest (m_0). Effective masses at the CBM were calculated at N for 8 GPa and 16 GPa, and P at 20 GPa. Effective masses at the VBM were calculated at M for each pressure.

	**8 GPa**			**16 GPa**			**20 GPa**		
**Step Size (1/Bohr)**	0.015	0.025	0.035	0.015	0.025	0.035	0.015	0.025	0.035
**CBM**	0.033	0.094	0.192	0.033	0.095	0.196	0.025	0.070	0.141
**VBM**	0.696	1.568	2.714	0.071	0.200	0.411	0.053	0.150	0.467

Table S9. Tabulated ratios of thermal (E_a) and optical (E_{op}) IVCT barrier ratios of **1** and values of the difference in energy of potential well minima (E_0) at each pressure according to Hush.[21]
* = value obtained from ref. 1.

**Pressure (GPa)**	**E_{op} obtainment**	**E_{op} (eV)**	**E_a (eV)**	**$E_a : E_{op}$**	**E_0 (eV)**
0	hν$_{max}$	1.43(8)*	0.600(5)*	1:2.4	0.58
0	onset	0.85(3)	0.600(5)*	1:1.4	0.55
6	onset	0.265(1)	0.167(3)	1:1.6	0.16

Supplementary Discussion

Supplementary Discussion 1. Prior Studies of Mixed-Valence Au Perovskites

IVCT transition mechanism

Intervalence charge transfer (IVCT) transitions in materials with mixed-valence metal sites often occur between the highest occupied molecular orbital (HOMO) of the smaller valence state of a metal (e.g., Au^+ in Au^+–Au^{3+} mixed-valence), and the lowest unoccupied molecular orbital (LUMO) of the larger valence state of a metal. Because this HOMO to LUMO energy gap typically corresponds to the lowest energy transition, it often dictates the bandgap of mixed-valence halide perovskites.[7] However, Kojima and Kitagawa proposed that the lowest energy optical transition of $Cs_2Au^IAu^{III}X_6$ ($X = Cl^-, Br^-, I^-$), which they attribute to IVCT, is a HOMO – 1 to LUMO transition. Using group theory analysis for orbital assignment, they determine that the HOMO (Au^+ d_{z^2}) and LUMO (Au^{3+} $d_{x^2-y^2}$) are orthogonal, precluding inner-sphere IVCT (Figure S1).[8] Instead, they determine that the next smallest energy gap for a transition with significant orbital overlap is from the HOMO – 1 (Au^+ $d_{x^2-y^2}$) to the LUMO. Polarized reflectance showed that this transition exhibited polarization in the *ab* plane. Since their predicted HOMO – 1 to LUMO transition only exhibits orbital overlap in the *ab* plane, Kojima and Kitagawa proposed that the lowest-energy IVCT transition is from Au^+ $d_{x^2-y^2}$ to Au^{3+} $d_{x^2-y^2}$ through a σ-bonding Cl p orbital.

High-pressure studies

High-pressure studies have been performed on the Au-halide perovskites: $Cs_2Au^IAu^{III}X_6$ (X = Cl, Br, I), to elucidate their mixed-valence class and emergent properties at high pressures. We refer the reader to our review[7] detailing the high-pressure evolution of these structures, which is summarized here. Mechanical force has been shown to compete against a Jahn-Teller distortion in related Cu^{2+}–halide perovskites,[9] and this effect has been reported to varying degrees for each composition of $Cs_2Au^IAu^{III}X_6$.

In $Cs_2Au^IAu^{III}Cl_6$, mechanical force drives a structural transition from tetragonal to cubic symmetry, characterized by single-crystal X-ray diffraction at 15 GPa and room temperature.[10] Equivalent octahedral (O_h) coordination for all Au sites in the single-crystal structural solution suggests complete suppression of the second-order/pseudo Jahn-Teller distortion of the Au^ICl_6 and $Au^{III}Cl_6$ polyhedra. The disappearance of Raman modes at 12 GPa, which are present in the tetragonal structure but are disallowed in the cubic structure of $Cs_2Au^IAu^{III}Cl_6$, was also attributed to the transition to the cubic structure,[11–13] though increased conductivity from compression can also reduce the intensity of Raman modes. As the Au sites become equivalent in this higher-symmetry coordination, the IVCT energy should decrease to zero, resulting in a Robin-Day Class II to Class III (semiconductor to metal) transition.[14] Indeed, the activation energy of conductivity drops below 20 meV by 6 GPa, and a trend of increasing conductivity with decreasing temperature is observed between 7 GPa and 11 GPa, which is expected for metallic transport.[15,16] Although optical absorption onsets have been shown to fall below mid-infrared energies prior to the phase transition,[11] no far-infrared absorption or Drude features in reflectivity data have been reported to corroborate a metallic state. Additionally, there has never been experimental confirmation of a single-valent Au^{2+} state reported for this composition to our knowledge.

Interestingly, $Cs_2Au^IAu^{III}Br_6$ has been reported to undergo different structural transitions under pressure than its Cl analog. A tetragonal-to-tetragonal (*I*4/*mmm* to *P*4/*mmm*) symmetry transition at 8.1 GPa has been reported by Ohishi and Takata et al. through Rietveld refinement and Maximum Entropy Method refinement of high-pressure powder X-ray diffraction.[17] This transition does not completely eliminate second-order/pseudo Jahn-Teller distortions, but the distortion changes at the transition as the Au sites become equivalent. Movement of each unique Br atom to the midpoint between two adjacent Au atoms results in axial elongation for each Au site, with all of the long axes parallel to one another in the structure. Similar to $Cs_2Au^IAu^{III}Cl_6$, increasing conductivity with decreasing temperature has been observed at 6.8

GPa in $Cs_2Au^IAu^{III}Br_6$, which is expected for a metallic state.[18] Von Rohr *et al.* attribute this to a tetragonal (*I*4/*mmm*) to cubic ($Pm\bar{3}m$) structural phase transition through high-pressure structural relaxations using density functional theory and Rietveld refinement of high-pressure powder X-ray diffraction. However, the *c* axis is >3 Å longer than the *a* axis at all pressures for their Rietveld refinements and DFT calculations, which is inconsistent with cubic symmetry. The data agree with the conclusion of Ohishi and Takata et al., as described above, of a transition to higher tetragonal symmetry (*I*4/*mmm* to *P*4/*mmm*). The structural refinements and metallic temperature-dependence of the resistivity are attributed to complete electronic delocalization in the higher-symmetry tetragonal phase, where each Au is expected to have an oxidation state of Au^{2+},[18] though this single-valence state has never been directly observed for this composition. Interestingly, at pressures higher than the transition to *P*4/*mmm* symmetry, decreases in conductivity at room temperature and the return to a trend of decreasing conductivity with decreased temperature, expected for a semiconductor, are observed. However, the origin of this transition is unknown.

High-pressure structural studies of $Cs_2Au^IAu^{III}I_6$ also differ from the Cl- and Br-analogs, and several reports of the I analog conflict with one another. In one report, a high-pressure tetragonal-to-orthorhombic structural transition is reported at 8.9 GPa, with two possible space groups for the orthorhombic phase (*Ibmm* and *Immm*). Both retain second-order Jahn-Teller distortion of the Au sites, but only one (*Ibmm*) results in equivalent coordination of the Au sites.[19] The ^{197}Au Mössbauer spectra of the I analog contain doublets assigned to Au^+ and Au^{3+} at pressures prior to the phase transition, but these merge to a single doublet, intermediate in energy, at 12.5 GPa.[20] This is attributed to the Au^{2+} single-valence state, which suggests a Class II to Class III mixed-valence transition at this pressure, and is consistent with the *Ibmm* space group for the high-pressure phase. However, Drude features in far-infrared reflectivity suggest metallic character at pressures as low as 5.6 GPa,[21] so the pressures of the structural phase transitions and the property changes differ significantly. This may be attributed to non-hydrostatic conditions in diamond anvil cells, which may cause differences in measured and actual pressures, and discrepancies in average and local symmetries with pressure. However, similar to the Br analog, bandgap reopening in far-infrared absorption is reported at pressures higher than the orthorhombic transition with an unknown cause.[21] Additionally, other reports have very different findings. One reports a tetragonal-to-tetragonal (*I*4/*mmm* to *P*4/*mmm*) symmetry transition, like in $Cs_2Au^IAu^{III}Br_6$, at 5.5 GPa.[22] However, it is attributed to Au-site disorder or localized Au^{2+} sites, rather than a delocalized metallic state. They also suggest decomposition to an irreversible amorphous phase at 12 GPa. Further, they propose that their measured activation energies of conductivity at 17.5 GPa, which drop to within error of zero, could be from the presence of elemental iodine from this decomposition, which is metallic at these pressures. Further magnetic studies would be necessary to probe the existence of Au^{2+} sites, and careful structural and conductivity studies (with careful consideration of applied current to rule out redox reactions of the structure during measurements) would need to be performed with identical pressure transmitting media to abate these inconsistences.

Supplementary Discussion 2. PXRD phase assignments of $Cs_4Au^{II}Au^{III}_2Cl_{12}$

We conducted high-pressure powder X-ray diffraction (PXRD) and Raman spectroscopy measurements of **1** to probe its structural response to compression. The ambient-pressure structure of **1** has tetragonal symmetry in the $I4_1cd$ space group. The tetragonal symmetry (α phase) of **1** persists up to 2.2 GPa, when a transition to orthorhombic symmetry (β phase) occurs (Figures S3A,D). The emergence of high-angle shoulders on peaks that exhibit tetragonal splitting at ambient pressure (e.g., the (400)/(004) diffraction peaks) indicate shortening of the *b* axis and an orthorhombic transition at 2.2 GPa (Figure S3B).

Upon further compression, a sluggish second phase transition begins at 8.7 GPa. By 15 GPa, peaks with orthorhombic splitting (i.e., splitting of the (*h*00)/(0*k*0)/(00*l*) diffraction peaks corresponding to the three unique axes in the orthorhombic phase) converge to one peak, suggesting a transition to cubic symmetry (γ phase, Figure S3B). Peaks corresponding to this γ phase are present in diffraction patterns up to 57 GPa, albeit with increased broadening at the highest pressures (Figure S3A).

These trends of moving to lower symmetry (β phase) and then higher symmetry (γ phase) are also consistent with the pressure-dependence of the diffraction peak widths (full-width at half-maximum). Peaks containing reflections from diffraction planes along all three unit-cell axes exhibit broadening at the β phase (corresponding to the separation of lattice parameter values) and subsequent narrowing (due to the convergence of lattice parameter values) at the γ phase (Figure S3C). Though peak broadening can often be attributed to non-hydrostaticity of the sample chamber, Ne is a suitable hydrostatic pressure-transmitting medium at 2.2 GPa where the broadening is observed, so we attribute the peak broadening to a true decrease in symmetry. Additionally, the subsequent peak narrowing at still higher pressures could only indicate a phase change to higher symmetry at the γ phase.

Using GSAS-II indexing software[23], we indexed the α, β, and γ phases to $I4_1cd$, $Iba2$, and $Ia\overline{3}d$ space groups, respectively (Figure S4). We used Pawley refinement to fit the diffraction patterns and extract unit-cell lattice parameters and volumes with pressure (Figure 1B, Figure S5, Tables S1-3). Rietveld refinement was not used for the α phase due to limited reliability of refinement. DAC powder diffraction data often exhibits low intensities, and our synthesis method requires hand-grinding of single crystals for powder data such that the diffraction rings were not completely smooth from inhomogeneous grain sizes. Both of these issues prevent adequate Rietveld refinement, since it requires smooth diffraction rings and knowledge of the sample texture for appropriate modelling. Therefore, low-pressure patterns were phase-indexed and Pawley refined like the high-pressure phases. This level of refinement is more reliable for diffraction patterns with unknown or inhomogeneous texture because it fits the intensities arbitrarily and only takes into consideration the indexed space group and lattice parameters to generate expected reflection positions.

Though each of these phases exhibit suitable Pawley refinement statistics, we note that definitive assignment of a truly cubic phase at the γ phase transition is impossible with this level of refinement. A slight tetragonal distortion is also possible, though diffraction patterns are fit equally well between tetragonal and cubic symmetry. Indeed, calculations suggest that this phase would be better represented in a tetragonal ($I4_1cd$) space group with a very slight tetragonal distortion, as do the optical and electronic transport energy barriers measured at the transition, as discussed in the main text.

Supplementary Discussion 3. Raman and Infrared absorption analysis of $Cs_4Au^{II}Au^{III}_2Cl_{12}$

The unit-cell volume of **1** is too large to perform structural relaxations on the entire unit cell, so calculation of predicted phonon modes of **1** was not possible. Definitive assignment of Raman and infrared absorption modes could not be done as a result. Instead, we take a qualitative approach to evaluating these modes with respect to relative lattice symmetry. Generally, a high-symmetry structure will have fewer allowed Raman and infrared-active modes than a low-symmetry structure.

This behavior is readily observed in the Raman spectra of **1** upon compression (Figure S6). Measurements collected using 638 nm and 785 nm line excitation exhibit features at the same energies, albeit their relative intensities differ. Up to 1.4 GPa, **1** appears to exhibit three distinct modes between 280 cm^{-1} and 375 cm^{-1}. At 2.3 GPa, the number of observed modes dramatically increases to a minimum of 7 modes with significant overlap, indicating a transition to lower symmetry as is consistent with an orthorhombic phase transition. This trend is emulated in far-infrared absorption, where the number of modes increases between 1.9 GPa and 3.4 GPa (Figure S7).

In the Raman spectra, it is notable that *ca.* 4 lower energy modes below 100 cm^{-1} also appear at the orthorhombic phase transition, but are not observed up to 1.4 GPa. A significant blue shift of Raman modes to higher energy with pressure likely caused these modes to be measurable at 2.3 GPa. If the modes are present below this pressure, they likely sit below the measurable energy range of our detector. The number of modes decreases between 8.8 GPa and 16 GPa, at which point a single mode is measured near 330 cm^{-1}

and two lower energy modes are observed near 100 cm^{-1}, consistent with a transition to a higher symmetry (cubic) phase.

No further phase transition is observed via powder diffraction, but a low-energy shoulder begins to appear on the mode centered near 330 cm^{-1} above ca. 21 GPa, after which the peaks begin to broaden and disappear. More lower energy modes begin to appear and increase in intensity as well after 16 GPa, then broaden and disappear with the higher energy peaks. This indicates the introduction of a local distortion that occurs with further pressure increase above 21 GPa. Since Raman spectroscopy is a much more local probe whereas powder diffraction looks at average unit-cell symmetry, this distortion may be averaged within the confines of the unit cell. This structural distortion is the likely cause of optoelectronic property changes above this pressure, discussed in sections III, VII, and VIII in the main text, since a local symmetry distortion could cause the Au^{2+}→Au^{3+} IVCT to become more localized.

Supplementary Discussion 4. Electronic structure evolution with pressure of $Cs_4Au^{II}Au^{III}_2Cl_{12}$

Structural relaxations above 20 GPa
In the structural relaxations obtained from our DFT-HSE calculations above 20 GPa, both $Au^{II}Cl_4$ and $Au^{III}Cl_4$ bond lengths are similarly compressed and become equivalent by 44 GPa (Figure 3D). However, the local structural distortion observed experimentally in Raman spectra above 21 GPa could not be observed due to the symmetry constraints of the calculations. Therefore, these results do not parallel experimental observations.

Band structures
Using the structural relaxations described in Section IV, we calculated electronic band structures and density of states at 0 GPa, 8 GPa, 16 GPa, 20 GPa, and 24 GPa with DFT using the HSE functional in VASP in order to understand the impact of compression on band dispersion and orbital composition of the IVCT in **1** (Figure 3E-F, Figure S14). At 0 GPa, **1** exhibits very flat bands with an indirect gap of 859 meV between degenerate valence band maxima (VBM) at P and Γ and degenerate conduction band minima (CBM) at X and N. The valence band and conduction band are composed of Au^{2+} and Au^{3+} d orbitals, respectively, with some Cl p-orbital contributions, as is expected for Class II mixed-valence. The direct gaps at X and N are nearly equivalent to the indirect gaps in energy at 860 meV, as is consistent with the initial report of **1**. Calculations of electronic structure at 8 GPa exhibit slightly more dispersion, and a reduced indirect bandgap of 543 meV from P to X with a slightly higher direct gap of 561 meV at X. At 16 GPa, a change in valence band alignment occurs resulting in the smallest indirect gap of 260 meV occurring from M to X and the smallest direct gap of 293 meV at P, though the orbital contributions remain the same. Conduction band alignment changes at 20 GPa, where the smallest indirect gap of 11 meV occurs from M to P and the smallest direct gap of 58 meV occurs at P. This same trend is repeated at 24 GPa with similar bandgap magnitudes. At 20 GPa and 24 GPa, there are substantial contributions of Au^{2+} and Au^{3+} d orbitals in both the valence and conduction bands as they approach the Fermi level, suggestive of strongly coupled metal centers with a very low energy barrier for optical IVCT.

Electron density map projections
Electron density maps, projected from the *ab* and *bc* planes (as defined in the single-crystal structure in Figure 1A) in the relaxed structures, were similarly computed using DFT-HSE to visualize the sub-orbital compositions of select bands for (i) identification of the orbitals involved in IVCT at ambient and high pressures, and (ii) distinction between inner-sphere and outer-sphere IVCT through visualization of the relative d-orbital energies compared to experimental absorption spectra (Figures S15-24). We calculated the density maps at the VBMs and CBMs of each band structure to visualize the Au d and Cl p orbitals involved in IVCT, and along the valence band, conduction band, and several other bands at Γ to visualize the relative d-orbital splitting energy of Au^{2+} and Au^{3+} with pressure. The VBMs and CBMs at 0 GPa are

composed of Au^{2+} $d_{x^2-y^2}$ and Au^{3+} $d_{x^2-y^2}$, respectively, with σ-bonded Cl p orbitals. This, along with the flat nature of the bands, indicate weak Class II behavior. At higher pressures, there is mixing of Au^{2+} and Au^{3+} contributions at the VBMs and CBMs, though the orbital assignments become less visually clear. As there is no shared halide between these metal orbitals, we invoke outer-sphere IVCT across B-site vacancies to describe the nature of the transition, as described in the main text. Notably, the σ-bonding Cl p-orbitals show density at the VBMs and CBMs, consistent with across-vacancy outer-sphere IVCT being the charge transfer mechanism in **1** (Figures 4-5, Figure S25).

To evaluate the validity of invoking outer-sphere IVCT and a VBM to CBM transition to describe the experimentally observed absorbance spectra, we visualized electron density maps of several lower-energy bands at Γ to understand the relative d-orbital energies with pressure (Figures S20-24). In the double perovskite $Cs_2Au^{I}Au^{III}X_6$, a VBM–1 to CBM inner-sphere transition is invoked to describe observed electronic transitions due to the orthogonality of the VBM and CBM (see Supplementary Discussion 1 for details).[8] However, this does not appear to be the case in **1** since the VBM–1 (Au^{2+} d_{xy}) and VBM–2 (Au^{2+} d_{z^2}), considered as the bands with substantial gaps in energy from the VBM, are *ca.* 2.8 eV and 3.2 eV lower in energy than the CBM at Γ, respectively. Given the experimental absorption onset of *ca.* 0.85 eV, the VBM to CBM outer-sphere IVCT is the only pathway within a reasonable energy range to describe the transition. Though the relative ordering of Au^{2+} d_{xy} and Au^{2+} d_{z^2} switches at higher pressures from contraction of the long bonds of the octahedra, the VBM–1 d_{z^2} band energy is always *ca.* 1 eV lower in energy than the CBM, which suggests that the outer-sphere Au^{2+} $d_{x^2-y^2}$ to Au^{3+} $d_{x^2-y^2}$ IVCT remains the lowest-energy observed transition at all pressures (Figure S25).

Effective masses

To computationally support the experimentally observed trend of differing carrier mobilities, we performed DFT-HSE calculations to estimate the electron and hole effective masses from the band structures in Figure S26. The analysis focused on comparing the curvature of the CBM and VBM, from which the effective masses were extracted. In the low-pressure regime, the conduction band shows a significantly larger curvature compared to the valence band, consistent with lower electron effective masses and hence higher electron mobilities, as observed experimentally. To calculate effective masses, second derivatives of the band energy at each VBM and CBM were tabulated as a 3 × 3 matrix. The eigenvalues of this correspond to the inverse of the effective masses along the crystallographic *a*, *b*, and *c* axes of our computational unit cell. The average of the absolute values of these eigenvalues can be interpreted as a measure for the inverse of the effective mass of the carrier at that band. As shown in Tables S4-7, these values decrease at higher pressures by *ca.* an order of magnitude. This trend is consistent with the increase in carrier mobilities with pressure seen in the two-carrier fits of Hall effect and magnetoresistance data.

Due to the size and complexity of the unit cell, calculations were performed using the HSE hybrid functional to correctly capture the local bonding environment. However, this came with substantial computational cost, which constrained our ability to use dense k-point meshes and precluded Wannier interpolation. Attempts to obtain maximally localized Wannier functions failed to converge reliably across all pressures, ruling out that method for high-resolution Fermi surface and curvature analysis.

Instead, we used a finite-difference approach to compute the effective mass tensor from local band curvature. The second derivatives of the band energies were approximated by symmetric finite differences around the band extrema. However, the accuracy of this approach is sensitive to the step size chosen for k-point displacement: large steps introduce systematic bias, while very small steps suffer from numerical noise due to finite precision in the calculated eigenvalues.

To assess the robustness of the effective mass trends, we computed values using three different k-point step sizes: 0.015, 0.025, and 0.035 (in units of 1/Bohr, Table S8). While the absolute values of the extracted

effective masses vary to some degree with step size, the qualitative trend is stable: hole effective masses remain larger than electron effective masses across the full pressure range, with the disparity being most pronounced at low pressures and decreasing at higher pressures. This is consistent between curvature trends in the calculated band structures and the experimental mobility data (Figure 8E-H), providing strong computational support for the hypothesis that hole mobilities are typically lower than electron mobilities. Given the intensity of these calculations, we view capturing trends as sufficient for this work, and reserve a more quantitative analysis of the effective masses in this material for a future study.

Supplementary Discussion 5. Evolution of the intervalence charge transfer (IVCT) potential energy curves of $Cs_4Au^{II}Au^{III}_2Cl_{12}$ with pressure

The evolution of the absorption onsets and activation energies of conductivity of **1** with pressure provides insight into the asymmetry of the IVCT. As described by Hush,[24] potential energy surfaces depicting IVCT are symmetric if the ΔG of IVCT is zero (the ground-state potential well minima are at equivalent energies) which results in a thermal IVCT barrier (E_a) to an optical IVCT barrier (E_{op}) ratio of 1:4. If asymmetric, this relationship will correspond to:

$$E_a = \frac{{E_{op}}^2}{4(E_{op} - E_0)}$$

where E_0 is the energy difference (ΔG) between ground-state potential curve minima before and after IVCT. The energy of the IVCT absorption peak maximum from diffuse reflectance data, and activation energy of conductivity from Arrhenius fits of variable-temperature conductivity, correspond to E_{op} and E_a, respectively (Table S9). In **1**, these values give an E_a to E_{op} ratio of 1:2.4, indicating a highly asymmetric IVCT at ambient pressure with an E_0 of 0.58 eV. As described in Table S9, linear fits of absorption onsets and Arrhenius fits of variable temperature conductivity can provide E_a and E_{op} values, respectively, to estimate this ratio and E_0 with pressure. We note that the use of IVCT absorption onset values, rather than the IVCT absorption peak maximum, causes the E_{op} values to be underestimates. This is unavoidable in high-pressure measurements of **1** because we cannot obtain a thin enough sample to prevent absorption saturation before the peak maximum of the IVCT feature. The use of the absorption onset value results in an ambient-pressure E_a to E_{op} ratio of **1** of 1:1.4 and an E_0 of 0.55 eV, indicating that the use of the absorption onset can indeed serve as an estimate of E_{op} with pressure.

Indeed, at 6 GPa, the E_a to E_{op} ratio of **1** becomes 1:1.6 and E_0 decreases to 0.16 eV, demonstrating that the IVCT becomes more symmetric with compression as the Au^{2+} and Au^{3+} coordination environments become more similar. Comparisons cannot be made at other pressures due to the low conductivity below 6 GPa that prevents collection of variable temperature conductivity, and the low absorption onsets above 6 GPa, which are occluded by diamond absorption and lattice modes so reliable absorption onset fits could not be obtained.

Supplementary Discussion 6. Variable-temperature structural and optical trends of $Cs_4Au^{II}Au^{III}_2Cl_{12}$

A low-temperature structural phase transition at high pressures could cause the observed curvature differences in our 2-K magnetoresistance data compared to data from 300-10 K. However, Raman spectra and optical absorption at 80 K, measured up to 22 GPa, exhibit identical pressure dependence as those from 300 K measurements. Additionally, variable-temperature PXRD at ambient pressure from 4 to 350 K exhibited no phase transitions, indicating that the tetragonal phase of **1** is robust with temperature in this range (Figures S34-35).

Supplementary References

(1) Mao, H. K.; Xu, J.; Bell, P. M. Calibration of the Ruby Pressure Gauge to 800 Kbar under Quasi-hydrostatic Conditions. *J. Geophys. Res.* **1986**, *91* (B5), 4673–4676. https://doi.org/10.1029/JB091iB05p04673.

(2) Wang, L.; Yang, W.; Xiao, Y.; Liu, B.; Chow, P.; Shen, G.; Mao, W. L.; Mao, H. Application of a New Composite Cubic-Boron Nitride Gasket Assembly for High Pressure Inelastic x-Ray Scattering Studies of Carbon Related Materials. *Revi. Sci. Instrum.* **2011**, *82* (7), 073902. https://doi.org/10.1063/1.3607994.

(3) Heyd, J.; Scuseria, G. E.; Ernzerhof, M. Hybrid Functionals Based on a Screened Coulomb Potential. *J. Chem. Phys.* **2003**, *118* (18), 8207–8215. https://doi.org/10.1063/1.1564060.

(4) Kresse, G.; Furthmüller, J. Efficient Iterative Schemes for *Ab Initio* Total-Energy Calculations Using a Plane-Wave Basis Set. *Phys. Rev. B* **1996**, *54* (16), 11169–11186. https://doi.org/10.1103/PhysRevB.54.11169.

(5) Blöchl, P. E. Projector Augmented-Wave Method. *Phys. Rev. B* **1994**, *50* (24), 17953–17979. https://doi.org/10.1103/PhysRevB.50.17953.

(6) A. Fonari; C. Sutton. Effective Mass Calculator, 2012. https://github.com/afonari/emc.

(7) Deschene, C. R.; Zwanziger, C.; Matheu, R.; Karunadasa, H. I. Mixed-Valence Halide Perovskites. *Coord. Chem. Rev.* **2025**, *539*, 216719. https://doi.org/10.1016/j.ccr.2025.216719.

(8) Kojima, N.; Kitagawa, H. Optical Investigation of the Intervalence Charge-Transfer Interactions in the Three-Dimensional Gold Mixed-Valence Compounds $Cs_2Au_2X_6$ (X = Cl, Br or I). *J. Chem. Soc., Dalton Trans.* **1994**, *3*, 327-331. https://doi.org/10.1039/dt9940000327.

(9) Jaffe, A.; Lin, Y.; Mao, W. L.; Karunadasa, H. I. Pressure-Induced Conductivity and Yellow-to-Black Piezochromism in a Layered Cu–Cl Hybrid Perovskite. *J. Am. Chem. Soc.* **2015**, *137* (4), 1673–1678. https://doi.org/10.1021/ja512396m.

(10) Matsushita, N.; Ahsbahs, H.; Hafner, S. S.; Kojima, N. Crystal Structure of Mixed-Valence Gold Compound, $Cs_2Au^{I}Au^{III}Cl_6$ up to 18 GPa. *Rev. High Press. Sci. Technol.* **1998**, *7*, 329–331. https://doi.org/10.4131/jshpreview.7.329.

(11) Tanino, H.; Syassen, K.; Wang, Z.; Hanfland, M.; Takahashi, K. The Mixed Valence Compound $Cs_2Au(I)Au(III)Cl_6$ under Pressure: Raman Modes and Optical Reflectivity. *High Press. Res.* **1990**, *3* (1), 183–185. https://doi.org/10.1080/08957959008246070.

(12) Liu, X. J.; Moritomo, Y.; Nakamura, A.; Kojima, N. Pressure-Induced Phase Transition in Mixed-Valence Gold Complexes $Cs_2Au_2X_6$ (X=Cl and Br). *J. Chem. Phys.* **1999**, *110* (18), 9174–9178. https://doi.org/10.1063/1.478839.

(13) Liu, X. J.; Moritomo, Y.; Nakamura, A.; Kojima, N. Pressure- and Photo-Induced Phase Transition in Mixed-Valence Gold Complexes. *Phase Transit.* **2001**, *74* (1), 51–92. https://doi.org/10.1080/01411590108224565.

(14) Robin, M. B.; Day, P. Mixed Valence Chemistry-A Survey and Classification. In *Advances in Inorganic Chemistry and Radiochemistry*; Elsevier, 1968; Vol. 10, pp 247–422. https://doi.org/10.1016/S0065-2792(08)60179-X.

(15) Keller, R.; Fenner, J.; Holzapfel, W. B. The Resistivity of Mixed Valence Compound $Cs_2Au_2Cl_6$ at High Pressure and Low Temperatures. *Mat. Res. Bull.***1974**, *9* (10), 1363–1369. https://doi.org/10.1016/0025-5408(74)90060-9.

(16) Kojima, N.; Fukuhara, F.; Kitagawa, H.; Takahashi, H.; Môri, N. Metallic Bipolaron Phase and Its Re-Entrant Phenomenon in the Au Mixed-Valence Compounds $Cs_2Au_2X_6$ (X=Cl, I) under High Pressure. *Synth. Met.* **1997**, *86* (1–3), 2175–2176. https://doi.org/10.1016/S0379-6779(97)81083-1.

(17) Sakata, M.; Itsubo, T.; Nishibori, E.; Moritomo, Y.; Kojima, N.; Ohishi, Y.; Takata, M. Charge Density Study under High Pressure. *J. Phys. Chem. Solids* **2004**, *65* (12), 1973–1976. https://doi.org/10.1016/j.jpcs.2004.08.028.

(18) Naumov, P.; Huangfu, S.; Wu, X.; Schilling, A.; Thomale, R.; Felser, C.; Medvedev, S.; Jeschke, H. O.; von Rohr, F. O. Large Resistivity Reduction in Mixed-Valent $CsAuBr_3$ under Pressure. *Phys. Rev. B* **2019**, *100* (15), 155113. https://doi.org/10.1103/PhysRevB.100.155113.
(19) Wang, S.; Hirai, S.; Shapiro, M. C.; Riggs, S. C.; Geballe, T. H.; Mao, W. L.; Fisher, I. R. Pressure-Induced Symmetry Breaking in Tetragonal $CsAuI_3$. *Phys. Rev. B* **2013**, *87* (5), 054104. https://doi.org/10.1103/PhysRevB.87.054104.
(20) Hafner, S. S.; Kojima, N.; Stanek, J.; Zhang, L. Divalent Gold in Perovskite Type $CsAuI_3$. *Phys. Lett. A* **1994**, *192* (5), 385–388. https://doi.org/10.1016/0375-9601(94)90224-0.
(21) Wang, S.; Kemper, A. F.; Baldini, M.; Shapiro, M. C.; Riggs, S. C.; Zhao, Z.; Liu, Z.; Devereaux, T. P.; Geballe, T. H.; Fisher, I. R.; Mao, W. L. Bandgap Closure and Reopening in $CsAuI_3$ at High Pressure. *Phys. Rev. B* **2014**, *89* (24), 245109. https://doi.org/10.1103/PhysRevB.89.245109.
(22) Kusmartseva, A. F.; Yang, M.; Arevalo-Lopez, A. M.; Kamenev, K. V.; Attfield, J. P. Pressure Suppression of Charge Order without Metallisation in $Cs_2Au_2I_6$. *Chem. Commun.* **2010**, *46* (36), 6681. https://doi.org/10.1039/c0cc01708f.
(23) Toby, B. H.; Von Dreele, R. B. *GSAS-II* : The Genesis of a Modern Open-Source All Purpose Crystallography Software Package. *J. Appl. Crystallogr.* **2013**, *46* (2), 544–549. https://doi.org/10.1107/S0021889813003531.
(24) Hush, N. S. Intervalence-Transfer Absorption. Part 2. Theoretical Considerations and Spectroscopic Data. In *Progress in Inorganic Chemistry*; Cotton, F. A., Ed.; Wiley, 1967; Vol. 8, pp 391–444. https://doi.org/10.1002/9780470166093.ch7.
